# sln-Topological Covalent Organic Frameworks with Shape Dimorphism and Dipolar Rotors

**DOI:** 10.1021/jacs.5c10010

**Published:** 2025-08-14

**Authors:** Xiaohan Wang, Takejiro Ogawa, Takuya Miyazaki, Kouki Kawamura, Atsuko Kosaka, Hiroaki Suzuki, Wang Zhang, Koji Yazawa, Yutaro Ogaeri, Takayuki Kamihara, Kiyohiro Adachi, Daisuke Hashizume, Yukihito Kondo, Takumi Sannomiya, Hidehiro Uekusa, Masaki Kawano, Ryosuke Takehara, Yoshiaki Shoji, Takanori Fukushima, Yoichi Murakami

**Affiliations:** † Laboratory for Zero-Carbon Energy, 693022 Institute of Science Tokyo, Tokyo 152-8550, Japan; ‡ Department of Mechanical Engineering, 693022 Institute of Science Tokyo, Tokyo 152-8552, Japan; § Laboratory for Chemistry and Life Science, 693022 Institute of Science Tokyo, Yokohama 226-8501, Japan; ∥ Department of Materials Science and Engineering, 693022 Institute of Science Tokyo, Yokohama 226-8501, Japan; ⊥ Department of Chemistry, 693022 Institute of Science Tokyo, Tokyo 152-8550, Japan; # 119855 JEOL Ltd., Akishima, Tokyo 196-8558, Japan; ¶ Facility Station Division, Core Facility Center, 693022 Institute of Science Tokyo, Yokohama 226-8501, Japan; ◆ RIKEN Center for Emergent Matter Science (CEMS), Wako, Saitama 351-0198, Japan; ○ Research Center for Autonomous Systems Materialogy (ASMat), 693022 Institute of Science Tokyo, Tokyo 226-8501, Japan; ▼ Department of Transdisciplinary Science and Engineering, 693022 Institute of Science Tokyo, Tokyo 152-8552, Japan

## Abstract

We report herein the first (i) covalent organic frameworks (COFs) with **sln** topology, (ii) drastic control of the shape of COF crystals with the same topology and chemical composition, (iii) insight that these different shapes are accompanied by conformational isomerism, and (iv) installation of dipolar rotors into three-dimensional (3D) COFs. We used a new building block, hexaarylbenzene with three dipolar 1,2-difluorophenyls. Depending on the solution composition, we generated two types of COF crystals with different shapes: hexagonal prism (**TK-COF-P**) and membrane (**TK-COF-M**). Although they exhibit distinctly different powder X-ray diffraction patterns, they are chemically identical. The structural determinations revealed that they have a low-symmetric, low-density **sln** topology that is yet to be reported for COFs. The two distinct shapes—shape dimorphism—is found to accompany conformational isomerism. Temperature-dependent dielectric and ^19^F NMR relaxation-time measurements reveal that the rotor motion is suppressed at room temperature, but the rotors respond to an external electric field at elevated temperatures owing to the high activation energy for rotation (∼20 kcal mol^–1^), which is desired for room-temperature applications including molecular memories. These outcomes have not only expanded the diversity of COFs but have also provided a foundation for installing external-field-responsive functions into COFs that have high thermal stability, which is expected to invoke broad applications.

## Graphical abstract

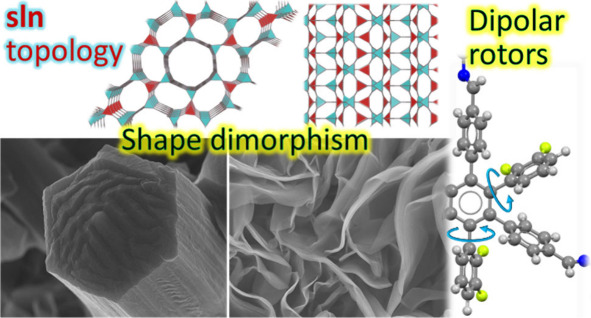

## Introduction

Covalent organic frameworks (COFs) emerged^1,2^ as designable porous solids following metal–organic frameworks (MOFs)^3^ in reticular chemistry.^4^ The higher robustness of COFs has expanded the realm of chemistry and invoked broad applications.^5–15^ Recently, in addition to two-dimensional (2D) COFs,^1,5–10^ 3D-COFs^2,7^ are burgeoning owing to their higher structural designability.^16–18^

However, the high energy (*ca*. 300–600 kJ mol^–1^)^19^ of covalent bonds has suppressed the diversity of COFs.^20,21^ So far, only *ca*. 30 topologies are known for 3D-COFs,^18^ whereas over 350 topologies are known for MOFs.^22^ Whereas isomerism^23,24^ and polymorphism^23,25^ have enriched the diversity of MOFs^23,24,26^ and 2D-COFs,^27–29^ isomerism in 3D-COFs is still rare.^30–34^

To facilitate applications, controlling materials’ shape is often desired. Previous efforts include the growth of 2D-COFs on a substrate^35–37^ and an interface between two liquids^38–42^ to form membranes. Exfoliations of single-crystal 2D-COFs into sheets were attained.^43,44^ However, active control of the macroscopic shape of COF crystals has yet to be achieved, which is more challenging and would bestow a much higher level of designability.

Furthermore, despite the broad applications proposed, a foundation for installing external-field-responsive functions into COFs has yet to be constructed. Such a foundation would open advanced application areas, such as molecular memories exploiting the high thermal stability of COFs. Although several studies^45–51^ installed dipolar rotors in MOFs, the rotors’ orientation could not be held at room temperature due to their relatively low activation energies (*E*_a_) for rotation (0.01 ≲ *E*_a_ ≲ 7 kcal mol^–1^), as they were studied mostly at low temperatures.^45–50^

Herein, we report multiple novelties that can address these issues. We report (i) the first COFs with **sln** topology (Figure 1a), (ii) the first drastic control of the shape of 3D-COF crystals with the same topology and chemical composition (prism vs. membrane, Figure 1b), (iii) the insight that this shape dimorphism is accompanied by *conformational isomerism*, and (iv) the first example of COFs in which dipolar rotors are installed.

**Figure 1:**
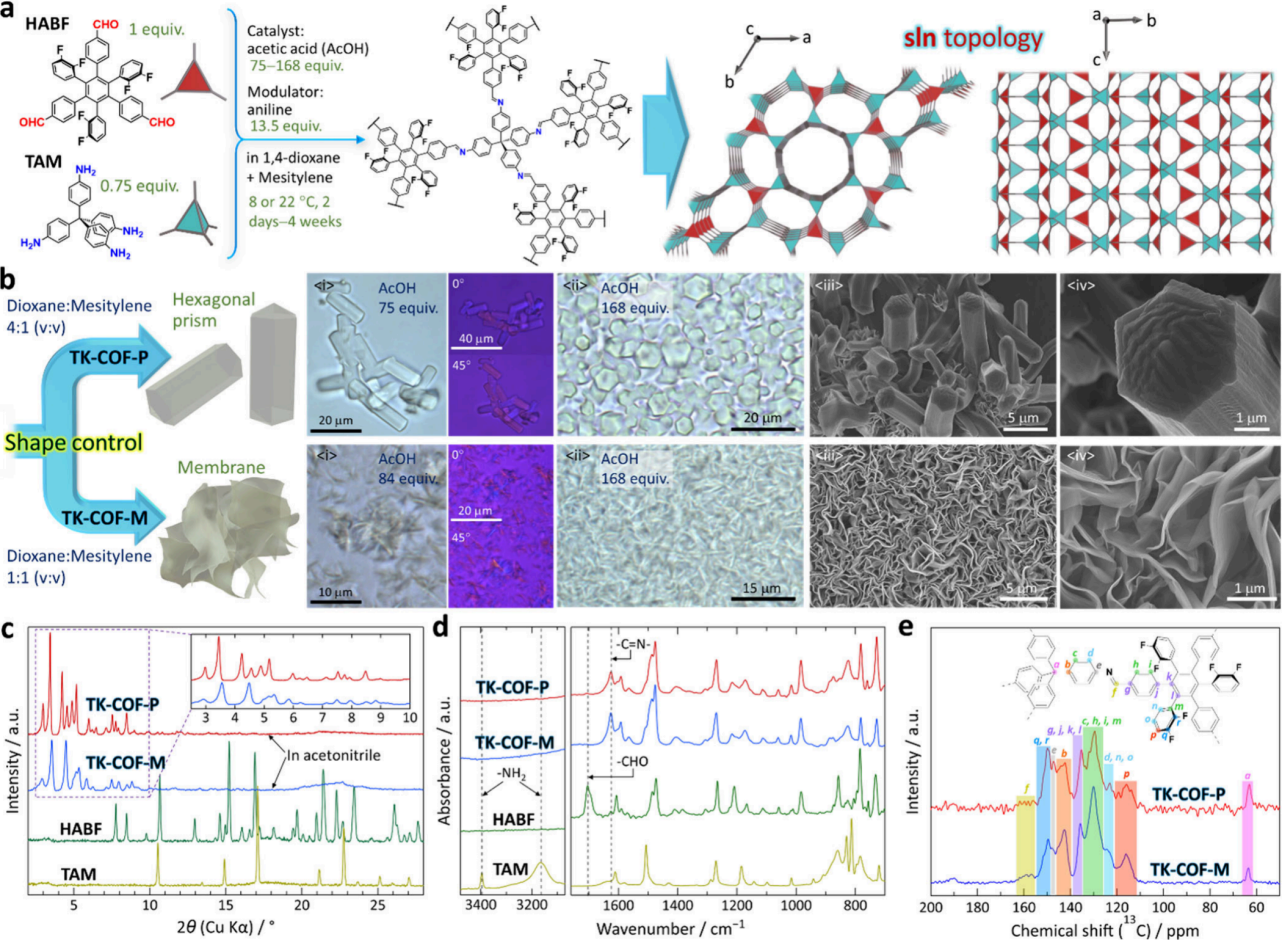
The **sln**-topological COFs with shape dimorphism and dipolar rotors. (a) Building-block molecules, **HABF** and **TAM**, and their reticulation into a 3D **sln** net. (b) Optical micrographs (panels <i> and <ii>) and SEM images (panels <iii> and <iv>) of **TK-COF-P** (hexagonal prism) and **TK-COF-M** (membrane). Solids in panels <i> and <ii> were generated with low and high AcOH concentrations, respectively. Panels <iii> and <iv> are low- and high-magnification SEM images, respectively, of the solids generated with a high AcOH concentration. The acquired (c) PXRD patterns, (d) FT-IR spectra, and (e) solid-state ^13^C CP/MAS NMR spectra.

## Results and Discussion

We combined triangular **HABF** (Figure 1a; Supporting Information, Section S1) and tetrahedral **TAM** (tetrakis­(4-aminophenyl)­methane, Figure 1a). **HABF** is a molecular memory unit^52^ in which three 1,2-difluorophenyl (DFP) groups as dipolar rotors and three aryl groups as suppressors against DFPs’ free-rotation are alternately positioned around the central benzene. The dynamics of the molecular unit of **HABF** without aldehydes was previously investigated in a toluene solution, revealing that the rotors had a sufficiently large *E*_a_ (*ca*. 22 kcal mol^–1^) for holding the rotors’ orientation at room temperature.^52^ However, when in the form of a molecular solid, the steric hindrance in the dense solid hampered the rotors’ flip motion.^52^ Furthermore, the van der Waals condensation lacked thermal stability. As shown below, we evolve this immature concept by bringing it to a COF platform, aiming to ensure an adequate microspace for the rotors to flip and thermal stability.

After growth at 8 or 22 °C (Section S1.3 for details; Tables S1 and S2 for the conditions), we generated two kinds of solids with distinctly different shapes (size: 5–30 μm) depending on the solvent (Figure 1b). The use of dioxane:mesitylene = 4:1 and 1:1 yielded hexagonal prisms (**TK-COF-P**) and membranes (**TK-COF-M**), respectively; the latter was in a gathered petal-like form. Polarizing microscopy indicated single crystallinity of each shape domain (<i> in Figure 1b). An increase of AcOH increased the amount of the solid (<i> vs. <ii> in Figure 1b). Scanning electron microscopy (SEM, <iii> and <iv> in Figure 1b; full-size images: Figures S10–S12) revealed that the prisms in vacuum were bent or twisted, implying their softness and low density, whereas the membranes were so thin (≤50 nm) that some petals were translucent in the SEM images. Notably, the replacement of F in **HABF** by H resulted in amorphous or poorly crystalline solids (Figures S13–S15); this may be ascribed to the assistive effect of F on the structure formation of COFs.^53–55^

**TK-COF-P** and **-M** showed distinctly different powder X-ray diffraction (PXRD) patterns (Figure 1c, in acetonitrile; see Section S2.4 for the method). These PXRD patterns were highly reproducible (*e*.*g*., Figure S23) and show that the amount of **TK-COF-M** contained in the **-P** batch (and *vice versa*) was sufficiently small. Notably, these two kinds of solids showed the same Fourier transform infrared (FT-IR) spectra (Figure 1d), ^13^C cross-polarization magic-angle-spinning (CP/MAS) NMR spectra (Figure 1e), and results of elemental analysis (EA, Table S3), indicating their chemical identity. The FT-IR spectra showed the disappearance of amine and aldehyde and the emergence of imine, confirming the completion of the polycondensation between **HABF** and **TAM** (see Figure 1a).

Because of the low density of these crystals (*ca*. 0.2 g cm^–3^, see below) and the shape distortions seen in vacuum (*e*.*g*., <iii> in Figure 1b for **TK-COF-P**), the solvent removal diminished rigorous periodicity and hence weakened the PXRD patterns (Figure S16), as reported for MOFs^56^ and COFs.^33^ However, the framework integrity was retained after the solvent removal, as has been evidenced by the aforementioned FT-IR, ^13^C CP/MAS NMR, and EA results, all of which were measured for the crystals in the dried state.

To determine the structures, we conducted PXRD using synchrotron radiation^57^ (Sections S2.4 and S4.1) on **TK-COF-P** and **-M** in acetonitrile. Then, using the *Reticular Chemistry Structure Resource* database,^58^ we found ten candidate topologies (**bor**, **bor-c**, **ctn**, **ctn-c**, **ept**, **ofp**, **jaj**, **asn**, **jcy**, and **sln**; Figure S24) that can be formed by combining tetrahedral and trigonal building blocks; the criterion for excluding further alternative topologies is provided in Section S4.2. For these candidate topologies, we constructed structural models by connecting **HABF** and **TAM** (Section S4.2). Consequently, only the simulated PXRD pattern generated from the **sln**-topological model agreed excellently with the measured PXRD pattern of **TK-COF-P**, whereas the other candidate topologies differed substantially from the measured PXRD patterns (Figures S25 to S28).

Then, after the Pawley refinement of the unit cell (Figure S29), we conducted Rietveld refinement^59^ to determine the structure of **TK-COF-P** (Section S4.3), which resulted in small *R*_wp_ (1.40%) and *R*_p_ (0.97%) values (Figures 2a and 2b). The unit cell parameters were *a* = *b* = 59.660(3) Å, *c* = 23.024(2) Å, α = β = 90°, γ = 120°, with the *P*6_3_ space group (Table S6 for the atomic coordinates).

**Figure 2:**
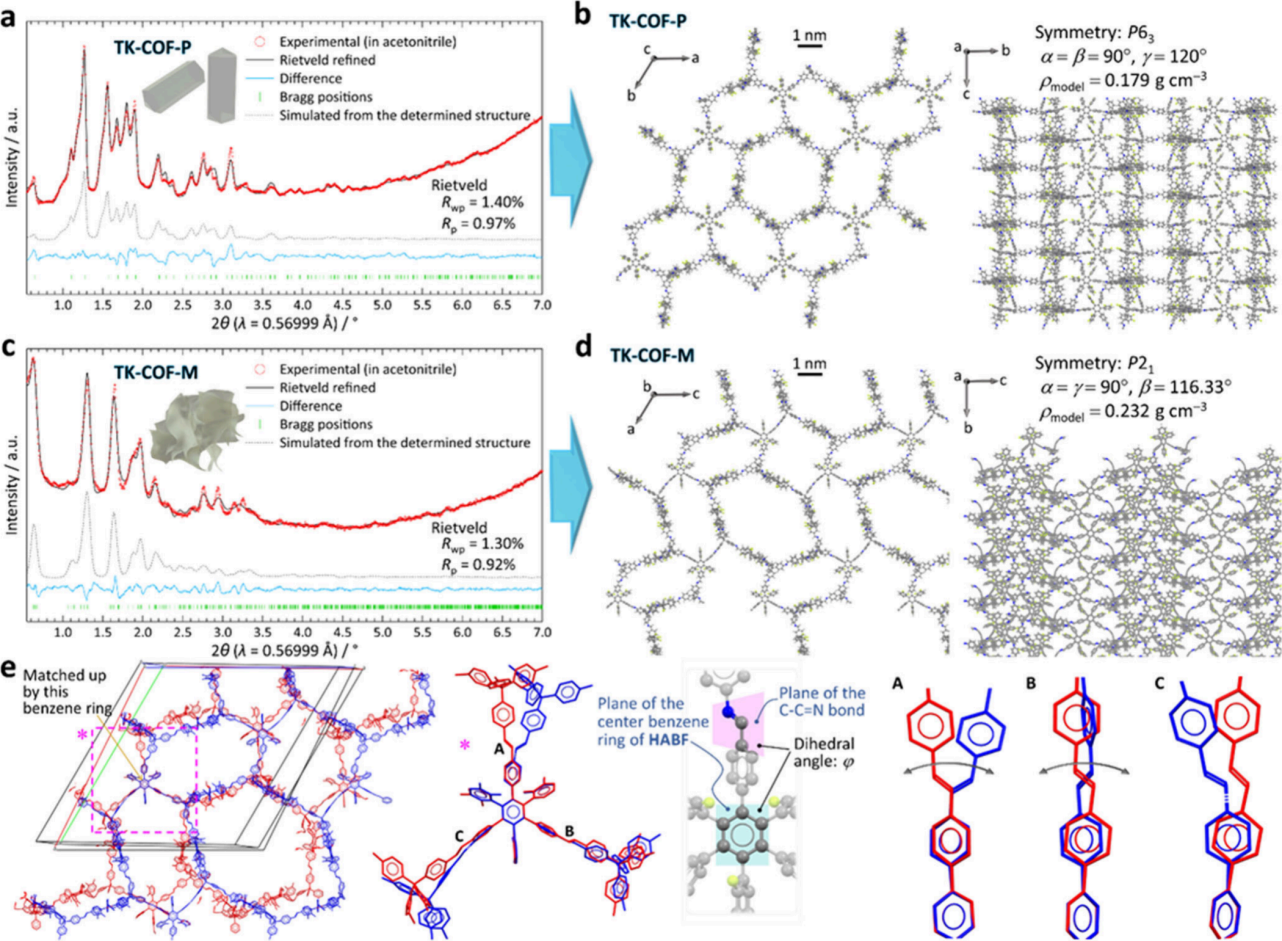
Determined structures of **TK-COF-P** and **-M**. (a and c) High-resolution synchrotron PXRD patterns and the results of the Rietveld analyses. See Sections S2.4 and S4 for the details of the measurement and analyses, respectively. (b and d) The structures determined by the Rietveld refinements. (e) The difference in the conformations of **TK-COF-P** (red) and **TK-COF-M** (blue), in which the central benzene rings of **HABF** are overlaid. The dihedral angles are summarized in Table S8.

Because the lowest peak at 2θ = 0.63° was commonly observed for both COFs (Figures 2a and 2c), we inferred that **TK-COF-M** also has **sln** topology but a different symmetry. Then, we tested a lower symmetry of *P*2_1_, which resulted in an excellent Pawley refinement of the cell (Figure S31b). Finally, we conducted Rietveld refinement (Section S4.4), which resulted in small *R*_wp_ (1.30%) and *R*_p_ (0.92%) values (Figures 2c and 2d). The unit cell parameters were *a* = 58.261(7) Å, *b* = 17.392(3) Å, *c* = 60.335(6) Å, α = 90°, β = 116.332(10)°, γ = 90° (Table S7 for the atomic coordinates). Consequently, we found that **TK-COF-P** and **-M** have unprecedented **sln** topology (Figures S30 and S32 for the topology check). One advantage of **sln** topology is its inherently noninterpenetrated net,^58^ which resulted in low density (ρ_model_ = 0.179 and 0.232 g cm^–3^, respectively) and softness (Figure 1, SEM). Notably, the formation of nets with low symmetry, such as **sln** topology (transitivity = 3343),^58^ is rather challenging.^2,7^

We found that **TK-COF-P** and **-M** are examples of *conformational isomerism* ^23,24,26^ (Figure 2e), which demands identical connectivity and composition but allows twists, bends, or rotation of bonds.^24,26^ The comparison revealed that they had, as a major difference, different dihedral angles between the center benzene ring of **HABF** and the imine C–C=N bond (φ in Figure 2e; Table S8). Conformational isomerism in COFs has rarely been studied or remarked on explicitly so far.^34,60^ The transformation of **TK-COF-P** into **-M** over a long time in the growth solution (Figure S34) and our energy calculations (Sections S5.2, S5.3) reveal that **TK-COF-M** is a thermodynamic product whereas **TK-COF-P** is a kinetic product; this may explain why a higher (lower) mesitylene ratio resulted in thermodynamic (kinetic) product based on solvent-polarity-dependent thermodynamics (Section S5.4). The reason for the use of 8 °C for growing **TK-COF-P** (Table S2) was to slow down the conversion into **TK-COF-M** in the growth solution.

Some SEM images showed that membranes were grown from the side of the hexagonal prisms in parallel to the prism axis (Figure S42), suggesting that the surface of **TK-COF-M** is parallel to the *b*-axis (see Figure 2d). Among the three possible surfaces, (100), (001), and (101̅), we surmised that (101̅) is the most plausible because it has the lowest bond density of the three (Figure 3a). The high-resolution transmission electron microscope (HR-TEM) image normal to the membrane showed a stripe period of 3.3–3.4 nm, which satisfactorily agrees with the stripe period on the (101̅) plane of the determined structural model (Figure 3b, Figure S33).

**Figure 3:**
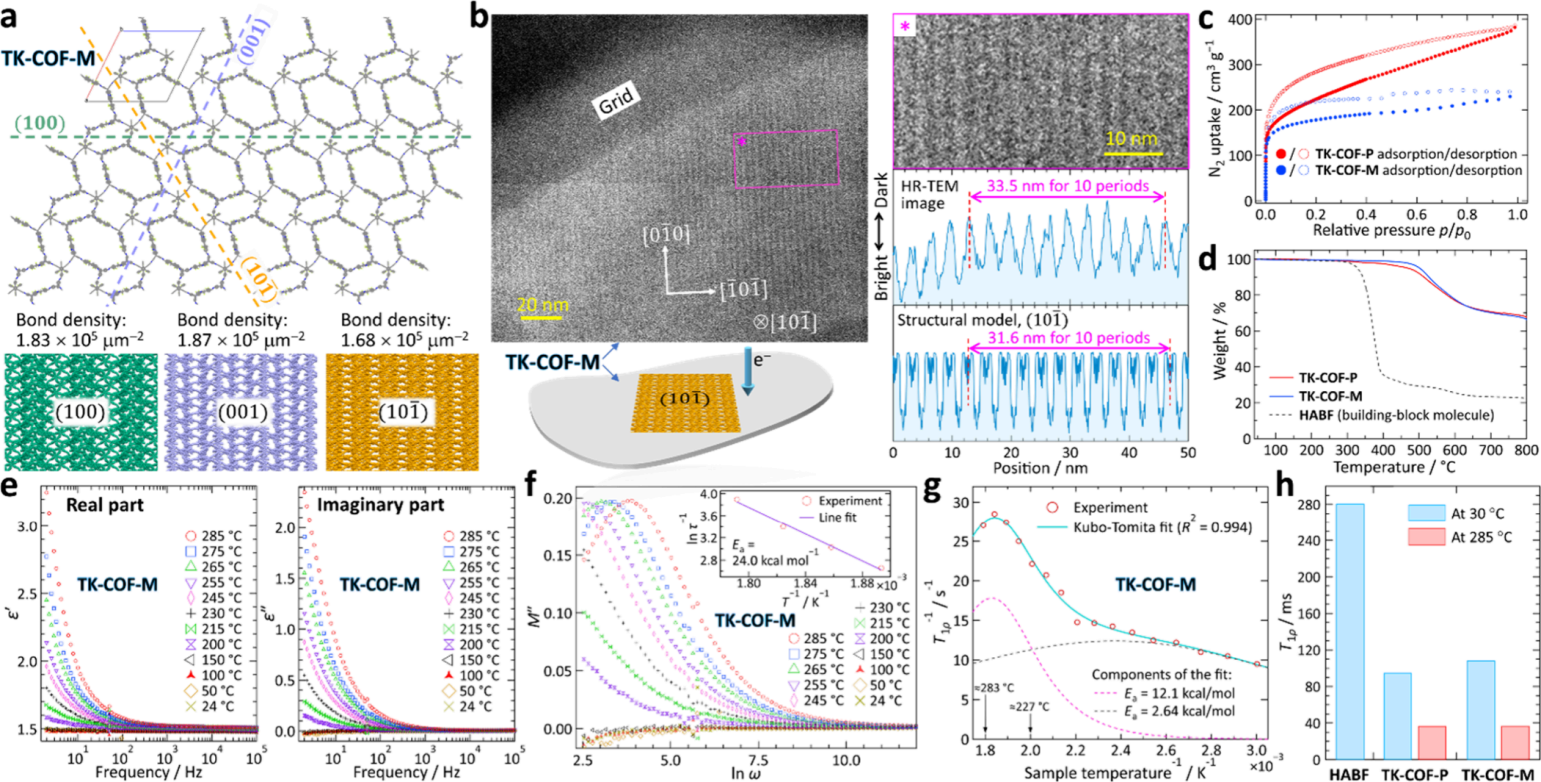
Characterizations of the COFs. (a) Three candidate crystallographic planes for the surface of **TK-COF-M**. The structure on each plane was made by slicing the crystal structure shown in Figure 2d. (b) HR-TEM images of **TK-COF-M** and the comparison with the (101̅) plane of the structure determined. (c) N_2_ adsorption–desorption isotherms at 77 K. (d) TGA data acquired in N_2_ flow. (e) Dielectric responses of **TK-COF-M** (Section S3.5 for **TK-COF-P**). (f) Dependence of the imaginary part of electric modulus *M*″ on the angular frequency ω = 2π*f* and temperature (Section S6.3 for **TK-COF-P**; see also Section S6.2). Inset: Arrhenius plot for τ^–1^ (= 2π*f*_m_) vs *T* ^–1^. (g) Temperature dependence of ^19^F *T*_1ρ_^–1^ (Figure S55 for **TK-COF-P**; Section S7.2 for raw data) and the Kubo–Tomita fit (Section S7.5 for details). (h) Comparison of ^19^F *T*_1ρ_ of **HABF**, **TK-COF-P**, and **TK-COF-M** (Section S7.2 for raw data).

For the rotors in **TK-COF-P** and **-M** to rotate, adequate microspace must exist in the dried state. The type-I nitrogen adsorption isotherms at 77 K (Figure 3c) and moderately high calculated surface areas (794 and 609 m^2^ g^–1^ for **TK-COF-P** and **-M**, respectively; Figure S17) confirmed the porosities or voids in the dried state. The higher surface area of **TK-COF-P** could be ascribed to its lower density. High thermal stability of these COFs to 400 °C was found (Figure 3d).

To assess the dynamics of the rotors, we conducted temperature-dependent dielectric spectroscopy (Figure 3e; Section S3.5 for **TK-COF-P**), where the maximum allowable temperature was 285 °C (see Section S2.10). The results were reproducible because of the *in-vacuo* experiments and samples’ high thermal stability. Very small temperature dependences were found in the real (*ε*′) and imaginary (*ε*″) parts of the permittivity below 150 °C, whereas *ε*′ and *ε*″ started to increase above 200 °C, implying the stability of the DFP rotors against reorientation under ambient temperatures. They rapidly decreased with increasing frequency, indicating that the pertinent kinetics—considered to be the rotors’ flipping motion—is substantially slow.

Notably, dielectric relaxation studies can be performed even in the absence of a well-defined peak in *ε*″ from the electric modulus analysis^61,62^ (Section S6.1). The imaginary part of the dielectric modulus (*M*″) was plotted against the frequency for **TK-COF-M** (Figure 3f), where the maxima were found in the low-frequency region (<10 Hz) and the frequency at the maxima (*f*_m_) depended on the temperature. Because (2π*f*_m_)^−1^ = ω_m_^–1^ represents the characteristic relaxation time τ,^61,62^ the activation energy *E*_a_ was obtained from the Arrhenius plot^61,62^ (inset of Figure 3f) to be *E*_a_ = 24.0 kcal mol^–1^; this is very close to *E*_a_ ≈ 22 kcal mol^–1^ reported previously for the DFP rotors in the molecular unit of **HABF** without aldehydes in a toluene solution.^52^ Such peaks were, however, unfound in *M*″ for **TK-COF-P** (Figure S45) probably because *f*_m_ existed below the lower frequency limit (2 Hz) of our experimental system. We surmise that the dependence of the dielectric response on the electric field direction is weak because **TK-COF-P** has two crystallographically nonequivalent **HABF** units that are almost perpendicularly oriented with each other (Figure 2b), and **TK-COF-M** has four crystallographically nonequivalent **HABF** units that orient in different directions (Figure 2d).

Although only small temperature dependences were found above the kHz region, we conducted temperature-dependent ^19^F solid-state NMR measurements in which the upper allowable temperature of the sample was strictly limited to 285 °C (see Section S7.1). We used the spin–lattice relaxation times in the rotating frame (*T*_1ρ_), which can provide information on molecular motions in the kHz range,^63^ rather than those in the laboratory frame (*T*_1_), which is suitable for studying fast dynamics in the ∼100 MHz range (Section S7.2 for *T*_1_ and *T*_1ρ_ data); *T*_1ρ_ was previously chosen to study the rotations of DFP-based rotors.^64^ Because of the large energy barrier for rotation and the strict upper temperature limit (285 °C) of the setup, only the *T*_1ρ_^–1^ vs *T* ^–1^ data for **TK-COF-M** could be fit using the Kubo–Tomita function (Figure 3g; Section S7.5; Figure S55 for data for **TK-COF-P**). The fit yielded an *E*_a_ of *ca*. 12 kcal mol^–1^, which is almost half of the *E*_a_ determined above, perhaps owing to the inaccuracy of the fit. Nevertheless, such *E*_a_ values (12–24 kcal mol^–1^) are far higher than the thermal energy at room temperature (*ca*. *RT*_300 K_ = 0.6 kcal mol^–1^) and *E*_a_ values in the aforementioned MOFs,^45–50^ supporting the stability against reorientation under ambient temperatures. Finally, at 30 °C (*RT*_303 K_ ≪ *E*_a_, *i*.*e*., only oscillation without flip), a much shorter *T*_1ρ_ in the COFs than in the molecular solid of **HABF** (Figure 3h) suggests lesser steric hindrance in the COFs owing to ample microspace around the rotors; a longer *T*_1ρ_ for **TK-COF-M** than **-P** at 30 °C may be related to the more remarkable steric hindrance caused by the more distorted and denser skeleton of **-M** than that of **-P**.

Finally, we mention that the peak positions in the PXRD patterns of **TK-COF-P** and **-M** in the dried state at 25 and 285 °C showed no noticeable differences (Figure S19). This indicates that the temperature-induced variance in the framework structure was minor and hence not the major mechanism of the temperature-dependent kinetics of the rotors observed above. We also checked that the structures were maintained after heating to 285 °C by comparing the PXRD patterns acquired before and after heating (Figure S20). From the comparisons of the nonbond energies of **TK-COF-P/-M** and those of hypothetical H-substituted **TK-COF-P/-M** (Section S5.6), the hydrogen-bond interaction of F with H in the present COFs is not considered to be significant, at least to the extent of affecting the dynamics of the present DFP rotors.

## Conclusions

To summarize, we have expanded the diversity of COFs by discovering **sln**-topology and shape dimorphism. By orderly allocation of the dipolar rotors on the low-density **sln** skeleton, we have simultaneously achieved an adequately large *E*_a_ and microspace around the rotors, by which the rotation is suppressed at room temperature, but the rotors can respond to an external electric field at elevated temperatures, resolving the issue suffered by molecular solid systems. Thus, this report has provided a prototypical foundation for installing external-field-responsive functions into COFs that excel in thermal stability, which is expected to invoke broad applications.

## Supplementary Material



## Notes

Additional details of materials and methods for COF synthesis, methods of sample characterizations, supporting results of sample characterizations, structural determination of **TK-COF**s by Rietveld refinement, discussion of energetic stabilities of **TK-COF**s, electric modulus analysis and results, and dipolar rotor dynamics investigated by temperature-dependent solid-state ^19^F NMR measurements (PDF)

## ■

Formerly Tokyo Institute of Technology. The university name has changed to Institute of Science Tokyo since October 1, 2024.
