# Supplementary material for: sln-Topological Covalent Organic Frameworks with Shape Dimorphism and Dipolar Rotors
Source: J Am Chem Soc. 2025 Aug 14;147(34):31204–11. doi: 10.1021/jacs.5c10010 (PMC12395412; doi:10.1021/jacs.5c10010)
Supplement: Supplementary file 1 [file ja5c10010_si_001.pdf]

Supporting Information

sln-Topological Covalent Organic Frameworks with Shape Dimorphism and Dipolar Rotors

Xiaohan Wang,[†,1] Syunto Goto,[†,1,2] Takejiro Ogawa,[3,4] Takuya Miyazaki,[3,4] Kouki Kawamura,[3,4] Atsuko Kosaka,[3] Hiroaki Suzuki,[5] Wang Zhang,[5] Koji Yazawa,[6] Yutaro Ogaeri,[6] Takayuki Kamihara,[7] Kiyohiro Adachi,[8] Daisuke Hashizume,[8] Yukihito Kondo,[4] Takumi Sannomiya,[4] Hidehiro Uekusa,[5] Masaki Kawano,[5] Ryosuke Takehara,[3,4,9] Yoshiaki Shoji,[3,4,9] Takanori Fukushima,[*,3,4,9] and Yoichi Murakami[*,1,2,10]

[1] Laboratory for Zero-Carbon Energy, Institute of Science Tokyo,[‡] Tokyo 152-8550, Japan.
[2] Department of Mechanical Engineering, Institute of Science Tokyo,[‡] Tokyo 152-8552, Japan.
[3] Laboratory for Chemistry and Life Science, Institute of Science Tokyo,[‡] Yokohama 226-8501, Japan.
[4] Department of Materials Science and Engineering, Institute of Science Tokyo,[‡] Yokohama 226-8501, Japan
[5] Department of Chemistry, Institute of Science Tokyo,[‡] Tokyo 152-8550, Japan.
[6] JEOL Ltd., Akishima, Tokyo 196-8558, Japan.
[7] Facility Station Division, Core Facility Center, Institute of Science Tokyo,[‡] Yokohama 226-8501, Japan.
[8] RIKEN Center for Emergent Matter Science (CEMS), Wako, Saitama 351-0198, Japan.
[9] Research Center for Autonomous Systems Materialogy (ASMat), Institute of Science Tokyo, 226-8501, Japan.
[10] Department of Transdisciplinary Science and Engineering, Institute of Science Tokyo,[‡] Tokyo 152-8552, Japan.

* Corresponding authors: murakami.y.af@m.titech.ac.jp, fukushima@res.titech.ac.jp

[†] These authors contributed equally to this work.

[‡] Formerly "Tokyo Institute of Technology," which turned to "Institute of Science Tokyo" on October 1, 2024 due to the merger with a medical university.

Table of Contents

Section S1. Materials and methods for COF synthesis

S1.1 Chemicals from suppliers

S1.2 Synthesis of **HABF** (Scheme S1, Figures S1−S6)

S1.3 Synthesis of **TK-COF-P** and **-M** (Figure S7, Tables S1 and S2)

S1.4 Elemental analyses (EA) (Table S3)

Section S2. Methods of sample characterization

S2.1 Washing and drying procedures before characterization

S2.2 Optical microscopy

S2.3 Scanning electron microscopy (SEM)

S2.4 Powder X-ray diffraction (PXRD) measurements

S2.5 Fourier-transform infrared (FT-IR) spectroscopy

S2.6 ^{13}C solid-state nuclear magnetic resonance (ss-NMR) spectroscopy

S2.7 High-resolution transmission electron microscopy (HR-TEM)

S2.8 Nitrogen adsorption isotherm measurements

S2.9 Thermogravimetric analysis (TGA)

S2.10 Temperature-dependent dielectric response measurements (Figures S8, S9)

Section S3. Supporting results of sample characterization

S3.1 Full-size SEM images (Figures S10−S12)

S3.2 Synthesis using building-block molecules in which the F atoms in **HABF** were replaced by H atoms (Scheme S2, Table S4, Figures S13−S15)

S3.3 PXRD patterns of **TK-COF-M** before and after drying (Figure S16)

S3.4 BET surface area plots (Figure S17)

S3.5 Temperature-dependent dielectric response of **TK-COF-P** (Figure S18)

S3.6 Temperature-dependent PXRD patterns (Figure S19)

S3.7 Comparison of PXRD patterns before and after heating to 285 °C (Figure S20)

Section S4. Structural determination of TK-COF-P/-M by Rietveld refinement

S4.1 Synchrotron PXRD patterns (Figures S21−S23)

S4.2 Possible framework topologies and their structural models (Table S5, Figures S24−S28)

S4.3 Structural determination of **TK-COF-P** (Figures S29 and S30, Table S6)

S4.4 Structural determination of **TK-COF-M** (Figures S31 and S32, Table S7)

S4.5 Comparison of the dihedral angles (Table S8)

S4.6 Comparison of stripe periods in the HR-TEM image with the structural models (Figure S33)

Section S5. Discussion of energetic stabilities of TK-COF-P/-M

S5.1 Transformation of **TK-COF-P** to **-M** after a long retention in solution (Figure S34)

S5.2 Energy calculations of structural moieties (Figures S35−S40)

S5.3 Total energies of **TK-COF-P** and **-M** (Table S9)

S5.4 Explanation on the evolution into different morphologies depending on the solvent ratio (Figure S41)

S5.5 SEM images showing membranes grown from the side of prisms (Figure S42)

S5.6 Comparison of non-bond energies of **TK-COF-P/-M** and those of hypothetical H-substituted **TK-COF-P/-M** (Tables S10, S11)

Section S6. Electric modulus analysis and results

S6.1 Formulation of electric modulus analysis

S6.2 Temperature and frequency dependences of M' and M'' for **TK-COF-M** (Figures S43, S44)

S6.3 Temperature and frequency dependences of M' and M'' for **TK-COF-P** (Figures S45, S46)

Section S7. Dipolar rotor dynamics investigated by temperature-dependent solid-state ^{19}F NMR measurements

S7.1 Measurement methods and samples

S7.2 Raw data from ^{19}F spin-lattice relaxation measurements for T_1 and $T_{1\rho}$ (Figures S47−S51)

S7.3 Temperature dependences of T_1 and $T_{1\rho}$ (Figures S52−S55)

S7.4 Discussion of the temperature dependence of signal linewidth (Figure S56)

S7.5 Procedure of Kubo-Tomita analysis and obtained fit parameters (Table S12)

References

Section S1. Materials and methods for COF synthesis

S1.1 Chemicals from suppliers

Tetrakis(4-aminophenyl)methane (**TAM**; > 95%, NMR) was purchased from Accela ChemBio in the USA. 1,4-Dioxane (99.5%), mesitylene (97%), acetic acid (99.5%), acetonitrile (99.5%), acetone (99.5 %), and toluene (99.5 %) were supplied from FUJIFILM Wako Chemicals in Japan. Aniline (99.5%) was purchased from Sigma-Aldrich in the USA. Methyltrioctylammonium bis(trifluoromethanesulfonyl)imide ($[N_{8881}][NTf_2]$, 99%) was obtained from IoLiTec in Germany. These chemicals were used without further purification.

S1.2 Synthesis of **HABF**

A hexaarylbenzene derivative, 2',4',6'-tris(2,3-difluorophenyl)-5'-(4-formylphenyl)-[1,1':3',1''-terphenyl]-4,4''-dicarbaldehyde (**HABF**), was synthesized according to Scheme S1 below and the following procedures.

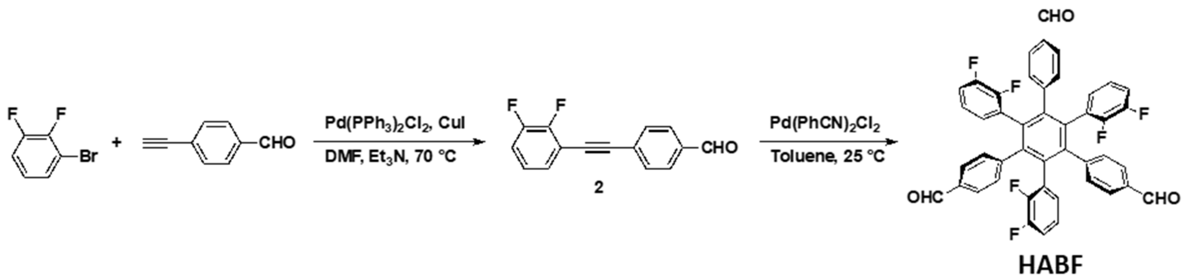

Scheme S1. Synthesis of **HABF**.

<u>Methods</u>

Column chromatography was carried out using Silica Gel 60N (particle size: 63–210 μm). Infrared (IR) spectra were recorded at 25 °C on a JASCO model FT/IR-660Plus Fourier-transform IR spectrometer. Nuclear magnetic resonance (NMR) spectroscopy measurements were carried out using a Bruker model AVANCE-400 spectrometer (^{1}H: 400.0 MHz, ^{13}C: 100.6 MHz, ^{19}F:

376.3 MHz) or a Bruker model AVANCE III HD-500 spectrometer (^{1}H: 500.0 MHz, ^{13}C: 125.7 MHz, ^{19}F: 470.4 MHz). Chemical shifts (δ) are expressed relative to the resonance of the residual non-deuterated solvent for ^{1}H ($CDCl_3$: $^{1}H(\delta)$ = 7.26 ppm), the resonance of the residual solvent for ^{13}C ($CDCl_3$: $^{13}C(\delta)$ = 77.16 ppm), and the resonance of CF_3CO_2H as an external standard for ^{19}F (CF_3CO_2H: $^{19}F(\delta)$ = –76.55 ppm). Absolute values of the coupling constants are given in Hertz (Hz), regardless of their sign. Multiplicities are abbreviated as singlet (s), doublet (d), triplet (t), quartet (q), multiplet (m), and broad (br). Mass spectrometry measurements were carried out using a Bruker model micrOTOF II mass spectrometer equipped with an atmospheric pressure chemical ionization (APCI) probe.

Synthesis

Compound 2. Under a nitrogen atmosphere at 25 °C, $Pd(PPh_3)_2Cl_2$ (1.17 g, 1.67 mmol) and CuI (568 mg, 2.98 mmol) were added to a DMF/Et_3N solution (175 mL, 4/3 v/v) of a mixture of 1-bromo-2,3-difluorobenzene (4.0 mL, 35.7 mmol) and 4-ethynylbenzaldehyde (3.55 g, 237.3 mmol), and the reaction mixture was stirred at 70 °C for 18 h and then evaporated to dryness under reduced pressure. The residue was subjected to column chromatography (SiO_2, CH_2Cl_2/hexane 1/2 v/v) to allow isolation of the product (3.20 g, 13.2 mmol) as a pale-yellow powder in 48% yield. FT-IR (KBr): ν (cm^{-1}) 2835, 2740, 1701, 1621, 1600, 1561, 1508, 1481, 1383, 1334, 1301, 1287, 1267, 1222, 1207, 1187, 1174, 1158, 1060, 984, 825, 785, 756, 714, 666, 634. ^{1}H NMR (400 MHz, $CDCl_3$, 25 °C, Figure S1): δ (ppm) 10.04 (s, 1H), 7.89 (d, J = 8.2 Hz, 2H), 7.71 (d, J = 8.2 Hz, 2H), 7.32–7.28 (m, 1H), 7.23–7.16 (m, 1H), 7.11–7.06 (m, 1H). ^{13}C NMR (100 MHz, $CDCl_3$, 25 °C, Figure S2): δ (ppm) 191.4, 151.0 (dd, J_{CF} = 250.0, 12.0 Hz), 150.6 (dd, J_{CF} = 250.0, 12.0 Hz), 135.9, 132.3, 129.6, 128.6, 128.3 (d, J = 3.7 Hz), 124.1 (dd, J_{CF} = 7.3, 5.1 Hz), 118.2 (d, J_{CF}

= 17.6 Hz), 113.4 (d, J_{CF} = 12.5 Hz), 94.4 (d, J_{CF} = 3.7 Hz), 85.2 (d, J_{CF} = 5.1 Hz). ^{19}F NMR (376 MHz, $CDCl_3$, 25 °C, Figure S3): δ (ppm) –134.3, –137.1. APCI-TOF mass: calcd. for $C_{15}H_8F_2O$ $[M]^+$: m/z = 242.0538; found: 242.0401.

Compound HABF. Under a nitrogen atmosphere at 25 °C, $Pd_2(PhCN)_2Cl_2$ (642 mg, 1.64 mmol) was added to a toluene solution (48 mL) of **2** (3.20 g, 13.2 mmol), and the resulting mixture was stirred at 25 °C for 24 h. Additional $Pd_2(PhCN)_2Cl_2$ (570 mg, 1.46 mmol) was added to the reaction mixture, and the mixture was stirred at 25 °C for 42 h, and then evaporated to dryness under reduced pressure. The residue was subjected to column chromatography (SiO_2, AcOEt/hexane 1/2 v/v) to afford a yellow solid containing **HABF**. The mixture was subjected to column chromatography (SiO_2, CH_2Cl_2/hexane 3/1 v/v) to allow isolation of **HABF** (101 mg, 0.139 mmol) as a pale-yellow powder in 3.2% yield. FT-IR (KBr): ν (cm^{-1}) 3446, 3047, 2825, 2774, 1703, 1625, 1606, 1590, 1570, 1487, 1474, 1410, 1386, 1306, 1267, 1209, 1167, 1114, 1059, 984, 875, 857, 823, 806, 785, 732, 602. ^{1}H NMR (500 MHz, $CDCl_3$, 25 °C, Figure S4) δ (ppm) 9.28 (s, 3H), 7.57 (dd, J = 8.0, 1.5 Hz, 3H), 7.50 (dd, J = 8.0, 1.5 Hz, 3H), 7.32 (d, J = 8.0 Hz), 7.06 (dd, 8.0, 1.5 Hz, 3H), 6.90–6.75 (m, 3H), 6.70–6.64 (m, 6H). ^{13}C NMR (126 MHz, $CDCl_3$, 25 °C, Figure S5) δ (ppm) 191.8, 150.1 (dd, J_{CF} = 250.4, 13.0 Hz), 147.4 (dd, J_{CF} = 250.4, 13.0 Hz), 144.8, 141.9, 134.7, 133.5 (d, J_{CF} = 2.6 Hz), 130.0 (d, J_{CF} = 12.8 Hz), 128.7 (d, J_{CF} = 13.5 Hz), 127.4, 126.8 (d, J_{CF} = 2.4 Hz), 123.2 (dd, J_{CF} = 7.0, 4.4 Hz), 116.7 (d, J_{CF} = 17.2 Hz). ^{19}F NMR (470 MHz, $CDCl_3$, 25 °C, Figure S6): δ (ppm) –135.8, –137.7. APCI-TOF mass: calcd. for $C_{45}H_{24}F_6O_3$ $[M]^+$: m/z = 726.1624; found: 726.1721.

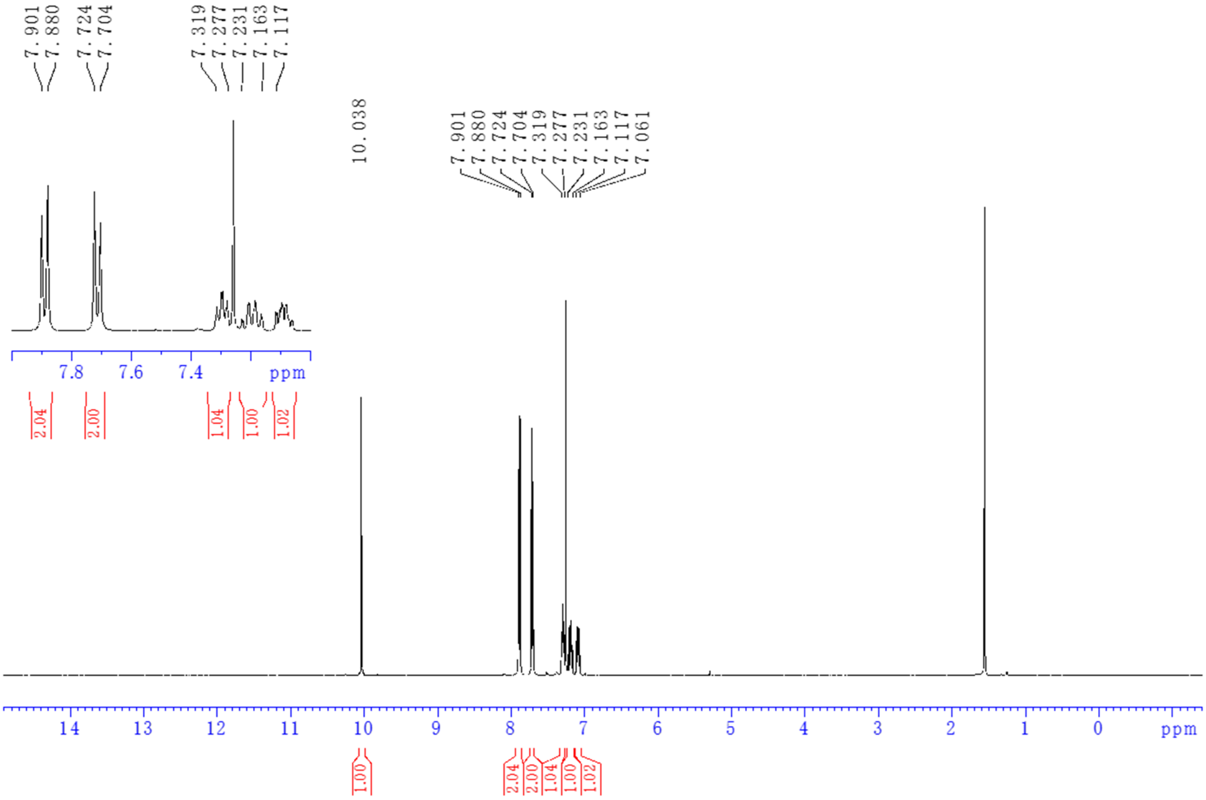

Figure S1. ^{1}H NMR (400 MHz) spectrum of **2** in $CDCl_3$ at 25 °C.

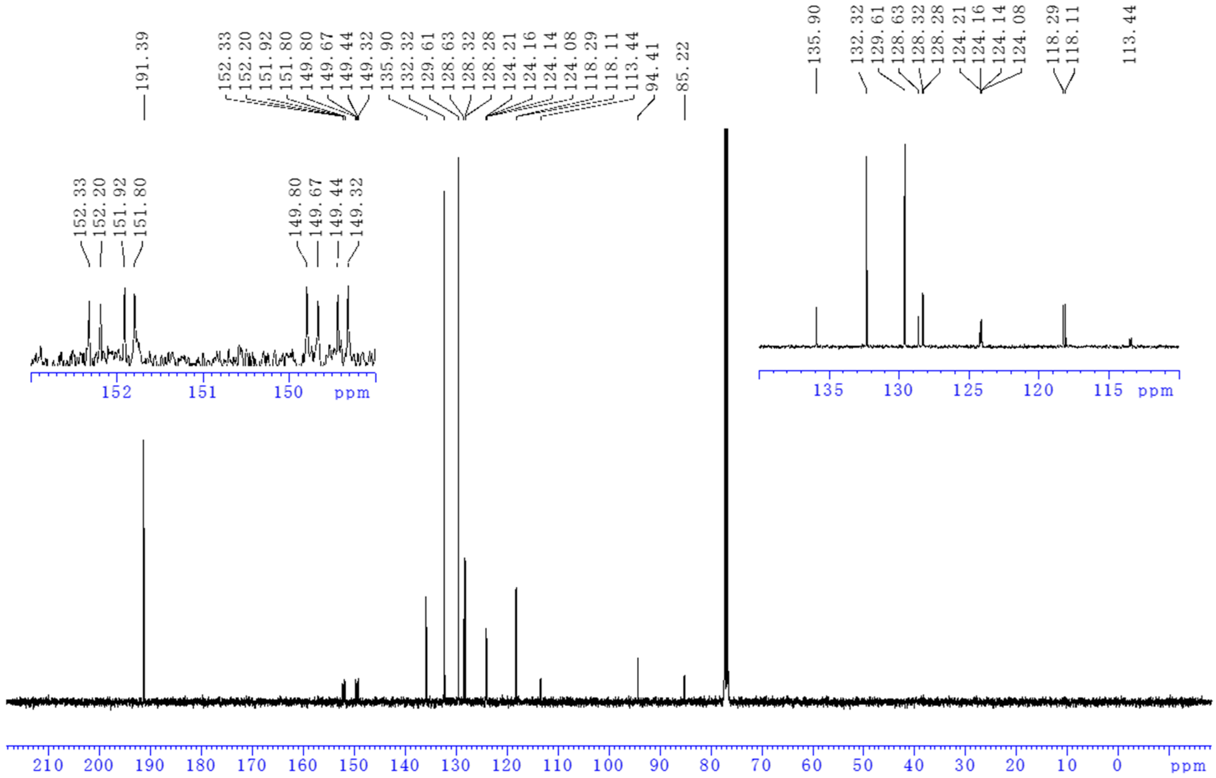

Figure S2. ^{13}C NMR (100 MHz) spectrum of **2** in $CDCl_3$ at 25 °C.

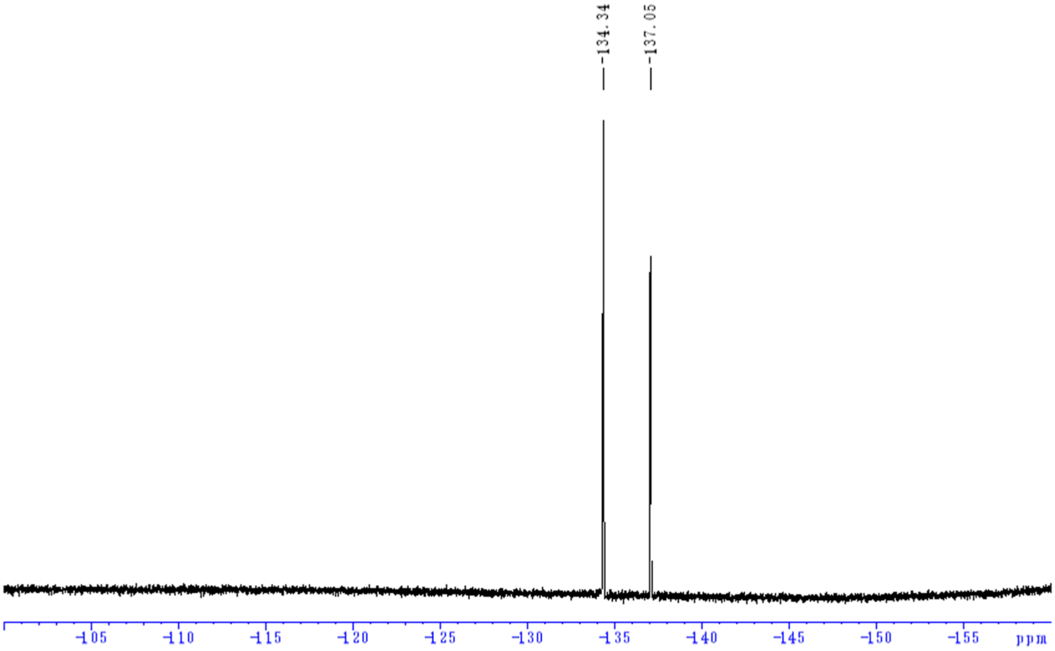

Figure S3. ^{19}F NMR (376 MHz) spectrum of **2** in $CDCl_3$ at 25 °C.

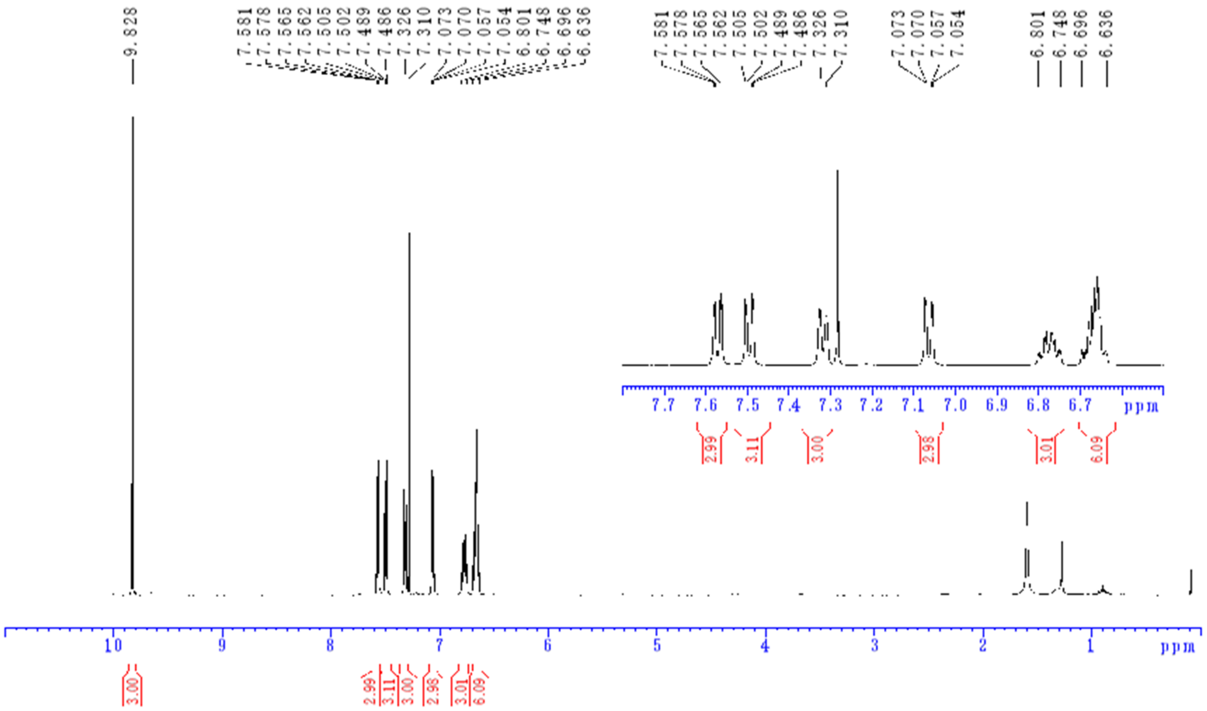

Figure S4. ^{1}H NMR (500 MHz) spectrum of **HABF** in $CDCl_3$ at 25 °C.

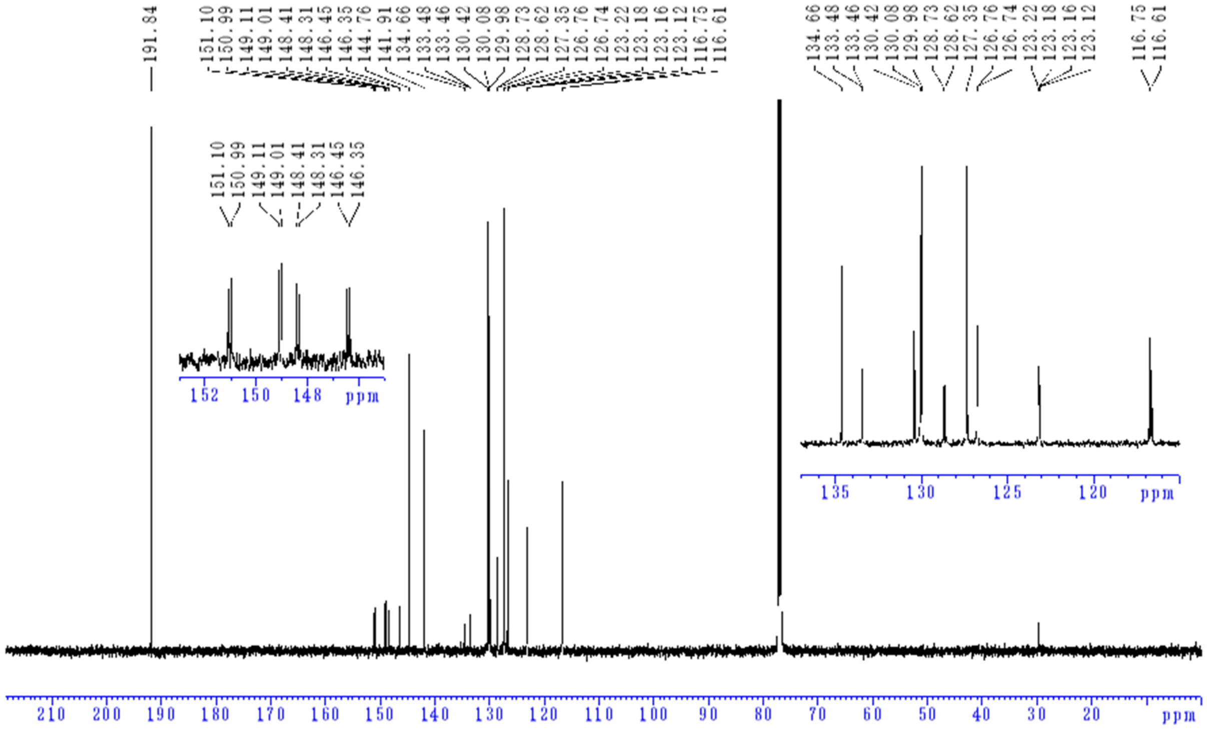

Figure S5. ^{13}C NMR (126 MHz) spectrum of **HABF** in $CDCl_3$ at 25 °C.

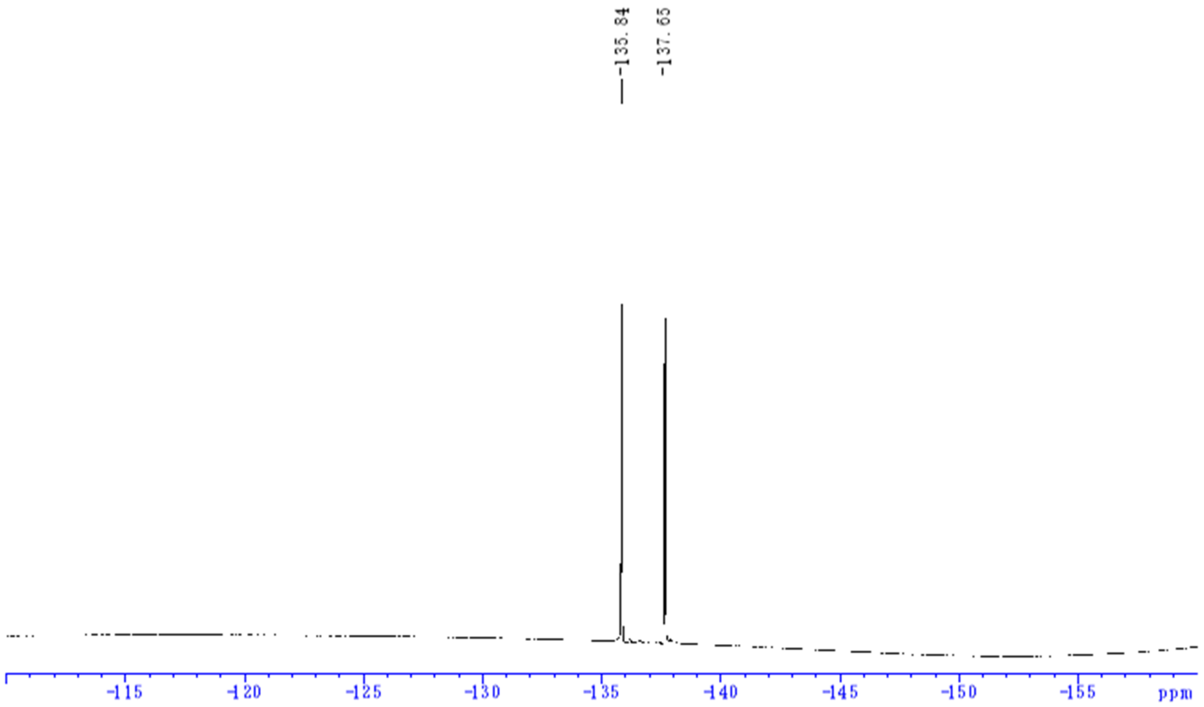

Figure S6. ^{19}F NMR (470 MHz) spectrum of **HABF** in $CDCl_3$ at 25 °C.

S1.3 Synthesis of **TK-COF-P** and **-M**

The COFs were grown according to the procedure described in Figure S7, the detailed conditions of which are summarized in Table S1. The volumetric ratios of 4:1 and 1:1 for 1,4-dioxane:mesitylene were used to grow **TK-COF-P** and **-M**, respectively. We used pre-cleaned screw-cap glass vials (capacity: 2 mL) in these procedures.

As shown in Table S1, we used three conditions (I to III) for **TK-COF-P** and two conditions (IV and V) for **TK-COF-M**. The major difference between them lies in the amount of catalyst AcOH; the smaller (larger) amount of AcOH resulted in lower (higher) yield and less (more) dense population of the COFs, as shown in Figure 1b (<i> *vs.* <ii>) in the main text. The growth conditions of the samples used for each figure are summarized in Table S2.

As shown in Figure S7, first we put **HABF** (1 equiv.) in a 2-mL screw-cap glass vial (Step 1). Then, a mixed solvent (75 μL) of 1,4-dioxane and mesitylene (either 4:1 or 1:1, Table S1) was added to it (Step 2). Separately, we prepared a mixture of aniline (10.8−13.5 equiv.), acetic acid (75−168 equiv.), and an ionic liquid, $[N_{8881}][NTf_2]$ (2.4−3.0 equiv), that was used as a crystal growth enhancer (ref. S1, the mechanism discussed therein). This mixture was ultrasonically homogenized for 5 min, filtered with a PTFE filter (pore size: 0.2 μm, *Merck-LG SLLGX13NL*), and then added to the vial containing the **HABF** solution (Step 3). Subsequently, a 1,4-dioxane solution containing 0.75 equiv. of **TAM** (50 μL), which had been ultrasonically homogenized for 5 min and passed through a PTFE filter, was added to the vial (Step 4). Finally, this vial was stored in a temperature-controlled Peltier incubator (22 or 8 °C) to grow COFs.

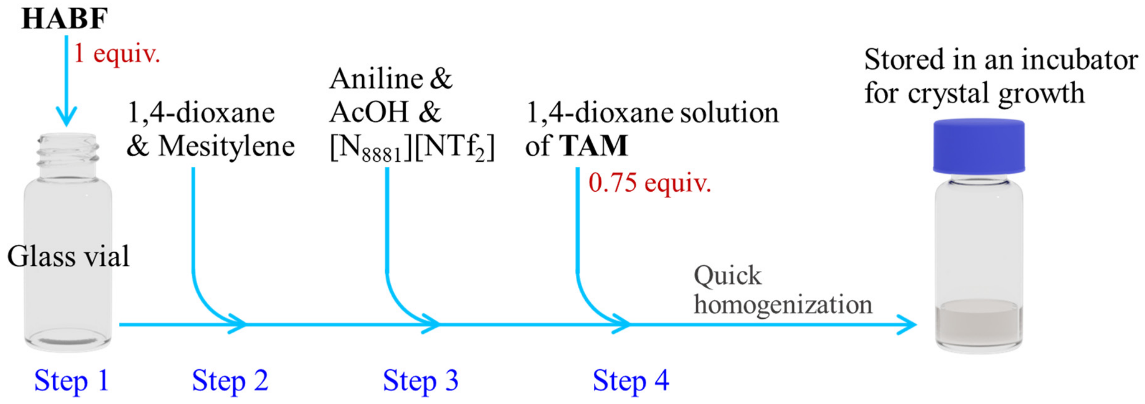

Figure S7. Procedure for the preparation of a solution to grow COFs. See also Table S1.

Table S1. Summary of sample preparation conditions used in the procedure of Figure S7.

Condition No.	Ratio of 1,4-dioxane to mesitylene	Amount of **HABF** added (1 equiv.)	Volumes of 1,4-dioxane and mesitylene added	Volumes of aniline, AcOH, and [N_{8881}][NTf_2] added	Volume of **TAM** solution (16.5 mM in 1,4-dioxane) added (0.75 equiv.)
		Step 1	Step 2	Step 3	Step 4
I	4:1 (v:v) for **TK-COF-P**	1.4 μmol 1.0 mg	50 and 25 μL, respectively	1.7 (13.5), 5.9 (75–84), and 2.3 μL (3.0 equiv.)	50 μL
II				1.7 (13.5), 13.2 (168), and 2.3 μL (3.0 equiv.)	
III		1.75 μmol 1.25 mg		1.7 (10.8), 13.2 (134), and 2.3 μL (2.4 equiv.)	
IV	1:1 (v:v) for **TK-COF-M**	1.4 μmol 1.0 mg	12.5 and 62.5 μL, respectively	1.7 (13.5), 6.6 (84), and 2.3 μL (3.0 equiv.)	
V				1.7 (13.5), 13.2 (168), and 2.3 μL (3.0 equiv.)	

Table S2. Summary of samples used in this report.

Figure #	Purpose	Type of COF	Condition	Temperature	Growth time	Sample state
1b <i>	Optical microscopy	**TK-COF-P**	I	22 °C	30 days	In the crystal growth solution
		**TK-COF-M**	IV	22 °C	20 days	
1b <ii>	Optical microscopy	**TK-COF-P**	II	22 °C	11 days	
		**TK-COF-M**	V	22 °C	6 days	
1b <iii>	SEM	**TK-COF-P**	II	8 °C	14 days	Dried, in vacuum
		**TK-COF-M**	V	22 °C	6 days	

1b <iv>	SEM	**TK-COF-P**	II	8 °C	14 days	
		**TK-COF-M**	V	22 °C	6 days	
1c	PXRD (Cu Kα)	**TK-COF-P**	II	22 °C	11 days	In acetonitrile
		**TK-COF-M**	V	22 °C	7 days	
1d	FT-IR	**TK-COF-P**	II	22 °C	13 days	Dried, in vacuum
		**TK-COF-M**	V	22 °C	7 days	
1e	Solid-state ^{13}C CP/MAS NMR	**TK-COF-P**	II	8 °C	6−14 days	Dried
		**TK-COF-M**	V	22 °C	20 days	
2a	PXRD (synchrotron)	**TK-COF-P**	III	22 °C	11 days	In acetonitrile
2b		**TK-COF-M**	IV	22 °C	14 days	
3b	TEM	**TK-COF-M**	V	22 °C	5 days	Dried, in vacuum
3c	N_2 adsorption	**TK-COF-P**	II	22 °C	14−27 days	Dried
		**TK-COF-M**	V	22 °C	20 days	
3d	TGA	**TK-COF-P**	II	8 °C	7 days	Dried, under N_2 flow
		**TK-COF-M**	V	22 °C	7 days	
3e	Dielectric response	**TK-COF-M**	V	22 °C	2 days	Dried, in vacuum
3g	Temperature dependence of $T_{1\rho}^{-1}$	**TK-COF-M**	V	22 °C	7 days	Dried, under N_2 flow
3h	Comparison of the $T_{1\rho}$ values	**TK-COF-P**	I	8 °C	14−21 days	
		**TK-COF-M**	V	22 °C	7 days	
S10	SEM	**TK-COF-P**	II	8 °C	14 days	Dried, in vacuum
S11		**TK-COF-M**	V	22 °C	6 days	
S12		**TK-COF-M**	IV	22 °C	20 days	
S16	PXRD (Cu Kα)	**TK-COF-M**	V	22 °C	7 days	In acetonitrile
		**TK-COF-M**	V	22 °C	20 days	Dried
S18	Dielectric response	**TK-COF-P**	II	8 °C	3 days	Dried, in vacuum
S19	PXRD (Cu Kα)	**TK-COF-P**	II	8 °C	7 days	Dried, in Ar
		**TK-COF-M**	V	22 °C	8 days	
S20	PXRD (Cu Kα)	**TK-COF-P**	II	8 °C	7 days	Dried, in Ar
		**TK-COF-M**	V	22 °C	8 days	
S21	PXRD (synchrotron)	**TK-COF-P**	III	22 °C	11 days	In acetonitrile
S22		**TK-COF-M**	V	22 °C	14 days	
S34	Optical microscopy	Conversion of **TK-COF-P** to **-M**	II	22 °C	7 days, 15 weeks	In the crystal growth solution
S42	SEM	**TK-COF-P**	III	22 °C	11 days	Dried, in vacuum

S1.4 Elemental analyses (EA)

We used a J-Science Micro Corder JM11 system to conduct EA. Antipyrine and tungsten(VI) oxide were used as a calibration reference and an oxidizer, respectively. To correct the amount of baseline H, which we have found to arise mostly from the oxidizer, the detected amount of H from the EA of tungsten(VI) oxide only was subtracted. The results are summarized in Table S3, showing satisfactory agreement with the theoretical composition.

Table S3. Results of EA.

Element	**Measured** (wt%)		**Calculated** ($C_{85}H_{48}N_4F_8$, wt%)
	Condition II (for **-P**)	Condition V (for **-M**)	
C	78.7	78.73	79.92
H	3.65	3.88	3.79
N	4.52	4.59	4.39
F†	13.13	12.8	11.90

†: Calculated from 100 – (C + H + N)%.

Section S2. Methods of sample characterization

S2.1 Washing and drying procedures before characterization

First, a growth solution containing COF was transferred to an Eppendorf tube (capacity: 1.5 mL), which underwent centrifugation at 60−90 g for 5 min. The COF crystals were then sequentially washed 4−5 times with 1,4-dioxane, 3 times with acetone, and 3 times with toluene using the same Eppendorf tube. For each wash, the supernatant after the centrifugation was replaced by fresh solvent (*ca*. 1 mL), after which the crystals were dispersed again into the freshly added solvent by gentle hand shaking. Finally, the COF was collected on a PTFE filter and dried under dynamic vacuum at 80 °C for 12 h with a flow of 50 sccm dry N_2 gas. The dried COF samples were stored in a glovebox equipped with a circulation gas purifier until characterization.

S2.2 Optical microscopy

Optical images were acquired using an Olympus BX53 microscope equipped with a CMOS camera. Color-sensitive polarized optical images were obtained using a pair of polarizers set in the crossed-Nicols configuration and a retardation plate of 530 nm.

S2.3 Scanning electron microscopy (SEM)

SEM images were obtained using a JEOL JSM-7500F scanning electron microscope (accelerating voltage: 10 kV; working distance: 8 mm) on samples lightly coated with osmium to avoid buildup of charge on the sample. SEM images in the panels a, b, d, and e of Figure S15 were obtained from a JEOL JSM-IT210 scanning electron microscope (accelerating voltage: 10 kV; working distance: 10 mm) on samples lightly coated with gold to avoid buildup of charge.

S2.4 Powder X-ray diffraction (PXRD) measurements

PXRD data in Figures 1c in the main text and Figures S13 and S16 were collected using a Rigaku SmartLab diffractometer with Ni-filtered Cu Kα (λ = 1.54184 Å) radiation at 2000 W (40 kV, 50 mA) power with a scan speed of 0.2° min^{-1}. In these measurements, COF crystals suspended in acetonitrile were loaded in a borosilicate glass capillary tube (diameter: 0.5 mm), which was rotated at 120 rpm during the measurement.

PXRD data in Figures 2a and 2c in the main text and Figures S21 and S22 were collected with synchrotron radiation using RIKEN Materials Science Beamline BL44B2 of SPring-8 (λ = 0.56999 and 0.69995 Å for **TK-COF-P** and **-M**, respectively). These data were used for structural determination (see Sections S4.3 and S4.4). For samples used in the synchrotron PXRD, COF crystals suspended in acetonitrile were sealed in a capillary tube (material: Lindemann glass, diameter: 0.5 mm).

The temperature-dependent PXRD data in Figure S19 over 25–285 °C and the room-temperature PXRD data in Figure S20 were collected using a Rigaku NANOPIX X-ray diffractometer (Cu Kα, λ = 1.54184 Å) equipped with a HyPix-6000 detector. For these measurements, the COF samples were dried using the procedure described in Section S2.1. Each sample was then sealed into a borosilicate glass capillary (diameter: 1.5 mm) using UV-curable resin in an argon-filled glovebox.

S2.5 Fourier-transform infrared (FT-IR) spectroscopy

FT-IR spectroscopy was conducted using a JASCO FT/IR-6100 spectrometer equipped with a single-reflection attenuated total reflectance unit (prism material: germanium). During the

measurements, both the sample and interferometer sections in the spectrometer were evacuated to eliminate the potential interference of atmospheric gases such as CO_2 and H_2O. Usually, the signal was accumulated 128 times to enhance the signal-to-noise ratio. The results are shown in Figure 1d in the main text.

S2.6 ^{13}C solid-state nuclear magnetic resonance (ss-NMR) spectroscopy

Solid-state ^{13}C cross polarization/magic angle spinning (CP/MAS) NMR measurements were performed using a JEOL JNM-ECZ500R (500 MHz) spectrometer equipped with a 3.2-mm HXMAS probe. The spectra were acquired with a 2-ms ramped-amplitude CP at a spinning frequency of 20 kHz. The ^{13}C chemical shifts were referenced to the CH resonance of adamantane at 29.5 ppm (relative to TMS at 0 ppm). The results are shown in Figure 1e in the main text.

S2.7 High-resolution transmission electron microscopy (HR-TEM)

HR-TEM observations were conducted using a JEOL R005 spherical-aberration-corrected transmission electron microscope at an acceleration voltage of 80 kV. **TK-COF-M** samples were washed using the procedure described in Section S2.1. Then, the toluene-replaced samples were drop-casted onto an ultrathin carbon-coated TEM grid (ALLIANCE Biosystems 3150C; carbon thickness: 5 nm, grid material: copper, mesh density: 300), followed by the drying procedure described in Section S2.1.

S2.8 Nitrogen adsorption isotherm measurements

Nitrogen (N_2) adsorption–desorption isotherms were acquired at 77 K using a Micromeritics 3Flex surface area and pore analyzer using ultra-high-purity N_2 (> 99.9995% purity). COF crystals to be measured were dried following the procedures described in Section S2.1 and then degassed before measurements according to the following procedure. First, COF crystals were loaded in the sample glass tube and degassed at 80 °C for 3 h under dynamic vacuum using our home-built vacuum equipment. Then, the sample glass tube was set to the measurement port of the 3Flex and further degassed using the turbomolecular pump of the 3Flex and an equipped mantle heater at 80 °C for 8 h. The results are shown in Figure 3c in the main text.

S2.9 Thermogravimetric analysis (TGA)

TGA measurements were conducted from room temperature to 800 °C using a thermogravimetric differential thermal analyzer (Rigaku, Thermo Plus EVO2) with a flow of 150 mL min^{-1} of nitrogen at a heating rate of 5 °C min^{-1}. Before a measurement, the COF sample was dried following the procedures described in Section S2.1.

S2.10 Temperature-dependent dielectric response measurements

To investigate the temperature dependence of the dielectric response of our COFs, we developed the measurement samples and system. We prepared the samples according to the procedure described graphically in Figure S8, in which we used small rectangular ITO glass substrates (length: 16 mm; width: 7 mm; thickness: 0.7 mm; Figure S8(i)). We added a metal wire (nickel, diameter: 0.15 mm) to the upper area of the ITO side of the plate using a high-melting-point, flux-free solder (CERASOLZER®, melting point = 297 °C; Figure S8(ii)). Then, the COF was grown directly on the surface of the glass plate held in a glass vial (capacity: 3 mL, outer diameter (O.D.):

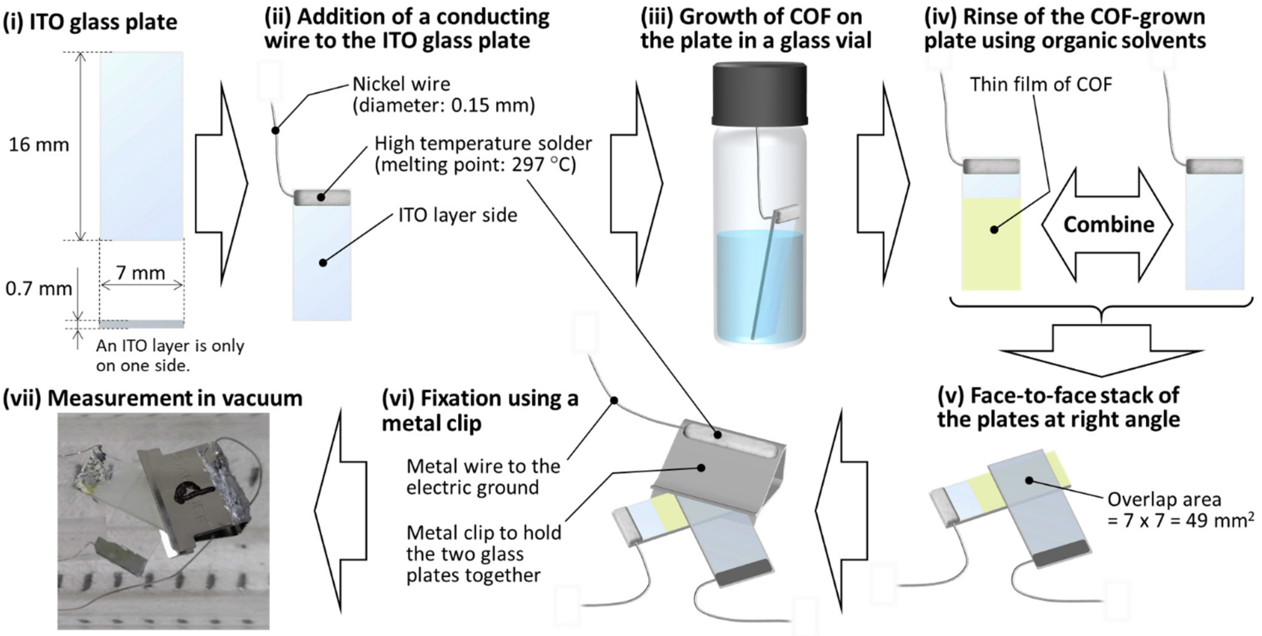

Figure S8. Preparation of the sample for the temperature-dependent dielectric response measurements.

13 mm) for 2 or 3 days (Figure S8(iii)) following the procedure described in Section S1.3, which typically resulted in a COF layer with a thickness of 2 to 8 μm). After the plate with the COF layer was taken out of the glass vial, the plate was washed by submerging it in dioxane (typically for 5 min, to remove the reactants, aniline, and acetic acid), then in acetonitrile (typically for 5 min), and finally in toluene (typically for 10 min). Subsequently, the plate was dried in ambient conditions for a few minutes and combined with a blank ITO glass plate (Figure S8(iv)) as follows. These two plates were stacked at a right angle to each other so that their ITO-plane sides face each other (Figure S8(v)). Then, this stack was fixed using a metal clip to which a metal wire had been added using the same high-melting-point solder (Figure S8(vi)), the entirety of which was then dried in vacuum at 80 °C for 12–24 h. This sample was used for dielectric response measurements in vacuum (Figure S8(vii)) at different temperatures, as described next.

To carry out this measurement, we constructed a home-built experimental setup, shown in Figure S9. The system consisted of an annular electric furnace (power = 500 W), a quartz tube (O.D. = 30 mm, inner diameter (I.D.) = 26 mm), heat-resistant semi-rigid coaxial cables (PASTERNACK, PECX006-BULK; O.D. = 0.086″, impedance = 50 Ω, dielectric insulator: PTFE), a type-T thermocouple (electrically insulated by being encased in a PFA tube, the bottom photograph of Figure S9), a temperature controller for the furnace (CHINO, KP1000), and an oil-free, dry-scroll vacuum pump (EDWARDS, nXDS15i). All soldering for connecting wires and

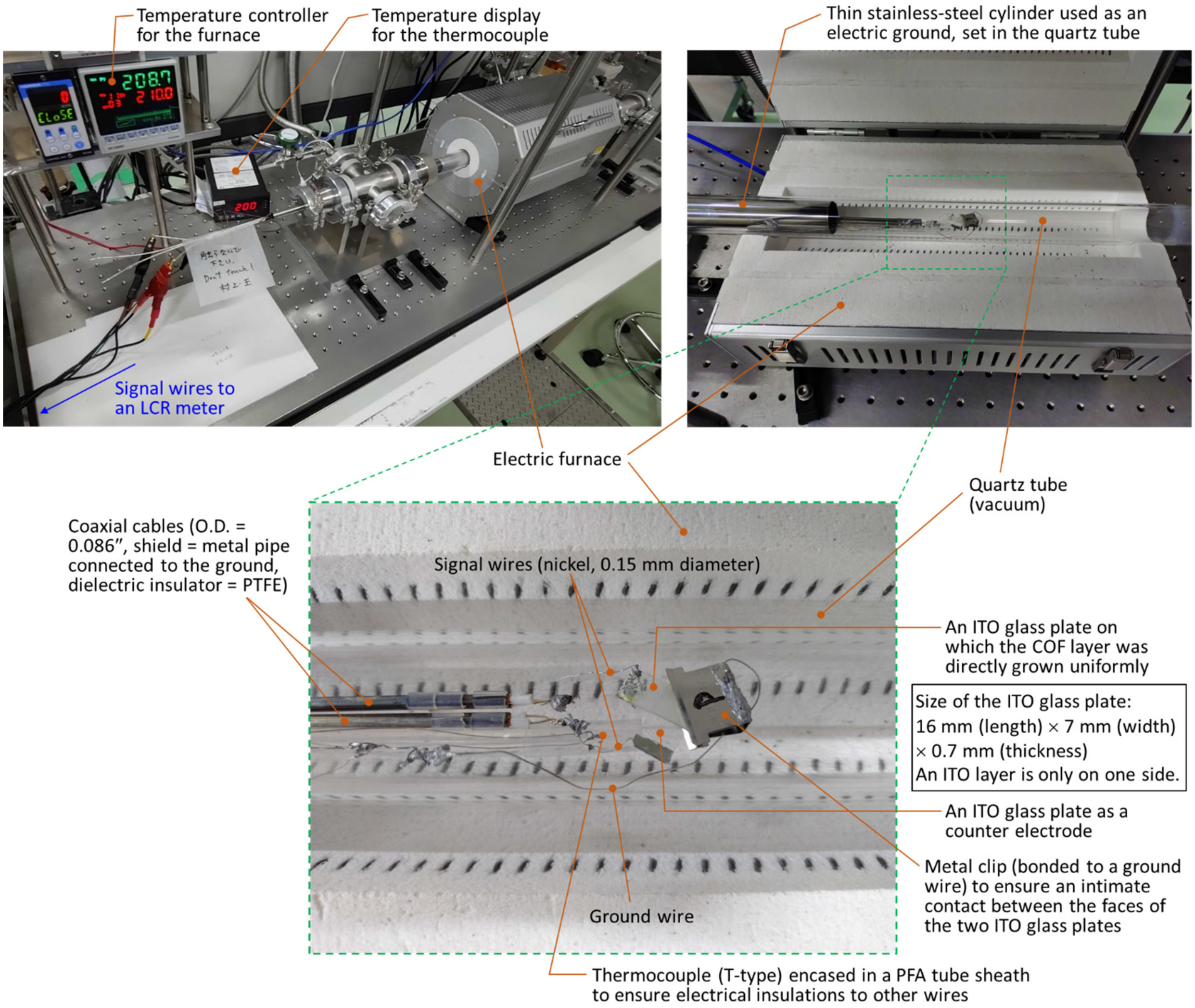

Figure S9. Photographs of our home-built experimental system used for the temperature-dependent dielectric response measurements.

forming thermocouple junctions was done using the high-temperature, flux-free solder (CERASOLZER®, melting point = 297 °C). The temperature of the sample was measured by placing the thermocouple junction near the sample and using a digital display to which the thermocouple was connected (Figure S9; accuracy: ±1.5 °C).

It is noted that the maximum allowable temperature of the sample in this setup was set by us to be 285 °C, because (i) the warranted upper temperature of the coaxial cable for continuous use

was 250 °C and (ii) the melting point of the aforementioned solder was 297 °C. This is the reason why the measurement for the high temperature side had to end at 285 °C in Figures 3e and S18.

We have confirmed that we could obtain reproducible data up to a sample temperature of 285 °C without damaging the sample owing to the oxygen-free, vacuum environment as well as the intrinsic high thermal stabilities of the present COFs (see the TGA results shown in Figure 3d in the main text). The outer shield of the coaxial cables and the metal clip were connected to the common electric ground. By doing this, we achieved very low floating capacitance of the system of only *ca.* 0.2 pF, which ensured reliable measurements because our samples typically had a capacitance of 50–250 pF, *i.e.*, sufficiently larger than the floating capacitance of the experimental system.

All cables were directed to the outside of the quartz tube through our homemade feed-through port. The signal wires of the coaxial cables were connected to an LCR meter (NF Corporation, ZM2376). The real and imaginary parts of the impedance ($Z'$and Z'', respectively) and the phase (θ) were recorded for the frequency range of 2 Hz to 500 kHz with an applied AC voltage amplitude of 1 V. The lower limit of the frequency in the present experimental system was approximately 2 Hz; further lowering of the frequency led to very high sample impedance that may not be reliably measured using our LCR meter. The temperature was varied from room temperature (22–24 °C) to 286 °C.

Finally, the measured Z' and Z'' values were analyzed assuming a parallel-capacitance–resistance model along with the information about the thickness of the COF layer and sampling area (49 mm^2) to obtain the real and imaginary part of the dielectric permittivity (ε' and ε'', respectively). Analytically, ε' and ε'' are given by the following equations S1 and S2[S2]

$$\varepsilon' = \frac{Z''}{\varepsilon_0 \cdot 2\pi f\left(Z'^2 + Z''^2\right)} \cdot \frac{l}{A} \tag{S1}$$

$$\varepsilon'' = \frac{Z'}{\varepsilon_0 \cdot 2\pi f\left(Z'^2 + Z''^2\right)} \cdot \frac{l}{A}, \tag{S2}$$

where Z' and Z'' are the real and imaginary parts of the impedance, respectively; ε_0 is the permittivity of vacuum (= 8.854 × 10^{-12} F/m); f is the frequency; l is the thickness of the sample between planar electrodes; and A (= 49 mm^2) is the sample area.

The results of ε' and ε'' for the **TK-COF-M** are shown in Figure 3e in the main text and those for **TK-COF-P** are shown in Figure S18 below. These results have revealed the most important aspect of the developed COFs—that the response of the dipolar rotors to the external field begins only above 150 °C, demonstrating their potential utility as dipolar memories that can be manipulated by an external field and hold the information at ambient temperature.

The absolute magnitudes of ε' and ε'' obtained for **TK-COF-M** and **TK-COF-P** in Figures 3e and S18 possess ±50% and ±100% uncertainty, respectively, which originates mainly from the uncertainty in the thickness of the COF layer on the ITO glass plate arising from the inaccuracy in the thickness measurement and possible thickness nonuniformity over the sample area. See also the descriptions in Section S3.5.

Section S3. Supporting results of sample characterization

S3.1 Full-size SEM images

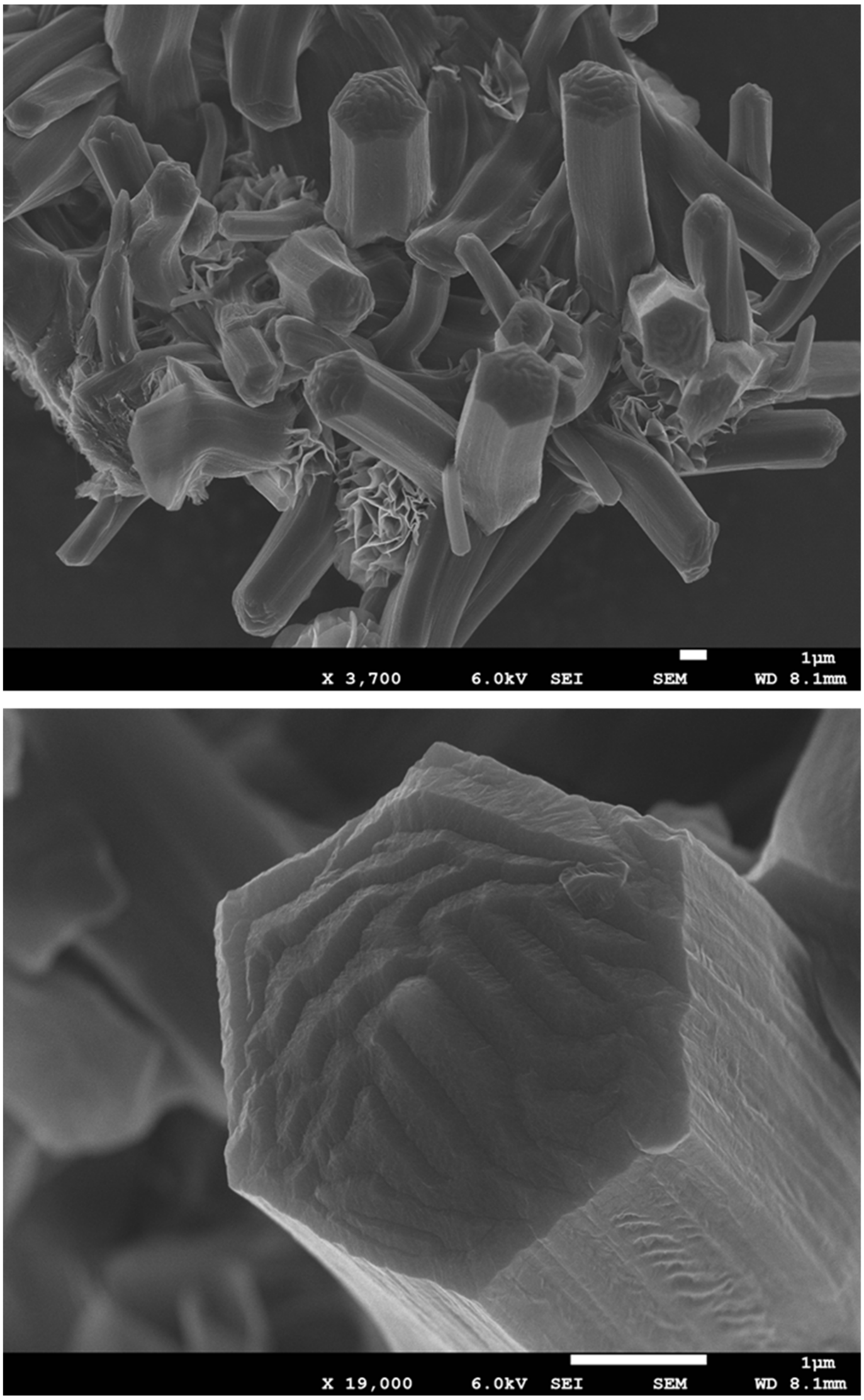

Figure S10. Full-size SEM images of **TK-COF-P** shown as Figures 1b <iii> and <iv> in the main text.

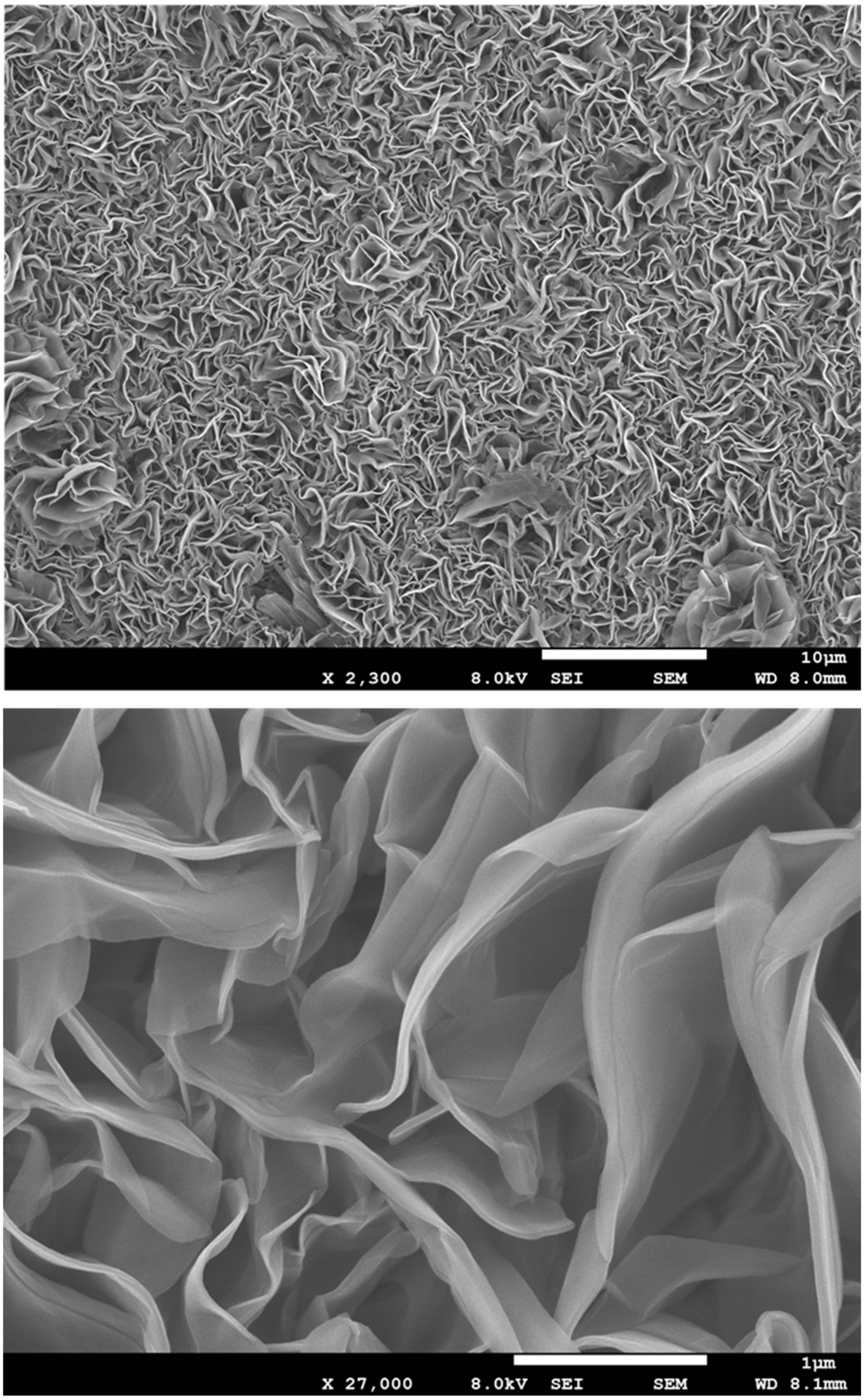

Figure S11. Full-size SEM images of **TK-COF-M** shown as Figures 1b <iii> and <iv> in the main text.

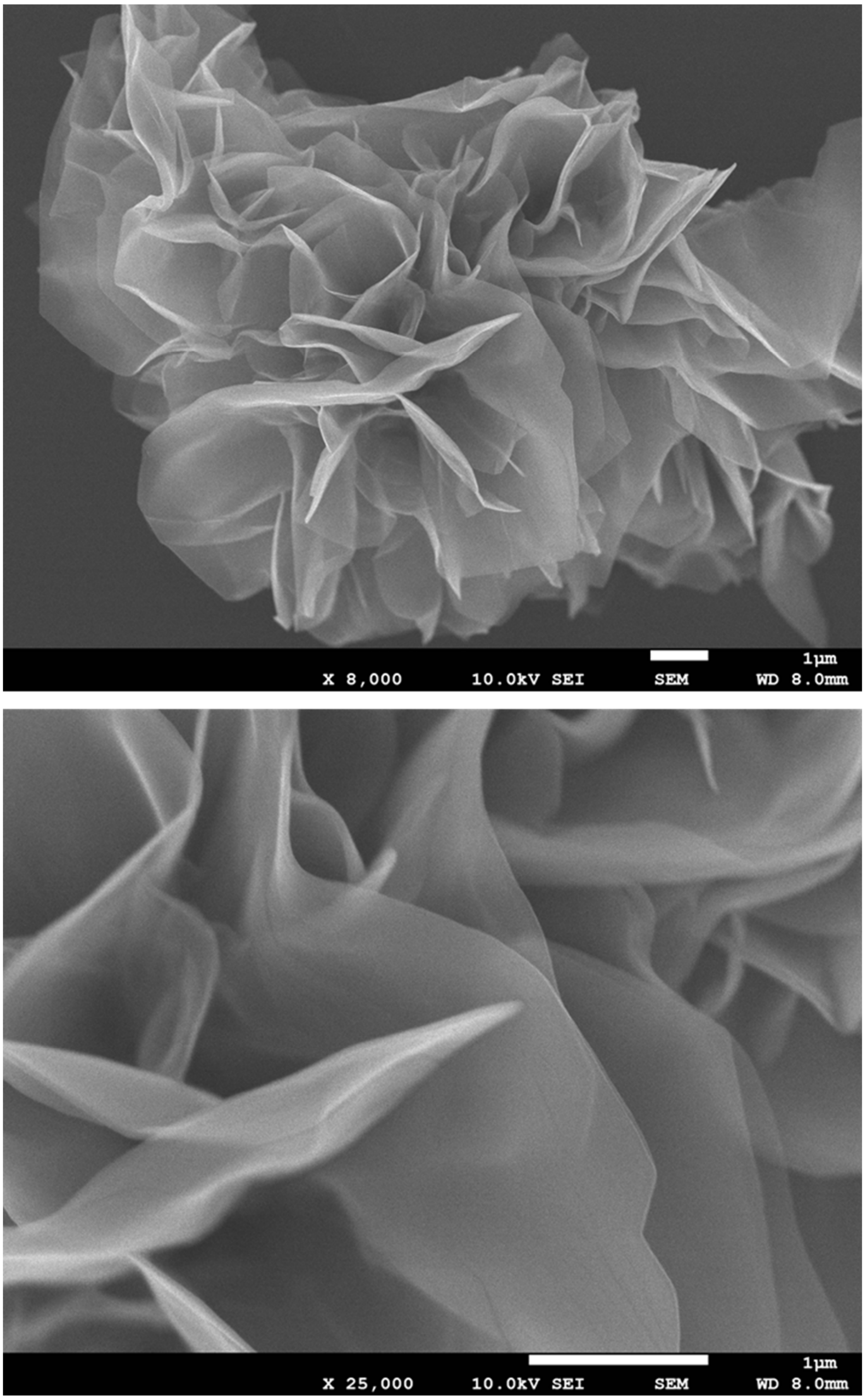

Figure S12. Full-size SEM images of **TK-COF-M** shown in Figures 1b <i> in the main text.

S3.2 Synthesis using building-block molecules in which the F atoms in **HABF** were replaced by H atoms

As reference experiments, we tried to synthesize COFs using an aldehyde building-block **HAB** (Scheme S2) that is similar to **HABF** but does not have F atoms. To prepare those samples, we used the same procedures as those used to grow **TK-COF-P** and **-M** (Figure S7) with four conditions, denoted Control #1 to 4, as summarized in Table S4. They are typical conditions we used to generate **TK-COF-P** and **-M** (see also Table S1). The growth solutions were stored in a Peltier incubator at 22 ± 0.5 °C for 7 days.

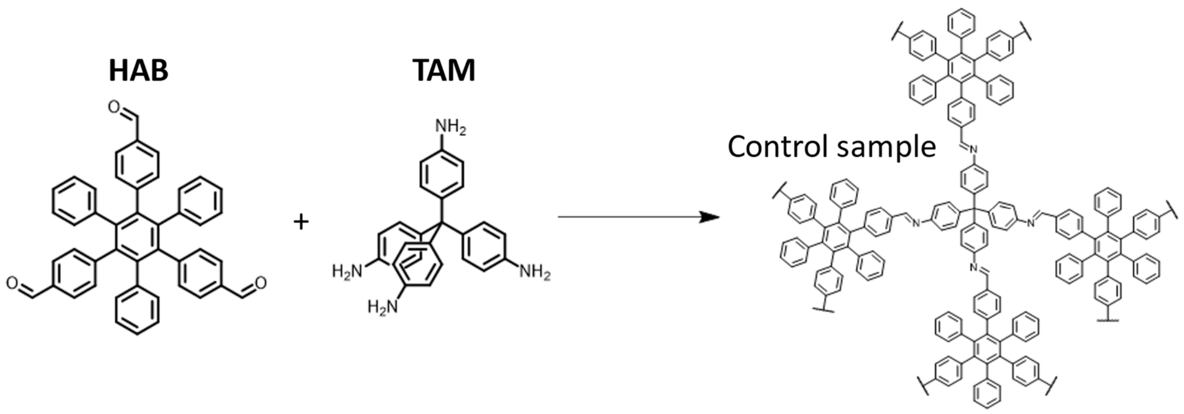

Scheme S2. Scheme to reticulate **HAB** and **TAM** via imine condensation.

Solid precipitates were obtained under all these conditions. Their PXRD patterns had only a few broad and low-intensity peaks in the low 2θ region, indicating significantly lower crystallinity than **TK-COF-P/-M** (Figure S13). The optical microscopy images (Figure S14) and SEM images (Figure S15) revealed that these precipitates did not have distinct crystalline shapes, suggesting that these were close to amorphous solids. Thus, the lack of F atoms in the aldehyde building-block molecule (*i.e.*, the use of **HAB** instead of **HABF**) resulted in solids with substantially low crystallinity. These results indicate that F atoms are essential to generate highly crystalline COFs or **TK-COF-P/-M** in the present study.

Table S4. Conditions to obtain reference solids using **HAB** and **TAM**.

Condition no.	**HAB**	**TAM**	Dioxane:Mesitylene (v:v)	Acetic acid	Aniline	[N_{8881}][NTf_2]
Control #1	1 equiv. (1.02 mg)	0.75 equiv. (0.47 mg)	4:1	84 equiv. (7.9 μL)	13.5 equiv. (2.0 μL)	2.96 equiv. (2.8 μL)
Control #2				168 equiv. (15.8 μL)		
Control #3			1:1	84 equiv. (7.9 μL)		
Control #4				168 equiv. (15.8 μL)		

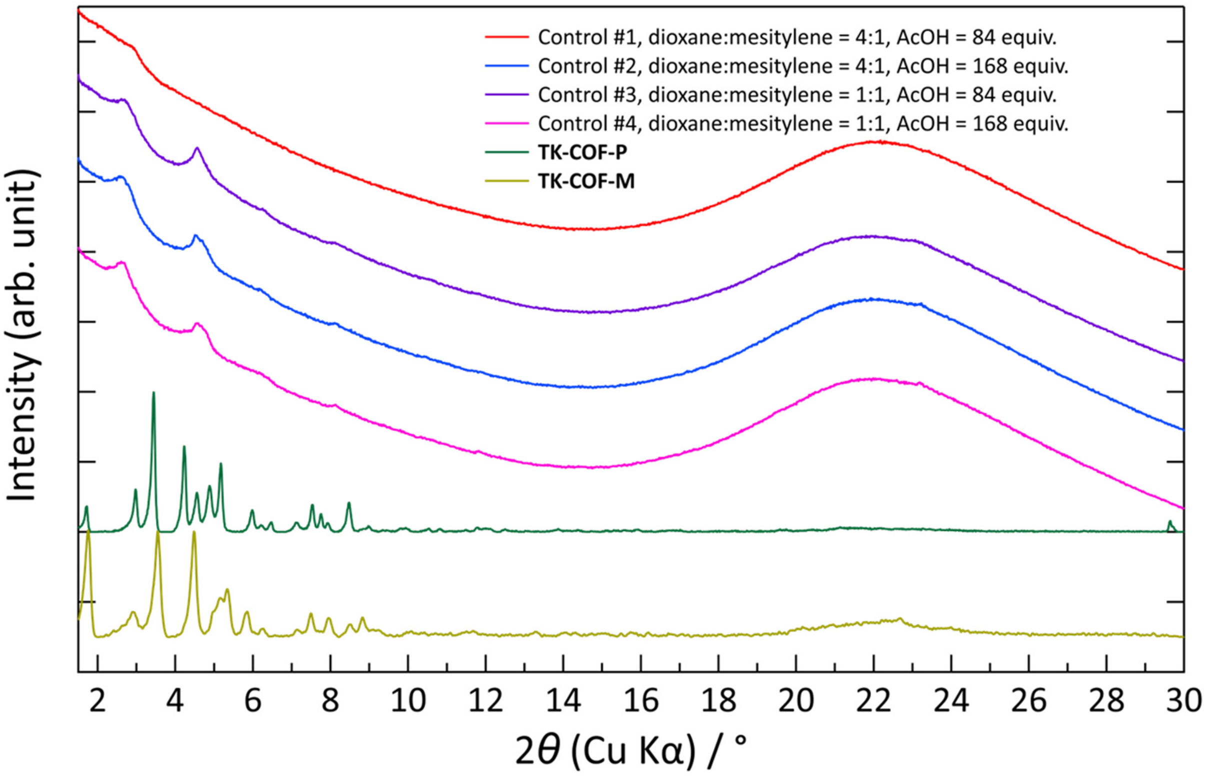

Figure S13. Comparison of the PXRD patterns of **TK-COF-P/-M** with those of the solid precipitates generated from **HAB** and **TAM** using the conditions of Control #1 to 4 (see Table S4).

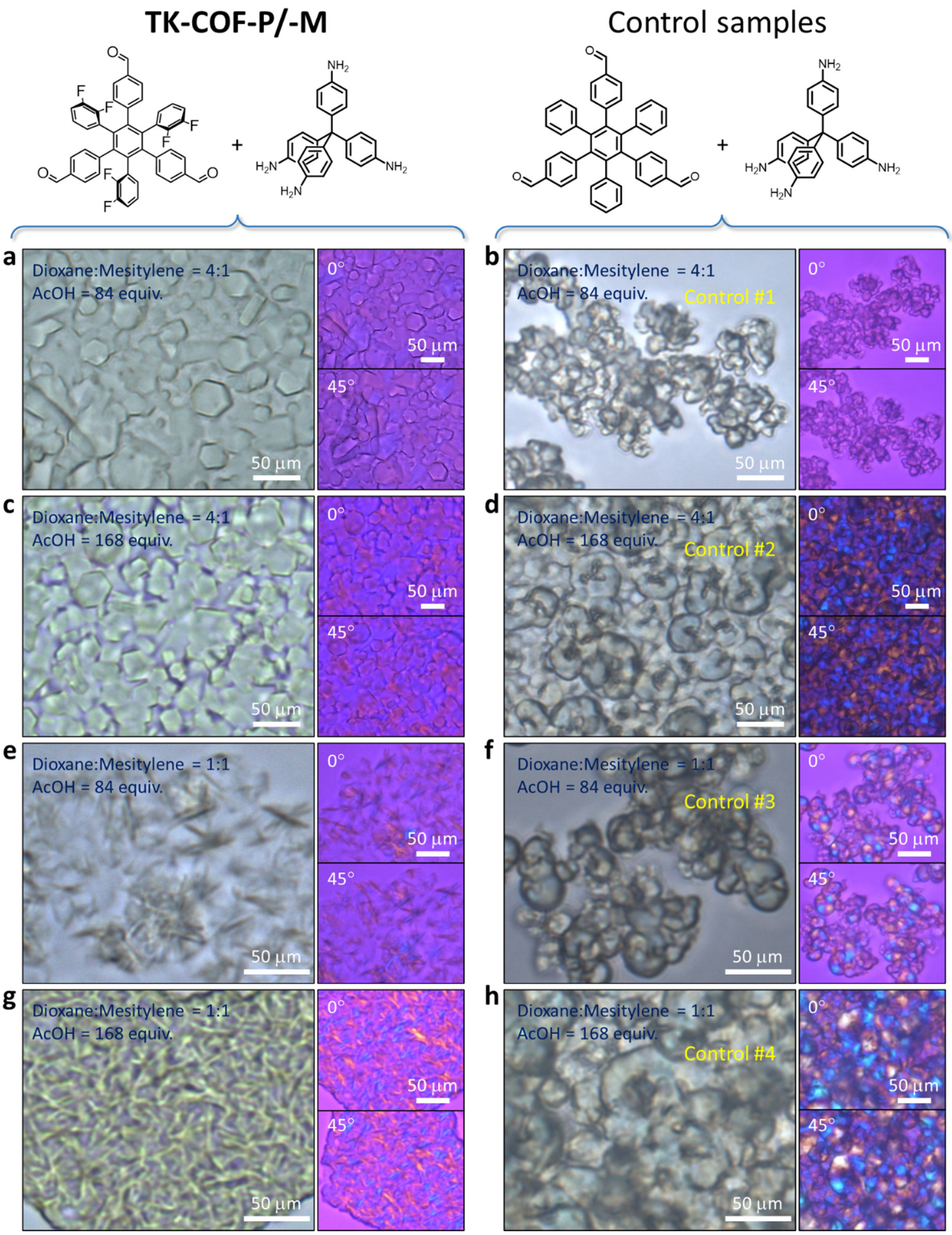

Figure S14. Comparison of the morphologies observed under an optical microscope of (a, c, e, g) **TK-COF-P/-M** and (b, d, f, h) the control samples described in Scheme S2, generated using the conditions of (a, b) Control #1, (c, d) Control #2, (e, f) Control #3, and (g, h) Control #4 summarized in Table S4.

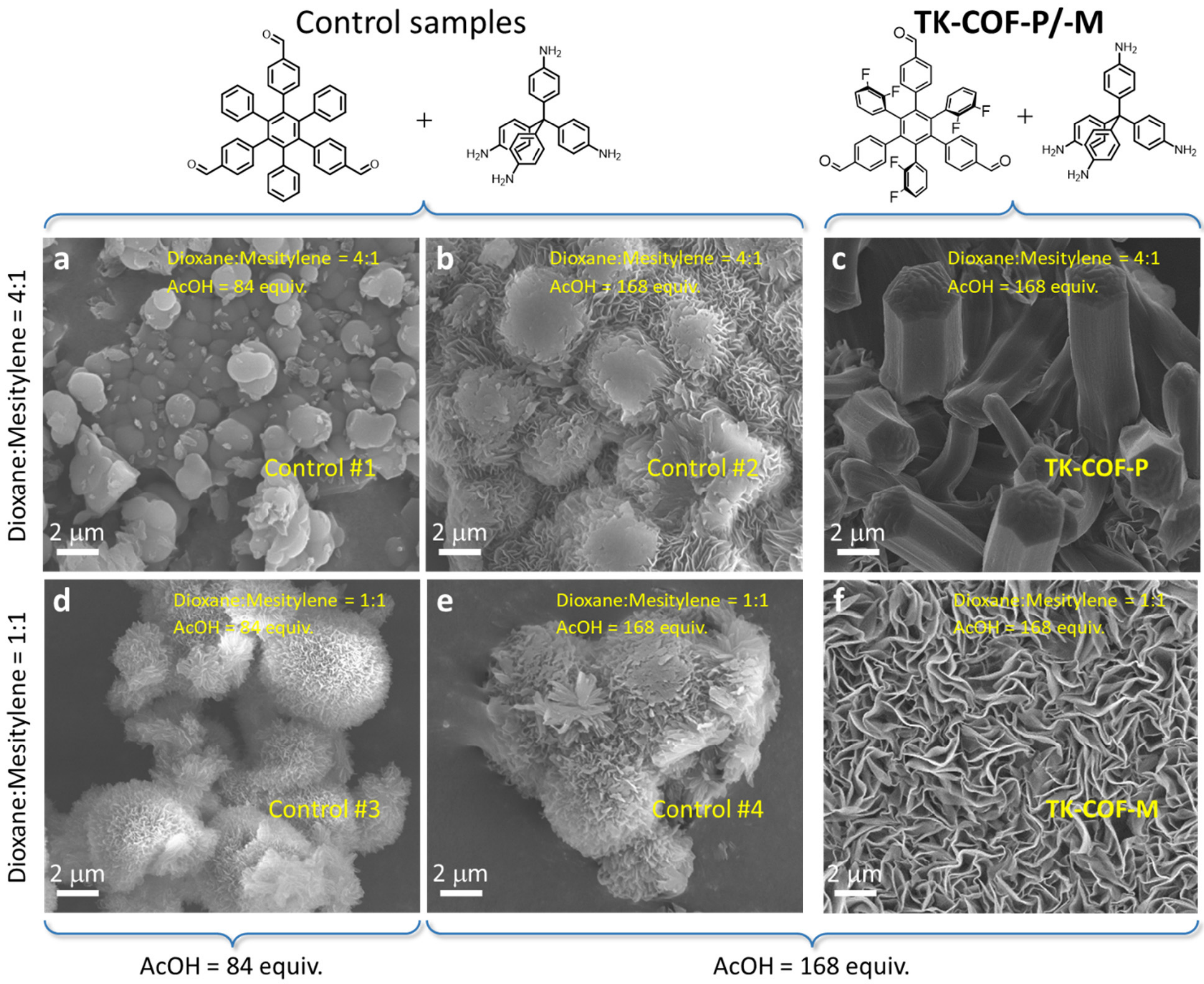

Figure S15. Comparison of the morphologies observed under an SEM of (a, b, d, e) the control samples generated using the conditions of (a) Control #1, (b) Control #2, (d) Control #3, and (e) Control #4 with those of (c) **TK-COF-P** synthesized with Condition II and (f) **TK-COF-M** synthesized with Condition V.

S3.3 PXRD patterns of **TK-COF-M** before and after drying

To investigate the influence of solvent removal, we compared the PXRD patterns of **TK-COF-M** before and after the removal of acetonitrile (Figure S16) by the drying procedure described in Section S2.1. The PXRD pattern for the dried **TK-COF-M** was measured using reflection mode in Bragg-Brentano geometry. In the dried state, **TK-COF-M** exhibited broad peaks with much lower intensity, suggesting a reduction of long-range order due to drying.[S3] However, the results of ^{13}C solid-state NMR, FT-IR, and EA have confirmed the integrity of the skeleton connectivity of **TK-COF-M** after the solvent removal.

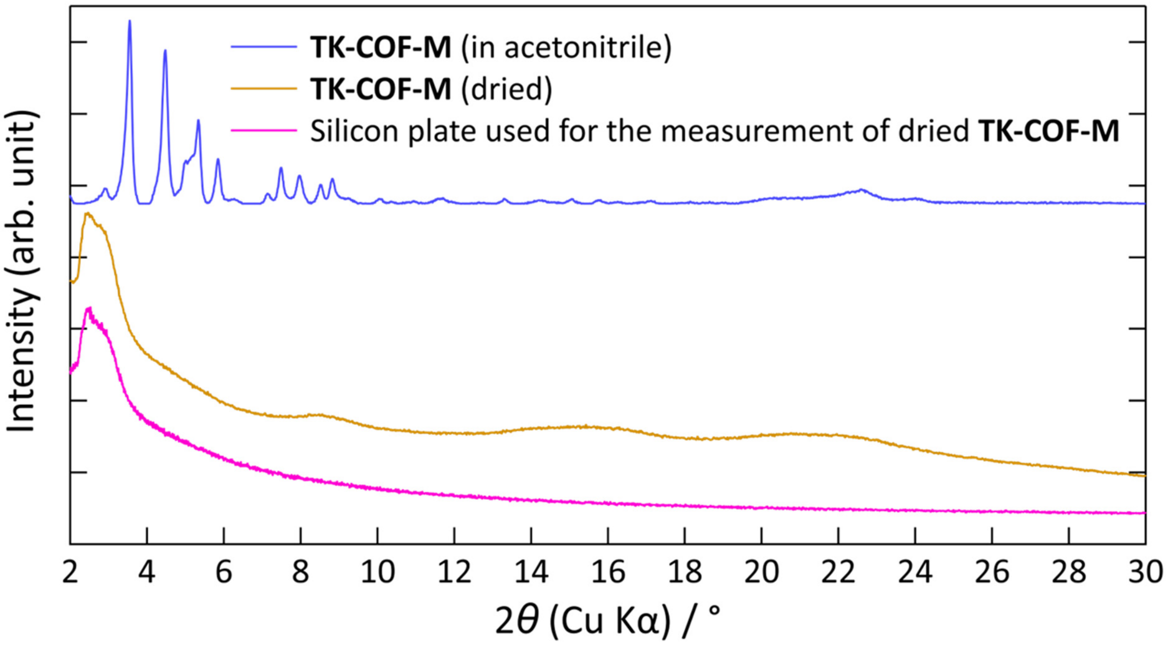

Figure S16. Comparison of the PXRD patterns of **TK-COF-M** before and after the removal of acetonitrile. The PXRD pattern of **TK-COF-M** in acetonitrile (blue) is the same as in Figure 1c in the main text. The PXRD pattern of the blank silicon plate (pink) used to measure **TK-COF-M** in the reflection mode is also shown.

S3.4 BET surface area plots

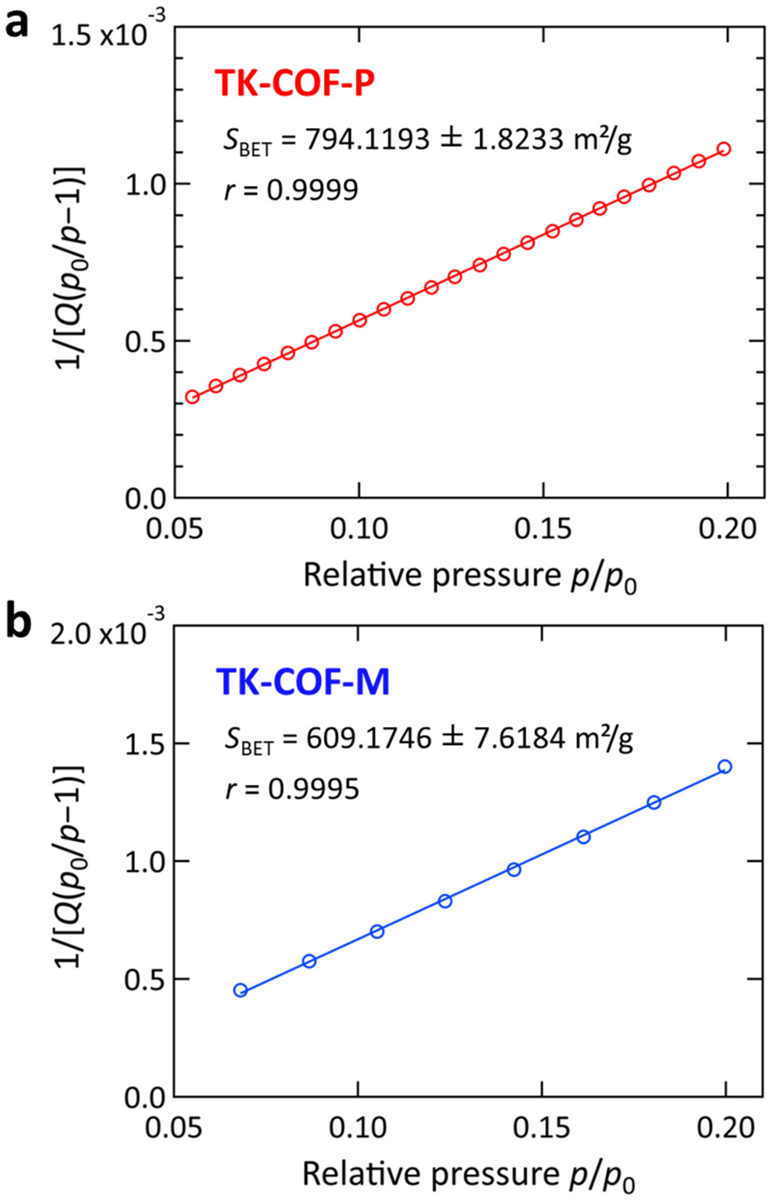

Figure S17. BET surface area plots for (a) **TK-COF-P** and (b) **-M** generated from the N_2 adsorption isotherms in Figure 3c in the main text. The BET surface area (S_{BET}) values are shown in the panels.

S3.5 Temperature-dependent dielectric response of **TK-COF-P**

The temperature dependence of ε' and ε'' of **TK-COF-P** is shown in Figure S18. The experimental and analytical details have been given in Section S2.10. Due to the large uncertainty in the estimated thickness of the COF film formed on the ITO substrate and the fact that the values of ε' and ε'' depend on the sample thickness (see equations S1 and S2 in Section S2.10), the magnitudes of ε' and ε'' shown in Figure S18 are considered to possess an uncertainty of ±100 % or more. (Note: The values of ε' and ε'' shown for **TK-COF-M** in Figure 3e in the main text are estimated to have an uncertainty up to ±50% owing to the uncertainty in the COF film thickness.) Therefore, these results should be taken to reveal an important *quantitative* tendency that the present COFs are dielectrically non-responsive below 150 °C and respond to the external field only above 200 °C, hence evidencing the ability to maintain the rotors' orientation under ambient temperatures.

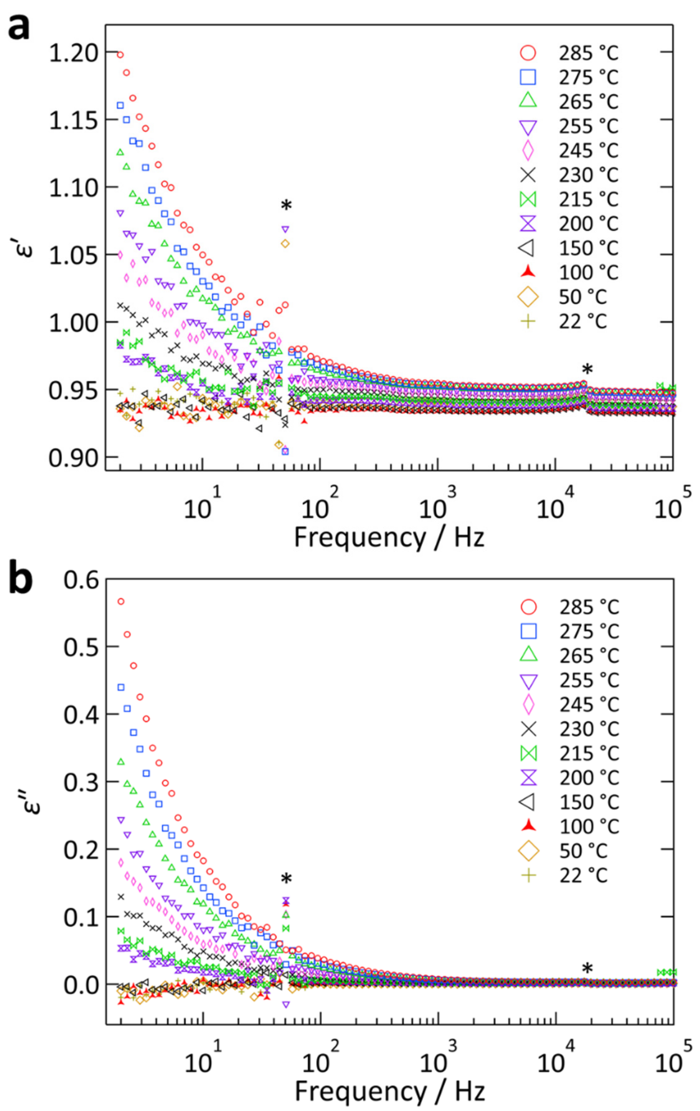

Figure S18. Temperature dependence of (a) the real part, ε', and (b) the imaginary part, ε'', of the dielectric permittivity of **TK-COF-P** formed on an ITO glass plate. See Section S2.10 for the experimental and analytical details. The magnitudes of ε' and ε'' herein are considered to possess an uncertainty of ±100 % or more; see the text above for the reason. The asterisks indicate the noise signals that occurred when the LCR meter changed its operating range.

S3.6 Temperature-dependent PXRD patterns

The temperature-dependent PXRD patterns of **TK-COF-P** and **TK-COF-M** were acquired at 25, 85, 185, and 285 °C. As the results (Figure S19) show, the positions of the peaks, especially those below 10°, did not vary noticeably with the increase of the temperature, indicating that the periodicity of the frameworks exhibited almost no change over the temperature range investigated.

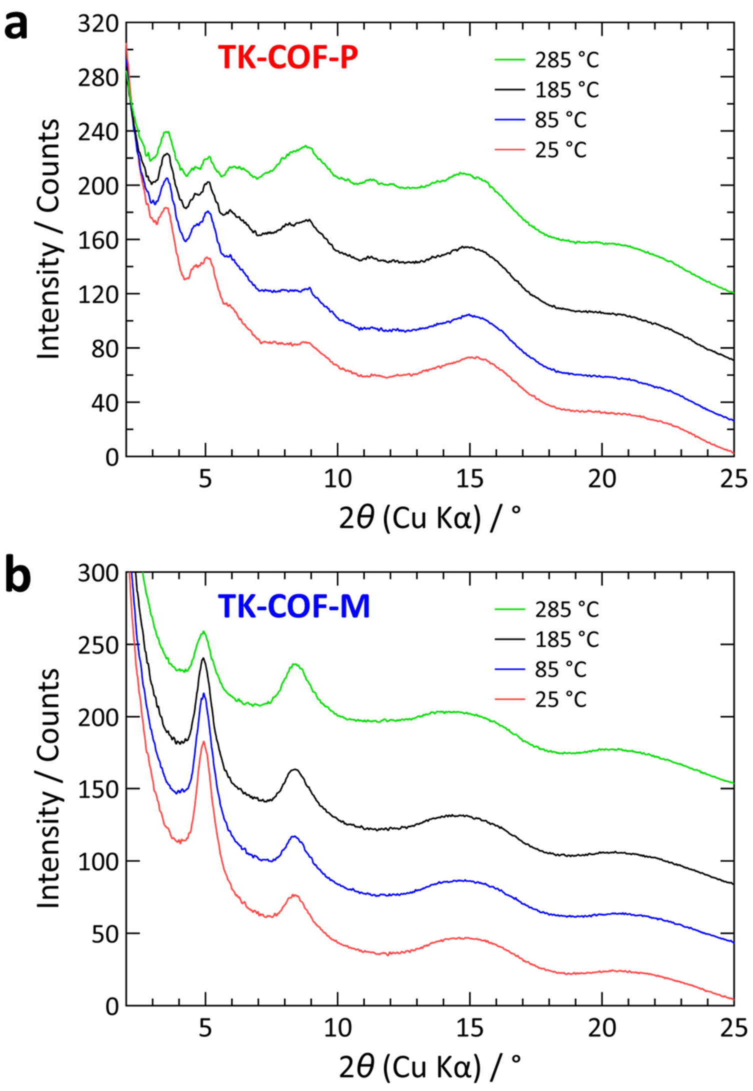

Figure S19. Temperature-dependent PXRD patterns of (a) **TK-COF-P** and (b) **TK-COF-M**.

S3.7 Comparison of PXRD patterns before and after heating to 285 °C

To confirm that the structure was retained after being heated up to 285 °C in the dielectric response measurements (Figures 3e and 3f in the main text, Figure S18), we compared the PXRD patterns of **TK-COF-P** and **-M** before and after heating. First, the samples were dried using the procedure described in Section S2.1. Then, the samples were heated to and maintained at 285 °C for 2 h in the experimental setup we used for the dielectric response measurements (Figure S9).

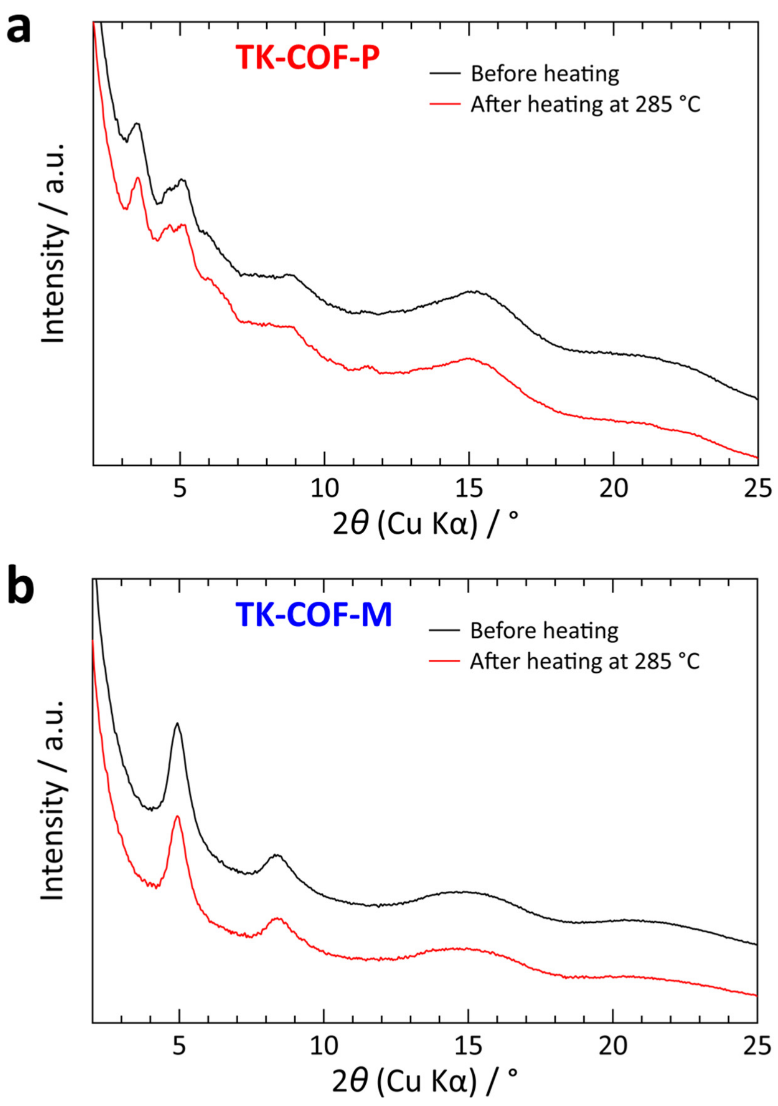

Figure S20. Comparisons of the PXRD patterns of (a) **TK-COF-P** and (b) **TK-COF-M** before and after heating at 285 °C for 2 h in the apparatus used for the dielectric response measurements.

After the samples were cooled to room temperature, the PXRD patterns were acquired and compared with the patterns acquired from the same samples without heating. The results, as shown in Figures S20a and S20b for **TK-COF-P** and **TK-COF-M**, respectively, confirm that the structures of these COFs were retained after heating at 285 °C.

Section S4. Structural determination of TK-COF-P/-M by Rietveld refinement

S4.1 Synchrotron PXRD patterns

For structural determinations of **TK-COF-P** and **-M**, their PXRD patterns were acquired using synchrotron radiation (see Section S2.4 for details). The patterns from **TK-COF-P** (Figure S21) and **TK-COF-M** (Figure S22) were acquired in acetonitrile using the radiation wavelengths of λ = 0.56999 and 0.69995 Å, respectively.

In Figure S23, the PXRD patterns of **TK-COF-P** and **-M** acquired using different X-ray sources (the synchrotron vs. our laboratory Rigaku SmartLab X-ray diffractometer) were compared, showing coincidences in these PXRD patterns. Because the sample batches used for the acquisition of the patterns using the synchrotron and the laboratory X-ray diffractometer were different, this comparison also indicates the good reproducibility of the shape of the PXRD patterns.

Although we tried single-crystal X-ray diffraction measurements, we could only obtain small number of diffraction spots that were insufficient for structural determination, presumably owing to the softness of the present COFs as evidenced by the remarkable shape distortions seen in the dried state (*cf.* SEM images under vacuum, Figure S10). Therefore, we were restricted to the high-resolution PXRD data acquired using the synchrotron radiation to determine the structures.

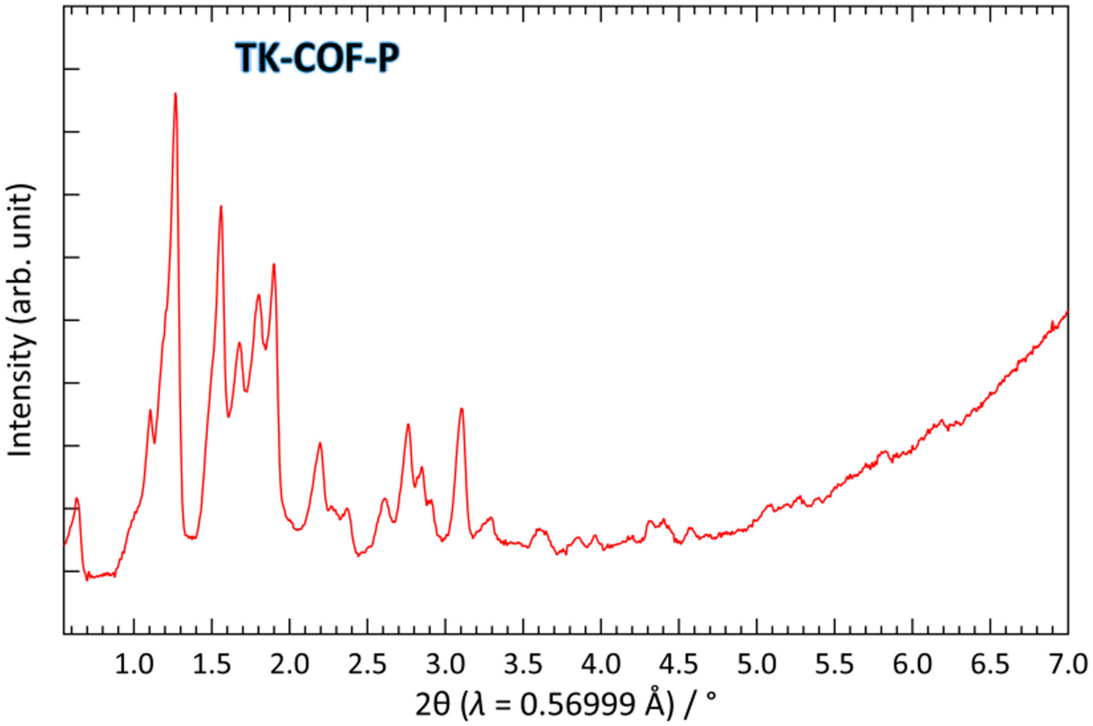

Figure S21. PXRD pattern of **TK-COF-P** in acetonitrile acquired using synchrotron radiation (λ = 0.56999 Å).

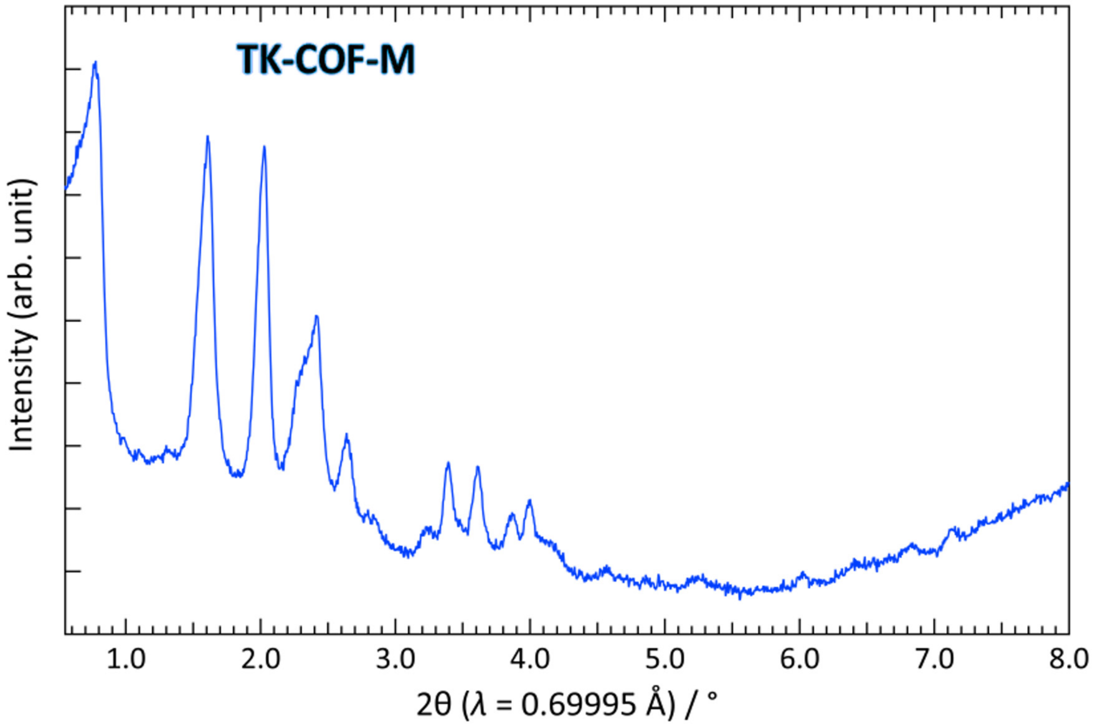

Figure S22. PXRD pattern of **TK-COF-M** in acetonitrile acquired using synchrotron radiation (λ = 0.69995 Å).

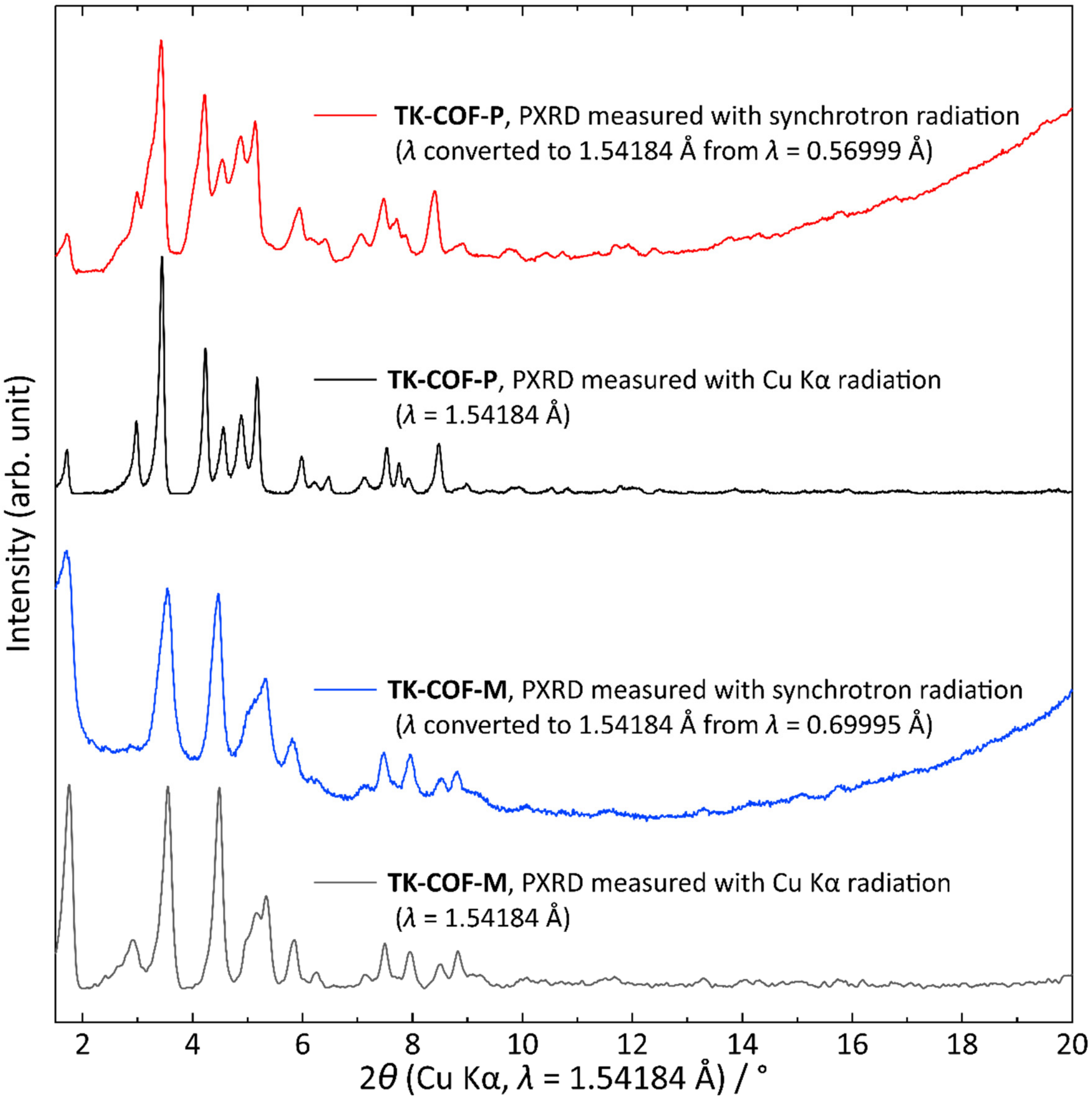

Figure S23. Comparison of the PXRD patterns of **TK-COF-P** and **-M** in acetonitrile acquired using synchrotron radiation (with $\lambda = 0.56999$ and 0.69995 Å, respectively) and those acquired using Cu Kα radiation ($\lambda = 1.54184$ Å, the same patterns as those in Figure 1c in the main text). The patterns acquired with synchrotron radiation were converted to $\lambda = 1.54184$ Å for ease of comparison.

S4.2 Possible framework topologies and their structural models

Following the method reported by Nguyen,[S4] we used the *Reticular Chemistry Structure Resource* (RCSR)[S5] database to search for the potential topologies of **TK-COF-P** and **-M**. Our geometrical search was limited to the combinations of triangular and tetrahedral linkers. Because higher symmetry in the network topology constructed by connecting linkers (*i.e.*, fewer kinds of vertices and edges contained in the network) is generally preferred in the reticulation of linkers into an extended network,[S4] we put higher priority onto candidate topologies that consist of fewer kinds of vertices and edges in our search for the right topology.

Consequently, we could narrow the possible topologies down to ten types. Figure S24 illustrates the ten topologies, which are classified as cubic, tetragonal, or trigonal/hexagonal crystal systems. For the **bor** and **ctn** topologies, we considered both non-interpenetrated (denoted **bor** and **ctn**) and 1-fold interpenetrated (denoted **bor-c** and **ctn-c**) networks (Figure S24a). The characteristics of these candidate topologies are summarized in Table S5. Notably, as shown in this table, the space groups we used for our structural models had either the same or lower symmetry than the symmetries of the basic structures presented in the *RCSR* database: we had to reduce the symmetry of some structural models for the models to render chemically reasonable connections between **HABF** and **TAM** linkers. We checked that each structural model had a reasonable extended skeleton in the chosen space group. We did not consider topologies with lower symmetry than that of **asn** topology, because such topologies were implausibly complex; **asn** topology consists of six kinds of vertices *and* eight kinds of edges.

Subsequently, we constructed ten model frameworks with the candidate topologies and space groups listed in Table S5 using Materials Studio® software. Then, we generated the simulated PXRD patterns from these structural models and compared them with the experimental PXRD

patterns obtained from **TK-COF-P** and **-M** (Figures S25–S27). Consequently, we found that the simulated PXRD pattern generated from the structural model with **sln** topology agreed excellently with the experimental PXRD pattern acquired from **TK-COF-P** (Figure S28).

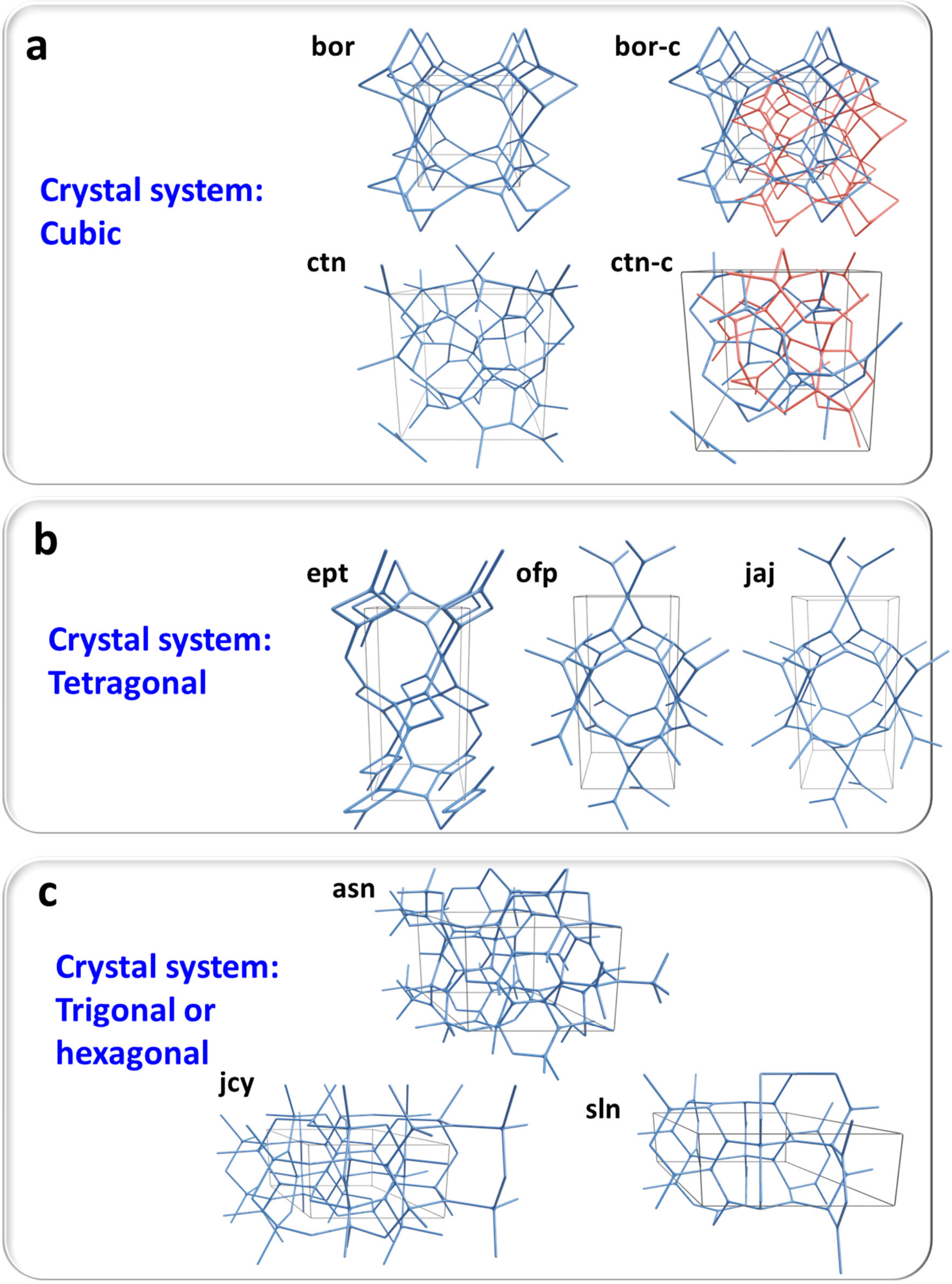

Figure S24. Potential topologies of **TK-COF-P/-M** synthesized in this study, categorized into (a) cubic, (b) tetragonal, and (c) trigonal/hexagonal crystal systems. Grey frames represent the unit cell. Skeletons in red represent the interpenetrated structures. All models are constructed based on the data reported in the *Reticular Chemistry Structure Resource* (RCSR).

Table S5. Structural and symmetrical characteristics of the candidate topologies in Figure S24.

Topology	Crystal system	Number of kinds of vertices	Number of kinds of edges	Space group displayed in *RCSR*	Space group for COF model
**bor**	Cubic	2	1	*P*–43*m* (No.215)	*P*23 (No. 195)
**bor-c**		2	1	*I*–43*m* (No.217)	*P*–4 (No. 81)
**ctn**		2	1	*I*–43*d* (No.220)	*I*–43*d* (No.220)
**ctn-c**		2	1	*Ia*–3*d* (No.230)	*Ia*–3*d* (No.230)
**ept**	Tetragonal	3	2	$P4_2/nnm$ (No.134)	$P2_1$ (No. 4)
**ofp**		3	2	*I*–42*m* (No.121)	*I*–4 (No. 82)
**jaj**		3	2	$P4_2/mnm$ (No.136)	*P*–1 (No. 2)
**jcy**	Trigonal or hexagonal	3	3	*P*–62*c* (No.190)	*P*3 (No. 143)
**asn**		6	8	*P*31*c* (No.159)	*P*31*c* (No.159)
**sln**		3	3	$P6_3/m$ (No.176)	$P6_3$ (No. 173)

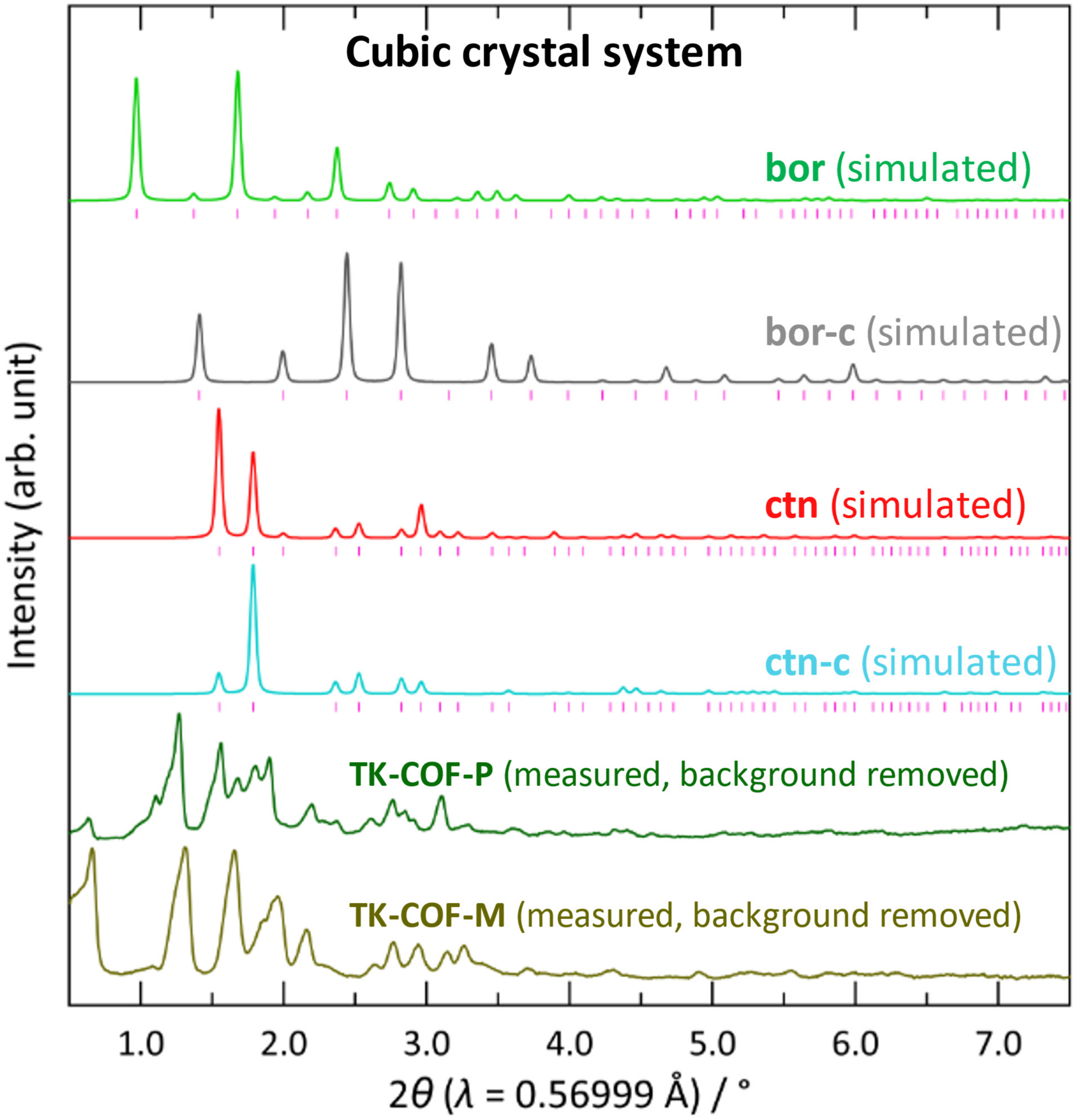

Figure S25. Comparison of the experimental PXRD patterns for **TK-COF-P/-M** with the simulated PXRD patterns of the structural models for the **bor**, **bor-c**, **ctn**, and **ctn-c** topologies, all with cubic space groups. The pink ticks under the simulated PXRD patterns indicate the Bragg positions for each structural model; see Table S5 for details.

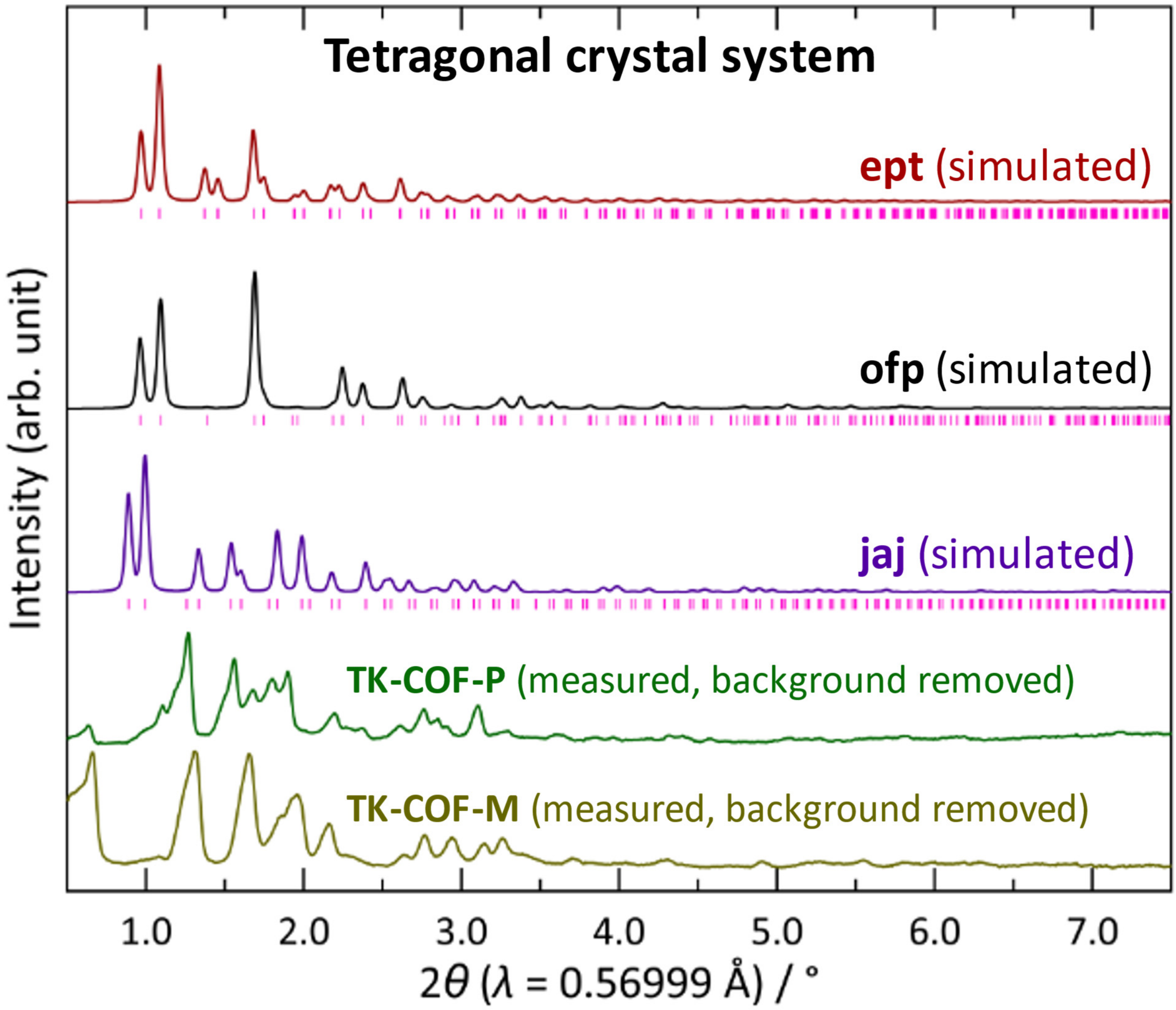

Figure S26. Comparison of the experimental PXRD patterns for **TK-COF-P/-M** with the simulated PXRD patterns of the structural models for the **ept**, **ofp**, and **jaj** topologies, all with tetragonal space groups. The pink ticks under the simulated PXRD patterns indicate the Bragg positions for each structural model; see Table S5 for details.

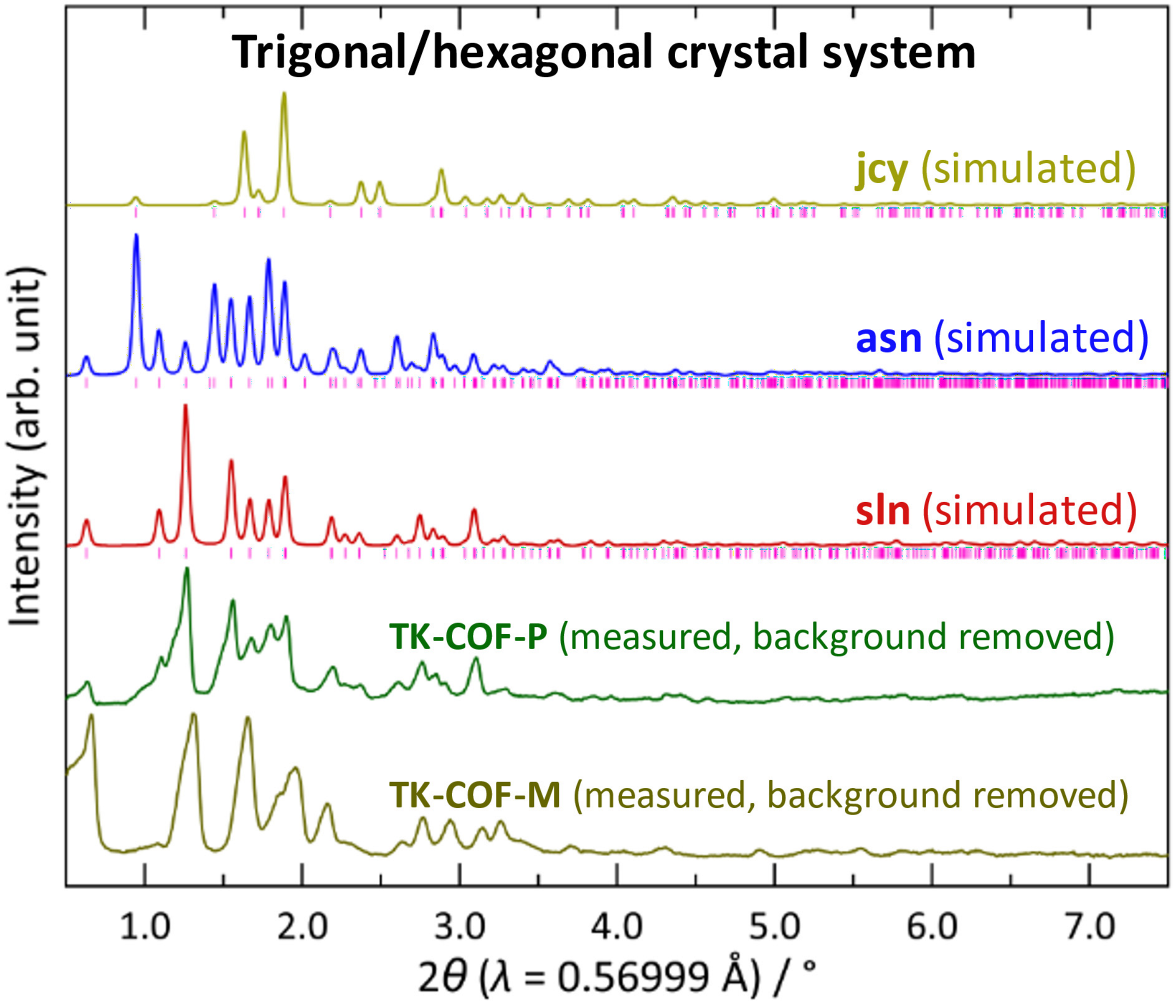

Figure S27. Comparison of the experimental PXRD patterns for **TK-COF-P/-M** with the simulated PXRD patterns of the structural models for the **jcy**, **asn**, and **sln** topologies, all with trigonal or hexagonal space groups. The pink ticks under the simulated PXRD patterns indicate the Bragg positions for each structural model; see Table S5 for details.

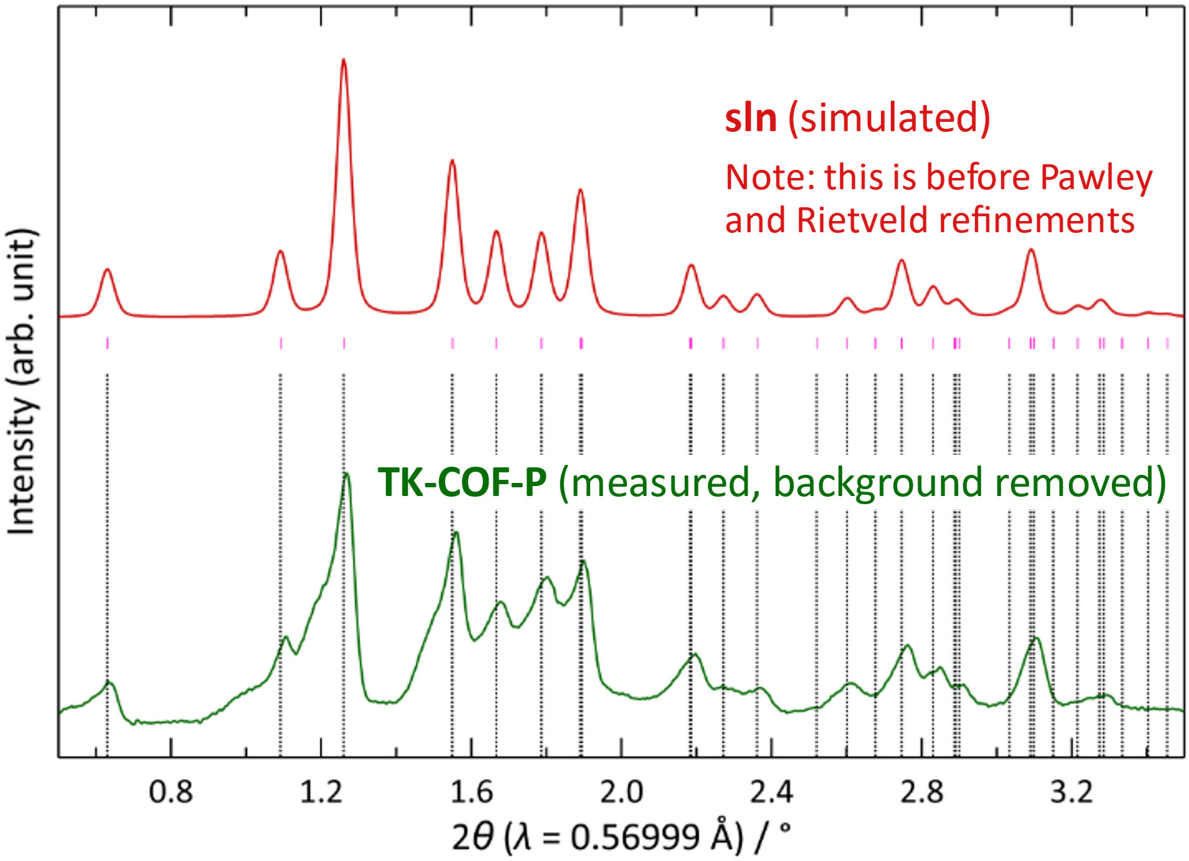

Figure S28. Agreement of the simulated PXRD pattern generated from the model with the **sln** topology and the experimental PXRD pattern from **TK-COF-P**. The pink ticks under the simulated PXRD pattern indicate the Bragg positions for the **sln** model; see Table S5 for details.

S4.3 Structural determination of **TK-COF-P**

Based on the excellent agreement between the simulated and experimental PXRD patterns shown in Figure S28, the model with **sln** topology was further refined using the *Reflex*® module of Materials Studio® software as follows.

First, we conducted Pawley refinement[S6] to optimize the unit cell parameters of the model. The refinement yielded sufficiently low R_{wp} (0.48%) and R_p (0.34%) values (Figure S29), from which the unit cell parameters for **TK-COF-P** have been determined to be $a = b = 59.660(3)$ Å, $c = 23.024(2)$ Å, $\alpha = \beta = 90°$, $\gamma = 120°$. Then, we conducted Rietveld refinement[S7] of the atomic coordinates in the unit cell using a *universal* force field[S8] with the "*Rietveld with energies*" option in Materials Studio® software. The result of the Rietveld refinements is presented in Figure 2a in the main text, showing satisfactorily low R_{wp} and R_p values (< 1.5%).

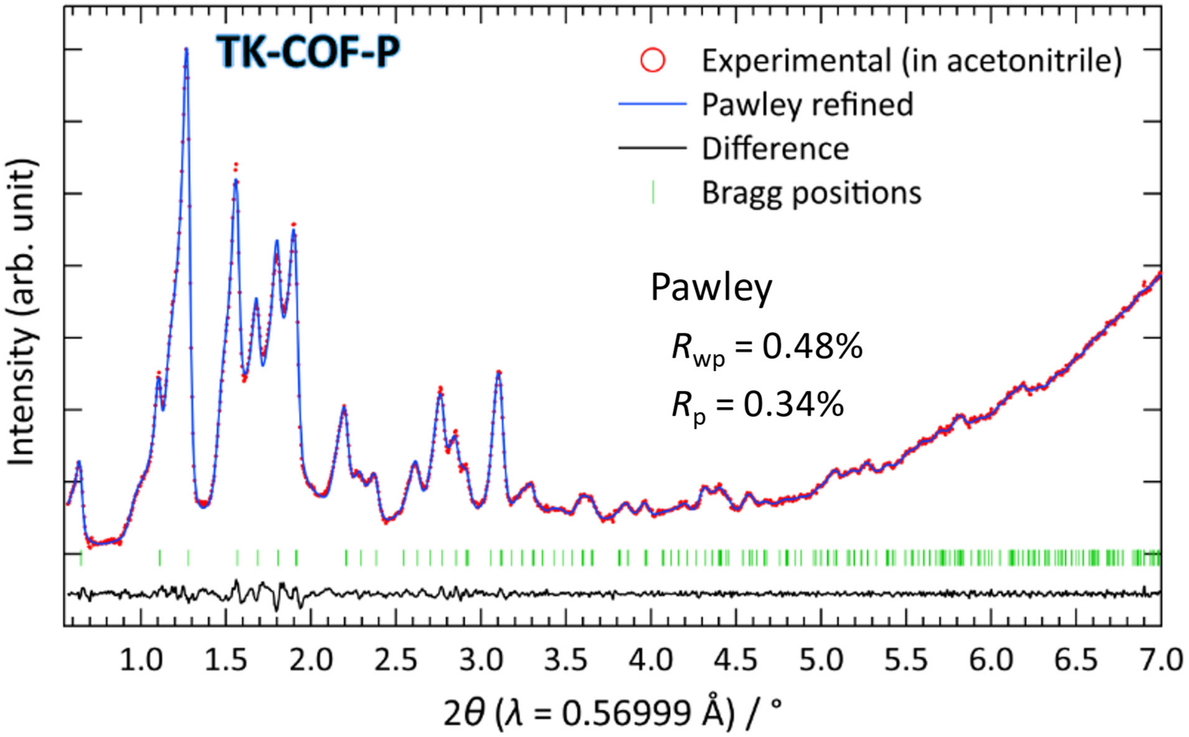

Figure S29. Result of the Pawley refinement of the unit cell for the PXRD pattern from **TK-COF-P** shown in Figure S21. The experimental pattern was obtained using synchrotron radiation with a wavelength of 0.56999 Å. The structural model with **sln** topology (see Figure S24c) was used for this refinement.

The framework topology was confirmed to be **sln** using ToposPro® software (Figure S30). The atomic coordinates determined by the Rietveld analysis are given in Table S6.

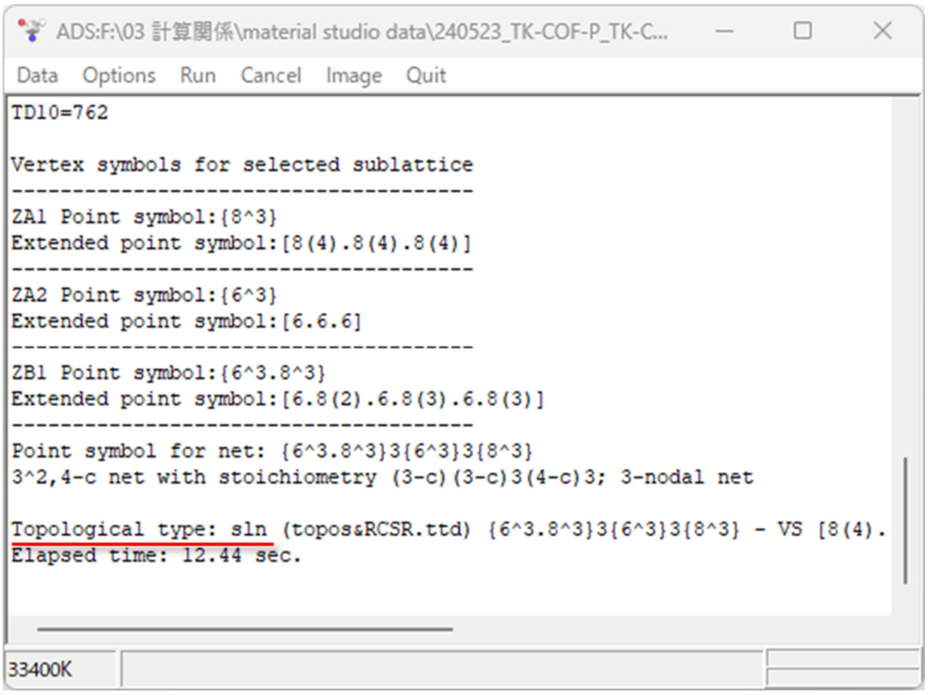

Figure S30. Topology judgment by ToposPro® on the determined structure of **TK-COF-P** (*cf.* Figure 2b), indicating the topological type of **sln** (underlined in red).

Table S6. Fractional atomic coordinates for the Rietveld-refined structure of **TK-COF-P**.

**TK-COF-P**							
Space group: $P6_3$, $a = b = 59.660(3)$ Å, $c = 23.024(2)$ Å, $\alpha = \beta = 90°$, $\gamma = 120°$							
**Atom**	***x***	***y***	***z***	**Atom**	***x***	***y***	***z***
C1	−0.39934	−0.22592	−0.29659	H74	−0.56112	−0.27176	−0.41258
C2	−0.42951	−0.23902	−0.29536	H75	−0.25085	0.05637	−0.47679
C3	−0.44578	−0.2628	−0.26711	H76	−0.25758	0.06911	−0.57431
C4	−0.47281	−0.27387	−0.26758	H77	−0.31219	0.08871	−0.50262
C5	−0.48471	−0.26252	−0.29882	H78	−0.30592	0.07567	−0.40619
C6	−0.46889	−0.23978	−0.3304	H79	−0.2231	0.09219	−0.23796
C7	−0.44188	−0.22864	−0.32965	H80	−0.20884	0.11937	−0.1506
N8	−0.51263	−0.2745	−0.29687	H81	−0.28813	0.08476	−0.09313
C9	−0.52708	−0.27094	−0.33397	H82	−0.30228	0.05767	−0.18108
C10	−0.55576	−0.28422	−0.32798	H83	−0.33993	−0.02589	−0.32674
C11	−0.56872	−0.29837	−0.27796	H84	−0.35602	−0.07246	−0.31364
C12	−0.5958	−0.31039	−0.27378	H85	−0.27789	−0.05597	−0.28236
C13	−0.61042	−0.30866	−0.31964	H86	−0.26208	−0.00959	−0.29488
C14	−0.5975	−0.29468	−0.36957	H87	−0.23702	0.17475	−0.67723
C15	−0.57051	−0.28249	−0.37354	H88	−0.26347	0.1375	−0.61342
C16	−0.63928	−0.32137	−0.31599	H89	−0.25938	0.08659	−0.73933
C17	−0.65476	−0.34866	−0.31553	H90	−0.23379	0.12286	−0.80299
C18	−0.28799	0.02565	−0.3718	H91	−0.31787	0.06166	−0.6031
C19	−0.29002	0.01408	−0.31676	H92	−0.41842	−0.19486	−0.24659
C20	−0.2823	0.02959	−0.26603	H93	−0.39822	−0.14789	−0.24733
C21	−0.27226	0.05664	−0.27002	H94	−0.32799	−0.14184	−0.31453
C22	−0.26995	0.06816	−0.3253	H95	−0.34817	−0.18859	−0.31552
C23	−0.27808	0.05277	−0.37641	H96	−0.31228	−0.10133	−0.28563
C24	−0.2781	0.06477	−0.43394	H97	−0.21711	0.13337	−0.05738
C25	−0.26374	0.07298	−0.21624	H98	−0.38256	−0.19588	−0.4009
C26	−0.29981	−0.01432	−0.31185	H99	−0.3712	−0.2069	−0.49301
C27	−0.26363	0.06424	−0.48179	H100	−0.39677	−0.28419	−0.43165
C28	−0.26697	0.07213	−0.53731	H101	−0.40813	−0.27316	−0.33805
C29	−0.2847	0.08085	−0.54583	C102	−0.64238	−0.36477	−0.31271
C30	−0.29765	0.08306	−0.4971	C103	−0.29621	0.00919	−0.4246
C31	−0.29438	0.0752	−0.44197	C104	−0.28401	0.01749	−0.20861
C32	−0.23741	0.09053	−0.20666	C105	−0.25862	0.09661	−0.32976
C33	−0.22931	0.10601	−0.15669	C106	−0.64359	−0.37791	−0.26136
C34	−0.24732	0.10434	−0.11497	C107	−0.63136	−0.39405	−0.2595
C35	−0.27375	0.08662	−0.12441	C108	−0.61878	−0.39582	−0.30885

C36	−0.28185	0.07109	−0.17456	C109	−0.61786	−0.38238	−0.35937
C37	−0.32633	−0.0324	−0.31725	C110	−0.62949	−0.36703	−0.36136
C38	−0.33549	−0.05895	−0.30941	F111	−0.63228	−0.40697	−0.21034
C39	−0.3182	−0.06787	−0.29657	F112	−0.65557	−0.37564	−0.21342
C40	−0.29167	−0.04968	−0.29197	C113	−0.23405	0.11202	−0.35485
C41	−0.28259	−0.02322	−0.29928	C114	−0.22342	0.13887	−0.35837
C42	−0.23616	0.15162	−0.74909	C115	−0.23721	0.1504	−0.33709
C43	−0.24165	0.1556	−0.69132	C116	−0.26161	0.13515	−0.31212
C44	−0.25611	0.13438	−0.65375	C117	−0.2723	0.10834	−0.30835
C45	−0.26373	0.10903	−0.6718	F118	−0.19966	0.15381	−0.38216
C46	−0.2539	0.10588	−0.72441	F119	−0.22113	0.10166	−0.37387
C47	−0.23992	0.12679	−0.76189	C120	−0.31941	0.00308	−0.45318
N48	−0.2878	0.0874	−0.65264	C121	−0.32687	−0.01248	−0.50297
C49	−0.29783	0.07696	−0.6026	C122	−0.31133	−0.02204	−0.52426
C50	−0.38589	−0.19611	−0.28646	C123	−0.28833	−0.01613	−0.49581
C51	−0.39892	−0.18378	−0.26317	C124	−0.28078	−0.0006	−0.44612
C52	−0.38722	−0.1567	−0.26246	C125	−0.26138	0.02121	−0.18117
C53	−0.36174	−0.14101	−0.28171	C126	−0.26283	0.00943	−0.12775
C54	−0.3477	−0.15306	−0.29923	C127	−0.28685	−0.00614	−0.10141
C55	−0.35944	−0.18011	−0.30028	C128	−0.30948	−0.00992	−0.12836
N56	−0.35142	−0.11337	−0.28592	C129	−0.30813	0.00187	−0.18183
C57	−0.32698	−0.09584	−0.28919	F130	−0.33481	0.01199	−0.43252
N58	−0.25348	0.12051	−0.02199	F131	−0.34923	−0.01839	−0.53062
C59	−0.2378	0.12108	−0.06226	F132	−0.33022	−0.00179	−0.20734
C60	−0.39395	−0.23277	−0.35872	F133	−0.33273	−0.02504	−0.10256
C61	−0.38485	−0.21495	−0.40542	H134	−0.6097	−0.40762	−0.30808
C62	−0.37845	−0.2214	−0.45921	H135	−0.60806	−0.38388	−0.39744
C63	−0.3817	−0.24619	−0.46876	H136	−0.62855	−0.35691	−0.40116
C64	−0.39321	−0.26473	−0.42476	H137	−0.22897	0.17117	−0.33982
C65	−0.3996	−0.2583	−0.37108	H138	−0.27224	0.14411	−0.29562
H66	−0.43799	−0.27346	−0.24548	H139	−0.29123	0.0967	−0.28893
H67	−0.48461	−0.29176	−0.24426	H140	−0.31712	−0.03408	−0.56274
H68	−0.4771	−0.23024	−0.35516	H141	−0.27634	−0.02359	−0.51229
H69	−0.43046	−0.21141	−0.35538	H142	−0.26289	0.0039	−0.42448
H70	−0.51847	−0.25882	−0.3717	H143	−0.24268	0.0331	−0.2014
H71	−0.55794	−0.3	−0.24197	H144	−0.24534	0.01232	−0.10684
H72	−0.60535	−0.32095	−0.2347	H145	−0.2879	−0.01526	−0.06007
H73	−0.60839	−0.29323	−0.40559				

S4.4 Structural determination of **TK-COF-M**

Because the lowest-angle peaks for **TK-COF-P** and **-M** were seen at the same 2θ position (Figure S23), we surmise that **TK-COF-M** also has **sln** topology. However, Pawley refinement of the unit cell using the $P6_3$ space group (*i.e.*, high symmetry) disagreed with the experimental PXRD pattern of **TK-COF-M** (Figure S31a), suggesting that the symmetry for the model for **TK-COF-M** should be lowered from $P6_3$.

Then, to determine the unit cell parameters for **TK-COF-M**, we constructed a structural model with **sln** topology and $P2_1$ space group, which has a lower symmetry than $P6_3$. As a result, the Pawley refinement yielded sufficiently low R_{wp} (0.63%) and R_p (0.45%) values (Figure S31b), from which the unit cell parameters for **TK-COF-M** have been determined to be a = 58.261(7) Å, b = 17.392(3) Å, c = 60.335(6) Å, α = 90°, β = 116.332(10)°, γ = 90°.

Using these unit cell parameters and following the procedure described in Section S4.3, we conducted Rietveld refinement of the atomic coordinates in the unit cell. Because **TK-COF-M** has membranous (*i.e.*, macroscopically anisotropic) morphology, we adopted the *"preferred orientation"* option during the refinement. The result of the Rietveld refinement and the refined structural model are shown in Figure 2c and 2d in the main text, respectively. The framework topology was confirmed to be **sln** using ToposPro® software (Figure S32). The atomic coordinates determined by the Rietveld analysis are given in Table S7.

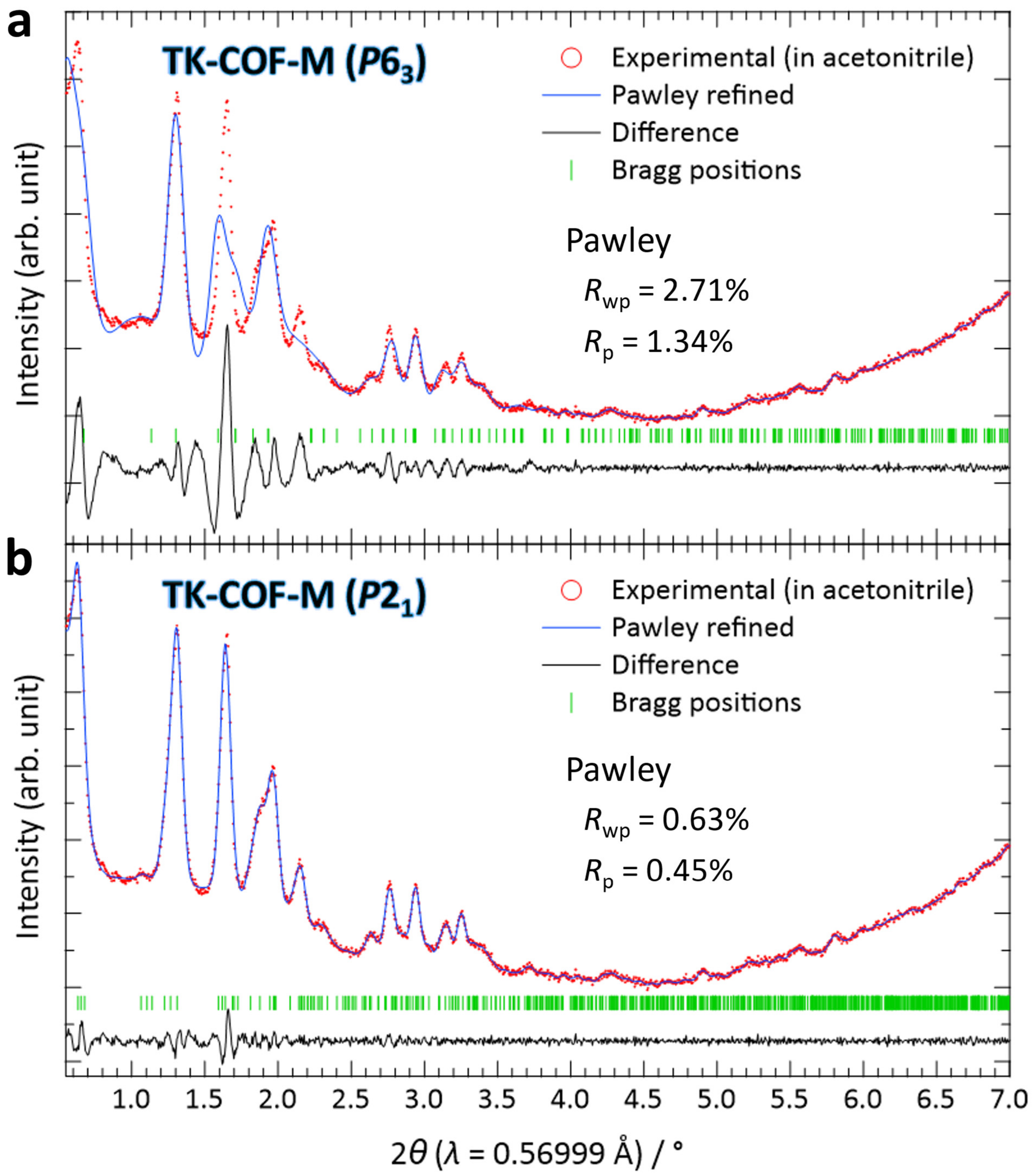

Figure S31. Results of the Pawley refinement for the experimental PXRD pattern of **TK-COF-M** using the **sln** structural model with (a) $P6_3$ and (b) $P2_1$ space groups. The experimental PXRD pattern was collected by synchrotron radiation at a wavelength of 0.69995 Å and converted to a wavelength of 0.56999 Å in this graph.

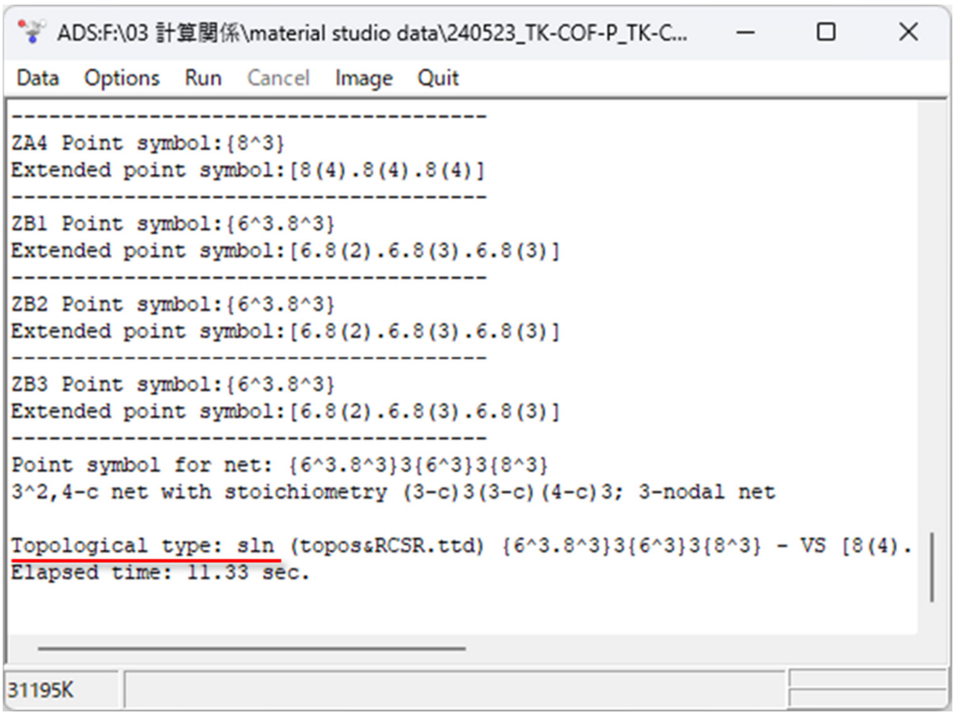

Figure S32. Topology judgement made by ToposPro® on the determined structure of **TK-COF-M** (cf. Figure 2d), indicating the topological type of **sln** (underlined in red).

Table S7. Fractional atomic coordinates for the Rietveld-refined structure of **TK-COF-M**.

**TK-COF-M**							
Space group: $P2_1$, a = 58.261(7) Å, b = 17.392(3) Å, c = 60.335(6) Å, α = 90°, β = 116.332(10)°, γ = 90°							
**Atom**	***x***	***y***	***z***	**Atom**	***x***	***y***	***z***
C1	1.25155	0.37373	0.481	C219	0.86483	−0.25915	0.21212
C2	1.39307	0.79924	0.22472	N220	0.90777	−0.15273	0.26966
C3	0.82484	−0.3408	0.20083	C221	0.93125	−0.1599	0.27285
C4	0.98637	0.15186	0.30766	C222	1.24845	0.46207	0.48077
C5	0.99952	0.0829	0.31878	C223	1.26731	0.50796	0.49924
C6	1.02615	0.08541	0.33462	C224	1.26994	0.58568	0.49511
C7	1.0392	0.15663	0.33994	C225	1.25406	0.61929	0.47221
C8	1.02596	0.22522	0.32915	C226	1.23367	0.57686	0.45489
C9	0.99967	0.22264	0.31273	C227	1.2311	0.49904	0.45885
C10	0.98543	0.29393	0.3011	N228	1.26032	0.69061	0.46425
C11	1.03964	0.30003	0.3351	C229	1.28332	0.71865	0.47256
C12	1.06684	0.16004	0.35704	C230	1.39173	0.88706	0.21979
C13	1.04007	0.01466	0.3473	C231	1.36901	0.92632	0.21597
C14	0.98603	0.01261	0.31302	C232	1.3623	0.99436	0.20248
C15	0.95892	0.14961	0.2891	C233	1.3782	1.02429	0.19266
C16	0.9744	0.30261	0.27531	C234	1.40309	0.99569	0.20117
C17	0.95535	0.3576	0.26365	C235	1.41013	0.92816	0.21521
C18	0.94757	0.40574	0.27776	N236	1.36819	1.07342	0.17137
C19	0.96048	0.40204	0.30367	C237	1.34472	1.06763	0.15427
C20	0.97922	0.34614	0.31529	C238	1.38809	0.79106	0.24783
C21	1.0453	0.33787	0.31753	C239	1.40463	0.82916	0.26986
C22	1.05797	0.40876	0.32328	C240	1.39826	0.83758	0.28948
C23	1.0651	0.44196	0.34652	C241	1.37502	0.80954	0.28767
C24	1.05953	0.4045	0.36404	C242	1.35899	0.76767	0.26647
C25	1.0468	0.33383	0.35836	C243	1.36527	0.75933	0.24677
C26	1.08481	0.16585	0.3476	N244	1.3678	0.82963	0.30688
C27	1.11077	0.17477	0.36361	C245	1.3451	0.82288	0.30582
C28	1.11897	0.17984	0.38922	C246	0.62742	0.25788	−0.20144
C29	1.10096	0.17241	0.39869	C247	0.63081	0.17752	−0.20178
C30	1.07505	0.16191	0.38266	C248	0.64713	0.1379	−0.18034
C31	1.05765	−0.02057	0.3404	C249	0.6597	0.17681	−0.15781
C32	1.0707	−0.08697	0.35262	C250	0.65662	0.25633	−0.15712
C33	1.0663	−0.11837	0.37173	C251	0.64052	0.2959	−0.17836
C34	1.04882	−0.0836	0.37862	N252	0.67469	0.13397	−0.13577
C35	1.03571	−0.01733	0.36646	C253	0.6779	0.1534	−0.11376
C36	0.99228	−0.04509	0.3003	C254	0.84184	0.58638	0.2087
C37	0.97499	−0.10458	0.28855	C255	0.85023	0.55907	0.23314
C38	0.95134	−0.1073	0.28943	C256	0.87144	0.51102	0.24405
C39	0.94631	−0.05436	0.30428	C257	0.88523	0.48947	0.23101
C40	0.96359	0.00472	0.31609	C258	0.87598	0.51049	0.20617
C41	0.95206	0.11642	0.26579	C259	0.85474	0.55917	0.19516
C42	0.92644	0.1148	0.24815	N260	0.9089	0.44784	0.24278
C43	0.90751	0.14649	0.25362	C261	0.92489	0.45551	0.26602
C44	0.91414	0.17951	0.27679	C262	1.26926	0.36466	0.4681
C45	0.93975	0.1809	0.29457	C263	1.22558	0.33228	0.4677
F46	1.06338	0.44542	0.30633	C264	1.20364	0.36034	0.46935
F47	1.03863	0.30592	0.29498	C265	1.17994	0.32436	0.45663
F48	1.06177	0.00862	0.32161	C266	1.17761	0.25922	0.44226
F49	1.08757	−0.1212	0.3459	C267	1.19975	0.22585	0.44268
F50	0.89569	0.21001	0.28202	C268	1.22355	0.26114	0.4558
F51	0.94586	0.2117	0.31717	C269	1.2589	0.36152	0.442

C52	1.31375	0.90252	0.39608	C270	1.27411	0.37588	0.42994
C53	1.30622	0.93502	0.41328	C271	1.29978	0.39732	0.44343
C54	1.30485	1.01554	0.41516	C272	1.31055	0.39927	0.4692
C55	1.31182	1.0641	0.40034	C273	1.29566	0.38202	0.48142
C56	1.31926	1.03154	0.38297	N274	1.31358	0.42872	0.43089
C57	1.31996	0.9507	0.38069	C275	1.3185	0.39238	0.41465
C58	1.3269	0.91612	0.36198	N276	1.15244	0.23061	0.42673
C59	1.32625	1.08192	0.36704	C277	1.14598	0.19808	0.40542
C60	1.31243	1.14881	0.40395	C278	0.81377	−0.3235	0.17286
C61	1.2951	1.04866	0.43205	C279	0.81424	−0.24897	0.16372
C62	1.30019	0.88438	0.42971	C280	0.80314	−0.2344	0.13835
C63	1.31536	0.81767	0.39433	C281	0.79003	−0.29223	0.12138
C64	1.35208	0.92071	0.36476	C282	0.78852	−0.36601	0.13013
C65	1.35837	0.88912	0.34683	C283	0.80011	−0.3812	0.15554
C66	1.3395	0.8525	0.32586	N284	0.77886	−0.27424	0.09549
C67	1.31443	0.84715	0.32338	C285	0.75831	−0.30575	0.07864
C68	1.30818	0.87871	0.34128	C286	1.33744	0.19353	0.67244
C69	1.34905	1.12497	0.37717	C287	1.33686	0.27302	0.66857
C70	1.3552	1.17305	0.36199	C288	1.32494	0.30268	0.64466
C71	1.3388	1.17806	0.33671	C289	1.31378	0.25319	0.62428
C72	1.31621	1.13502	0.32652	C290	1.31448	0.17331	0.62818
C73	1.30992	1.08711	0.3416	C291	1.32616	0.14368	0.65221
C74	1.29736	1.19818	0.38446	H292	0.97919	0.26298	0.26414
C75	1.2992	1.27791	0.38789	H293	0.94597	0.36011	0.24365
C76	1.31534	1.30873	0.41108	H294	0.95493	0.43913	0.31489
C77	1.32994	1.25954	0.43068	H295	0.98752	0.34007	0.3352
C78	1.32875	1.18025	0.42705	H296	1.07491	0.49677	0.35095
C79	1.30997	1.0467	0.45786	H297	1.06502	0.43032	0.38201
C80	1.29986	1.07428	0.47348	H298	1.04241	0.30558	0.37202
C81	1.27491	1.1033	0.46347	H299	1.07864	0.16487	0.32781
C82	1.26017	1.10605	0.43783	H300	1.12432	0.18069	0.35594
C83	1.27025	1.07921	0.42216	H301	1.10685	0.17592	0.41839
C84	1.27476	0.87359	0.42556	H302	1.06127	0.15765	0.39013
C85	1.26908	0.82227	0.44024	H303	1.0764	−0.16974	0.38117
C86	1.28885	0.78152	0.45932	H304	1.04542	−0.10802	0.3934
C87	1.31436	0.79512	0.46405	H305	1.02224	0.00926	0.37198
C88	1.31998	0.84593	0.44926	H306	1.00928	−0.04027	0.29739
C89	1.3394	0.78176	0.4035	H307	0.97933	−0.1453	0.27734
C90	1.3411	0.7019	0.40209	H308	0.92844	−0.05601	0.30572
C91	1.31884	0.65744	0.39152	H309	0.9587	0.04724	0.32642
C92	1.29482	0.69281	0.38224	H310	0.9666	0.09214	0.26129
C93	1.29301	0.77276	0.38358	H311	0.92127	0.08916	0.23022
F94	1.37711	1.21488	0.37178	H312	0.88769	0.14531	0.23989
F95	1.36527	1.11997	0.40164	H313	1.36676	0.94911	0.38073
F96	1.33416	1.01813	0.4678	H314	1.37785	0.89426	0.34923
F97	1.31413	1.07223	0.49837	H315	1.29956	0.81944	0.30737
F98	1.27327	0.64946	0.37187	H316	1.28866	0.87481	0.33888
F99	1.26958	0.80651	0.3742	H317	1.34358	1.21524	0.32498
C100	0.71728	−0.13142	−0.02967	H318	1.30353	1.13893	0.30694
C101	0.71737	−0.2103	−0.0236	H319	1.29228	1.0544	0.33354
C102	0.70696	−0.26574	−0.04276	H320	1.28451	1.17501	0.36655
C103	0.69553	−0.24196	−0.06771	H321	1.28797	1.31548	0.37257
C104	0.69542	−0.1637	−0.07367	H322	1.34281	1.28274	0.44859
C105	0.70692	−0.10871	−0.05468	H323	1.34086	1.14334	0.44218
C106	0.70688	−0.02724	−0.06183	H324	1.26697	1.12323	0.4756
C107	0.68342	−0.13916	−0.10009	H325	1.24083	1.12818	0.43005
C108	0.68453	−0.29855	−0.08806	H326	1.25854	1.08074	0.40233
C109	0.70841	−0.34901	−0.03686	H327	1.25933	0.90333	0.41056

C110	0.72812	−0.23497	0.00272	H328	1.24924	0.8128	0.4362
C111	0.72757	−0.07216	−0.00999	H329	1.32988	0.76411	0.47858
C112	0.68868	0.02332	−0.06073	H330	1.33971	0.85431	0.45273
C113	0.68101	0.08777	−0.07613	H331	1.35678	0.81565	0.41181
C114	0.69115	0.10075	−0.09299	H332	1.35968	0.67445	0.40923
C115	0.7116	0.05562	−0.09179	H333	1.32022	0.59559	0.39049
C116	0.71984	−0.00721	−0.07583	H334	0.6785	0.00853	−0.05004
C117	0.65979	−0.10032	−0.11031	H335	0.66535	0.12261	−0.0767
C118	0.64915	−0.07527	−0.13489	H336	0.71994	0.06567	−0.10441
C119	0.66185	−0.08954	−0.14943	H337	0.73406	−0.04559	−0.07669
C120	0.68517	−0.12893	−0.13947	H338	0.65359	−0.07024	−0.16845
C121	0.69596	−0.15368	−0.11489	H339	0.69495	−0.14	−0.15076
C122	0.65817	−0.29801	−0.10386	H340	0.71417	−0.18346	−0.10727
C123	0.64837	−0.3413	−0.12558	H341	0.6454	−0.26169	−0.09981
C124	0.66484	−0.3865	−0.1315	H342	0.62804	−0.33885	−0.13779
C125	0.69102	−0.38955	−0.11508	H343	0.70399	−0.42464	−0.11922
C126	0.70084	−0.34546	−0.09356	H344	0.72127	−0.34563	−0.08166
C127	0.73208	−0.38755	−0.02709	H345	0.71167	−0.56801	−0.02296
C128	0.7332	−0.46638	−0.02208	H346	0.66988	−0.50059	−0.04028
C129	0.71079	−0.50701	−0.02681	H347	0.6676	−0.36143	−0.04925
C130	0.68721	−0.46901	−0.03658	H348	0.69249	−0.28305	−0.0006
C131	0.68598	−0.39029	−0.04163	H349	0.71017	−0.32549	0.04262
C132	0.71251	−0.27253	0.01158	H350	0.78382	−0.2323	0.05588
C133	0.72257	−0.29651	0.03621	H351	0.76637	−0.19332	0.01239
C134	0.74825	−0.28176	0.05254	H352	0.76041	−0.04621	−0.01646
C135	0.76387	−0.24384	0.04371	H353	0.77738	0.05348	0.01602
C136	0.75394	−0.22133	0.01894	H354	0.75447	0.08487	0.041
C137	0.75023	−0.03291	−0.00565	H355	1.41794	0.23116	0.7736
C138	0.75988	0.02346	0.01271	H356	1.46402	0.25525	0.79048
C139	0.74695	0.04113	0.02681	H357	1.47011	0.07777	0.74333
C140	0.72431	0.00255	0.02253	H358	1.42411	0.05191	0.7272
C141	0.71456	−0.05395	0.00416	H359	1.43229	0.24674	0.66343
F142	0.62651	−0.03711	−0.14467	H360	1.40939	0.12199	0.65344
F143	0.64708	−0.08717	−0.0965	H361	1.38394	0.08191	0.67508
F144	0.75391	−0.34904	−0.02286	H362	1.23268	0.15083	0.66093
F145	0.756	−0.50365	−0.0128	H363	1.25579	0.02652	0.66922
F146	0.71175	0.02026	0.03614	H364	1.30302	0.02331	0.69202
F147	0.69235	−0.09015	−0.00009	H365	1.33342	−0.01158	0.74941
C148	1.37443	0.11767	0.74806	H366	1.30311	−0.02724	0.76738
C149	1.34744	0.12293	0.73551	H367	1.29569	0.21971	0.76876
C150	1.33529	0.14408	0.71032	H368	1.32587	0.23443	0.75101
C151	1.35008	0.16324	0.69795	H369	1.37819	0.19595	0.78855
C152	1.37701	0.16016	0.71054	H370	1.39952	0.15329	0.8321
C153	1.38928	0.13673	0.73559	H371	1.42085	0.02616	0.84283
C154	1.41767	0.13997	0.74913	H372	0.80001	−0.47266	0.20723
C155	1.39229	0.18456	0.69755	H373	0.76332	−0.47953	0.21559
C156	1.30692	0.14658	0.69685	H374	0.75513	−0.23293	0.20927
C157	1.33168	0.11248	0.74892	H375	0.79277	−0.22533	0.20133
C158	1.38714	0.09215	0.77421	H376	0.72778	−0.47299	0.219
C159	1.42937	0.19563	0.76756	H377	1.24271	0.41533	0.52084
C160	1.4556	0.20974	0.77701	H378	1.26066	0.36117	0.56219
C161	1.47045	0.16929	0.76789	H379	1.30127	0.19171	0.5412
C162	1.45903	0.11037	0.75048	H380	1.28329	0.24573	0.49943
C163	1.43284	0.09537	0.74134	H381	1.41295	0.79066	0.1911
C164	1.40513	0.25562	0.70302	H382	1.45282	0.73696	0.19518
C165	1.41958	0.27776	0.69075	H383	1.47143	0.66482	0.26953
C166	1.42113	0.22954	0.67293	H384	1.43083	0.71476	0.26531
C167	1.40821	0.15916	0.66728	H385	1.29877	0.34838	0.59763

C168	1.39381	0.13668	0.67952	H386	1.49469	0.73554	0.20991
C169	1.29376	0.21699	0.69224	H387	0.83017	−0.26914	0.24517
C170	1.26702	0.21835	0.67927	H388	0.86575	−0.19187	0.27366
C171	1.25336	0.14973	0.67096	H389	0.90111	−0.20369	0.22445
C172	1.26639	0.0796	0.67563	H390	0.8656	−0.27952	0.19551
C173	1.29309	0.07793	0.68855	H391	0.93607	−0.2014	0.26219
C174	1.3255	0.03886	0.75401	H392	1.28162	0.48266	0.51608
C175	1.30822	0.03013	0.76417	H393	1.28542	0.61777	0.50925
C176	1.29693	0.09489	0.76928	H394	1.22129	0.60209	0.43715
C177	1.30395	0.16864	0.76493	H395	1.21691	0.46644	0.44369
C178	1.32119	0.17723	0.75484	H396	1.29943	0.68848	0.48668
C179	1.3873	0.14005	0.79301	H397	1.35482	0.89936	0.2204
C180	1.39942	0.11616	0.81767	H398	1.34357	1.0188	0.19757
C181	1.41147	0.04448	0.82371	H399	1.41614	1.02102	0.19487
C182	1.41141	−0.00351	0.80509	H400	1.42909	0.90577	0.22034
C183	1.39929	0.02011	0.78037	H401	1.3319	1.02504	0.15578
F184	1.43204	0.34616	0.69608	H402	1.42202	0.85604	0.27132
F185	1.40344	0.30343	0.72002	H403	1.41091	0.86979	0.30568
F186	1.30678	0.28388	0.70049	H404	1.34114	0.74318	0.26446
F187	1.25429	0.28624	0.67476	H405	1.35116	0.73208	0.2303
F188	1.4231	−0.07305	0.81103	H406	1.32929	0.80026	0.28951
F189	1.39922	−0.02719	0.76244	H407	0.62045	0.14535	−0.2187
C190	0.80092	−0.34758	0.20557	H408	0.64924	0.07624	−0.18109
C191	0.79107	−0.41926	0.20834	H409	0.66634	0.28819	−0.14005
C192	0.76941	−0.42346	0.21263	H410	0.6378	0.35599	−0.17586
C193	0.75642	−0.3561	0.2134	H411	0.66768	0.20188	−0.11115
C194	0.76508	−0.28532	0.20906	H412	0.84145	0.57963	0.24452
C195	0.7867	−0.28109	0.20475	H413	0.87754	0.49304	0.263
N196	0.73525	−0.35602	0.21959	H414	0.88626	0.4933	0.1958
C197	0.72302	−0.41528	0.22215	H415	0.84994	0.58	0.17675
C198	1.41896	0.76343	0.22846	H416	0.92112	0.49654	0.27753
C199	1.26323	0.33942	0.50721	H417	1.20462	0.41154	0.47992
C200	1.25605	0.368	0.5251	H418	1.16313	0.34922	0.45713
C201	1.26629	0.33692	0.54882	H419	1.19867	0.173	0.43281
C202	1.2834	0.27474	0.55509	H420	1.24032	0.23431	0.45596
C203	1.28879	0.24133	0.53685	H421	1.23859	0.35403	0.43062
C204	1.27873	0.27295	0.51311	H422	1.26535	0.37655	0.40991
C205	1.4256	0.76444	0.20856	H423	1.33024	0.41734	0.47979
C206	1.4487	0.73392	0.21095	H424	1.30439	0.38874	0.5013
C207	1.46591	0.70006	0.23329	H425	1.3273	0.42346	0.40489
C208	1.45873	0.69303	0.25246	H426	1.15975	0.19298	0.39795
C209	1.43551	0.72345	0.25008	H427	0.82278	−0.20097	0.17603
N210	1.29534	0.24444	0.57974	H428	0.80415	−0.17703	0.13185
C211	1.30148	0.2866	0.59932	H429	0.77873	−0.41216	0.1174
N212	1.49084	0.67335	0.23739	H430	0.79846	−0.43839	0.16179
C213	1.50289	0.69366	0.22462	H431	0.74741	−0.34645	0.08376
C214	0.84392	−0.27841	0.21699	H432	1.34556	0.31196	0.68411
C215	0.84471	−0.25278	0.23958	H433	1.32461	0.36437	0.64206
C216	0.86544	−0.2099	0.25635	H434	1.30588	0.13399	0.61274
C217	0.88635	−0.19412	0.2516	H435	1.32654	0.08212	0.65506
C218	0.88559	−0.21726	0.22902				

S4.5 Comparison of the dihedral angles

Table S8. Comparison of the dihedral angles in **TK-COF-P** and **-M**.

Dihedral angles shown in Figure 2e	Dihedral angles (φ)	
	**TK-COF-P**	**TK-COF-M**
**A**	56.63°	−129.76°
**B**		−94.05°
**C**		70.64°

S4.6 Comparison of stripe periods in the HR-TEM image with the structural models

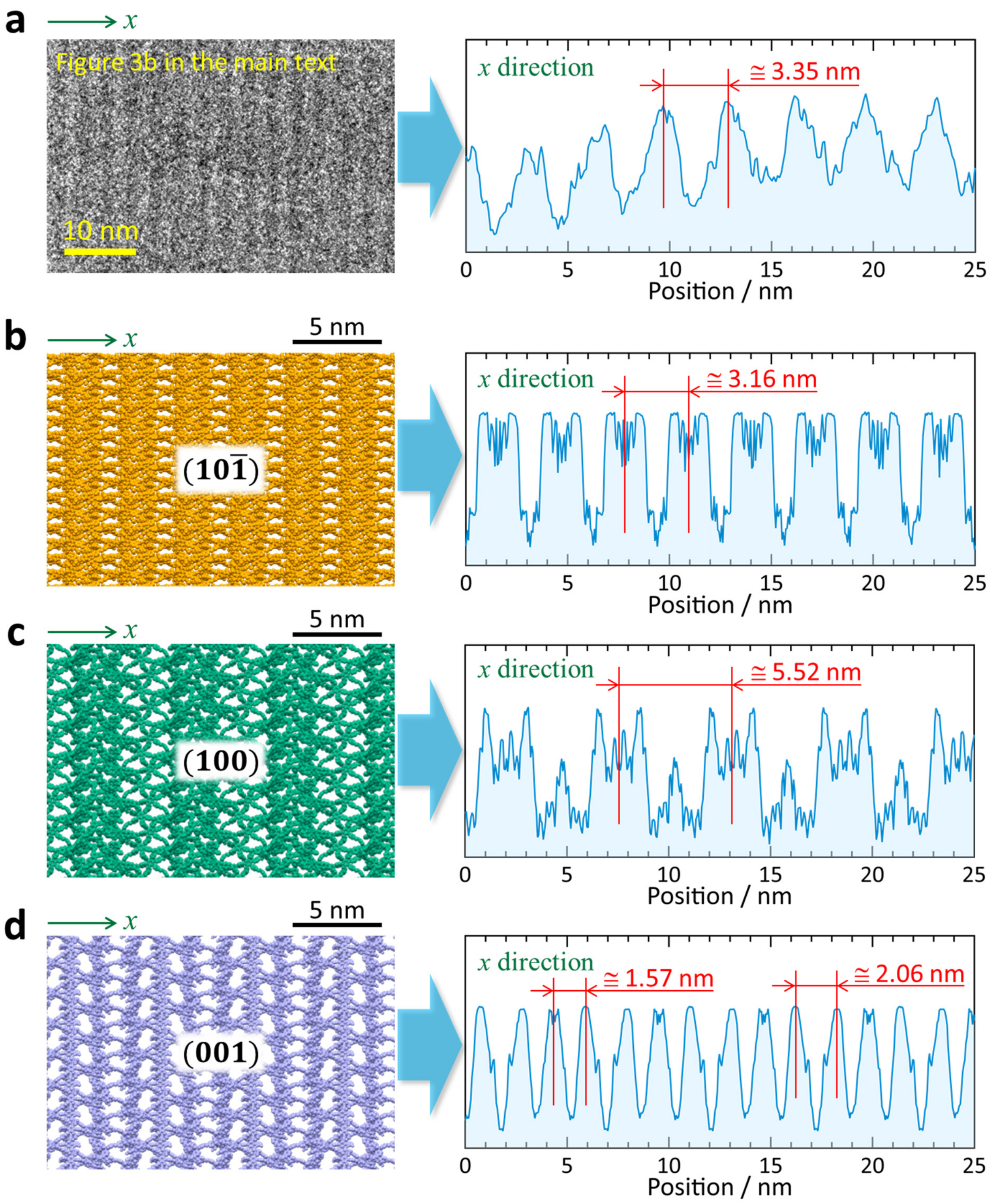

Figure S33. Comparison of stripe period seen in (a) the HR-TEM images of **TK-COF-M** (see Figure 3b in the main text) with the stripe periods that would be seen on the (b) (10$\bar{1}$), (c) (100), and (d) (001) planes of the structure determined by the Rietveld analysis of **TK-COF-M**.

Section S5. Discussion of energetic stabilities of TK-COF-P/-M

S5.1 Transformation of **TK-COF-P** to **-M** after a long retention in solution

We found that **TK-COF-P** transformed to **-M** after a long retention in solution. In the top photographs of Figure S34, **TK-COF-P** crystals were seen after 7 days of crystal growth at 22 °C. As shown by the bottom of the figure, however, these crystals had transitioned to **TK-COF-M** after retention in the same solution for further 14 weeks at 22 °C. This observation indicates that **TK-COF-M** is a thermodynamic product.

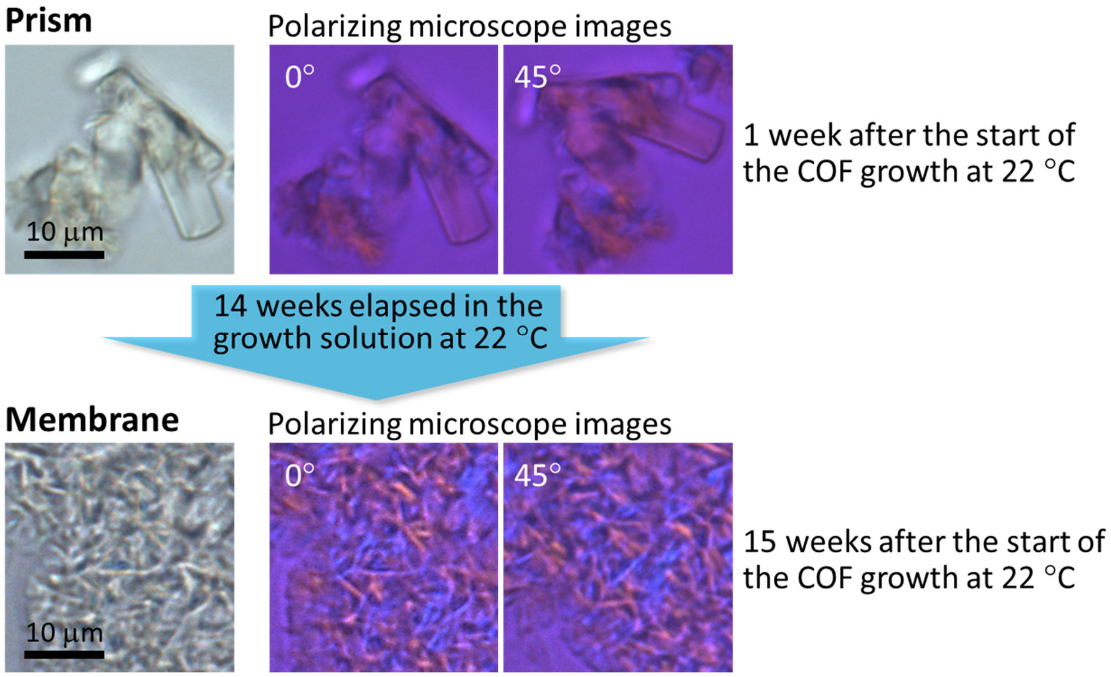

Figure S34. Optical micrographs taken before (top) and after (bottom) the transformation of **TK-COF-P** to **-M** through a retention of the former in the same growth solution for 14 weeks at 22 °C. The polarized optical micrographs were taken with a crossed-Nicols configuration.

S5.2 Energy calculations of structural moieties

To find out which of the two structures—**TK-COF-P** or **-M**—is more thermodynamically stable, we calculated and compared the energies of their structural moieties using Gaussian® software at the ωB97XD/6-31G+(d,p) level of theory. First, as a reference structural moiety, we constructed a model moiety by linking partial structures of **HABF** and **TAM** by an imine bond and then optimizing the geometry without geometrical restrictions (Figure S35). The energy of this reference moiety was set to be $H = 0$ kJ mol^{-1}. The energies of all structural moieties calculated below are presented with the symbol ΔH, which is defined as the difference from the energy of the reference moiety.

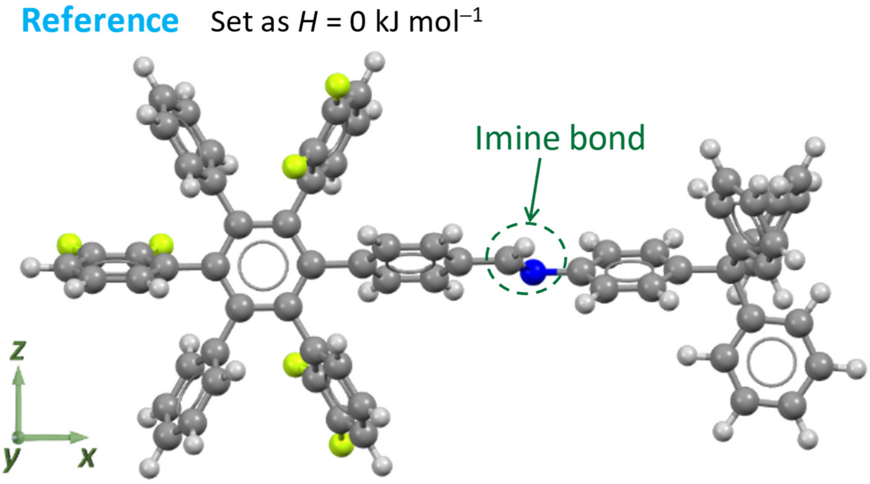

Figure S35. The optimized structure of the reference moiety calculated using Gaussian® software at the ωB97XD/6-31G+(d,p) level of theory. The color assignments are gray for C, yellow for F, blue for N, and white for H.

We calculated the energies of the structural moieties of **TK-COF-P** and **-M** as follows. The skeleton of **TK-COF-P**, determined by the Rietveld refinement (Section S4.3), contained four non-equivalent imine bonds. We extracted these four imine bonds with their adjacent **HABF** and **TAM** units to construct their structural moieties, the ends of which have been terminated with H atoms, referred to as $\mathbf{P}_{n}$ moieties for **TK-COF-P** ($n = 1$ to 4; Figure S36). We calculated the energy

of these moieties without geometrical optimizations. The skeleton of **TK-COF-M**, determined by the Rietveld refinement (Section S4.4), contained twelve non-equivalent imine bonds. Similarly, we constructed their structural moieties for **TK-COF-M**, referred to as $\mathbf{M}_n$ moieties (n = 1 to 12; Figures S37−S39) and calculated the energy of these moieties without geometrical optimizations.

The calculated values of ΔH are shown in Figures S36−S39. The results have been summarized in Figure S40, where "mol" is defined in terms of the number of the structural moieties. As this summary indicates, the energies of the $\mathbf{P}_n$ moieties (n = 1 to 4) for **TK-COF-P** are greater than those of the $\mathbf{M}_n$ moieties (n = 1 to 12) for **TK-COF-M**, indicating that **TK-COF-M** is more energetically stable than **TK-COF-P**.

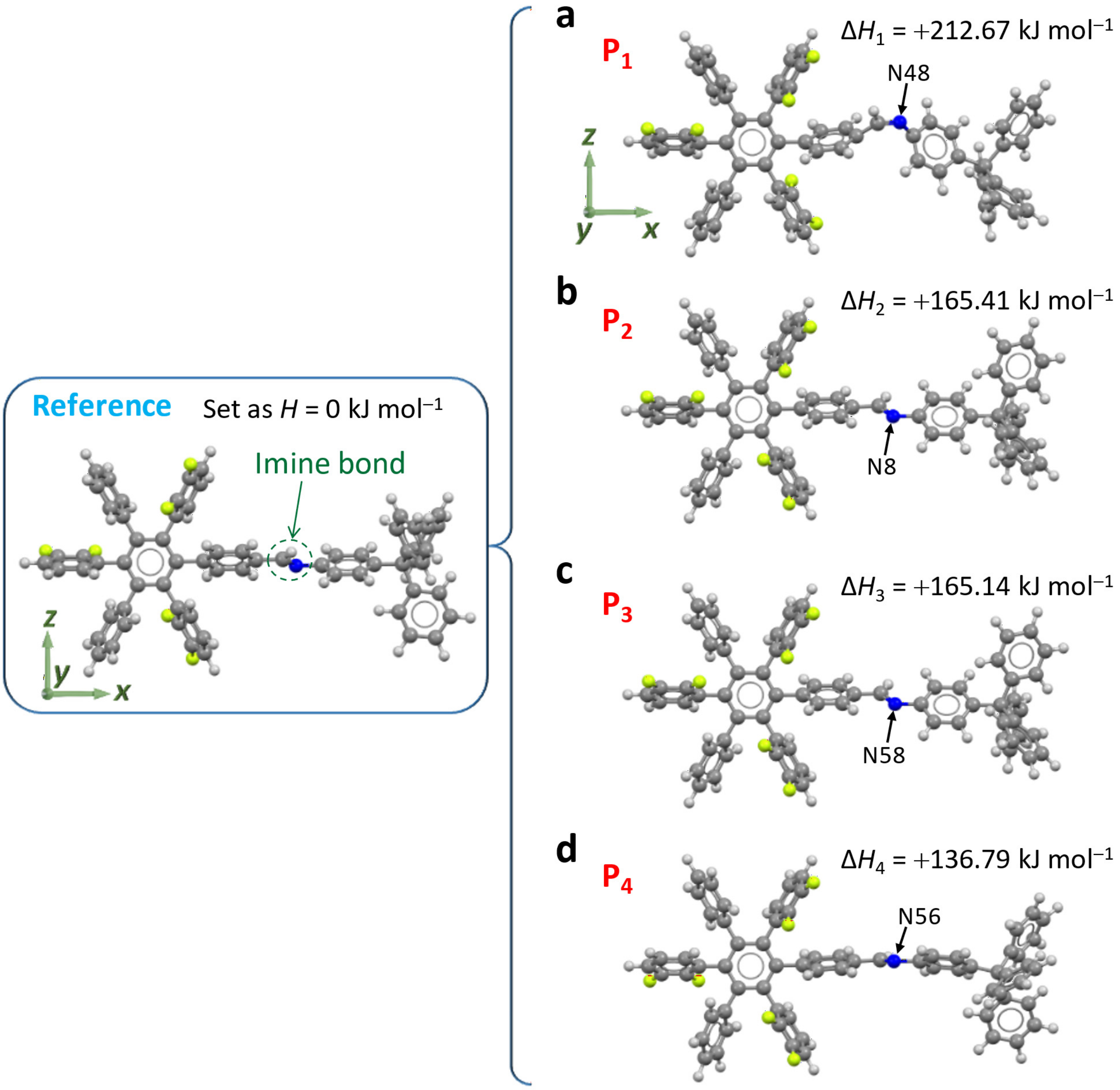

Figure S36. (a–d) The structures of the moieties $\mathbf{P}_n$ for **TK-COF-P** and their energies ΔH_n for n = 1 to 4, respectively. The coordinates are common to all graphics. For the label numbers of the nitrogen atoms in the imine bonds, refer to Table S6.

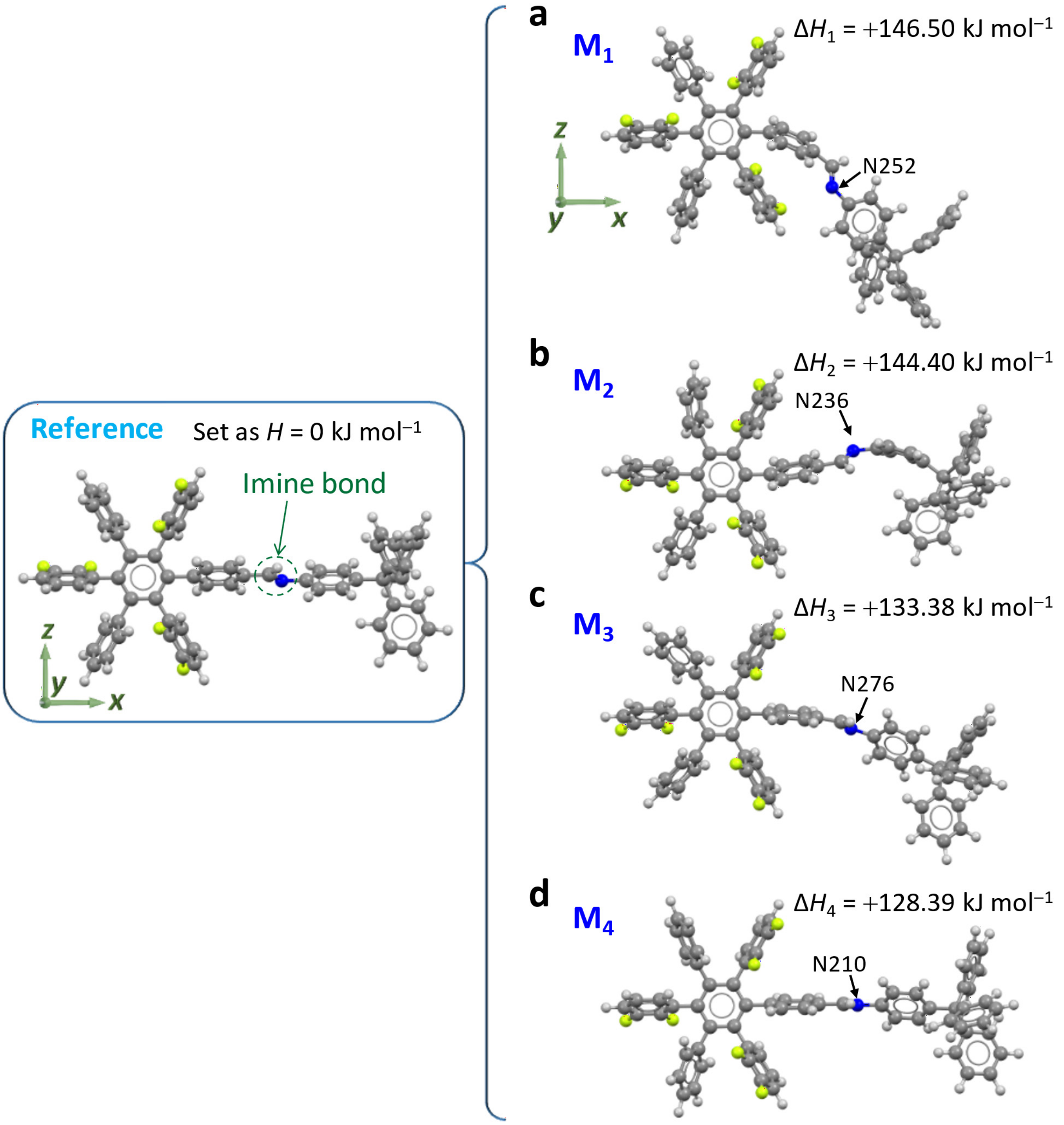

Figure S37. (a–d) The structures of the moieties $\mathbf{M}_n$ for **TK-COF-M** and their energies ΔH_n for $n = 1$ to 4, respectively. The coordinates are common to all graphics. For the label numbers of the nitrogen atoms in the imine bonds, refer to Table S7.

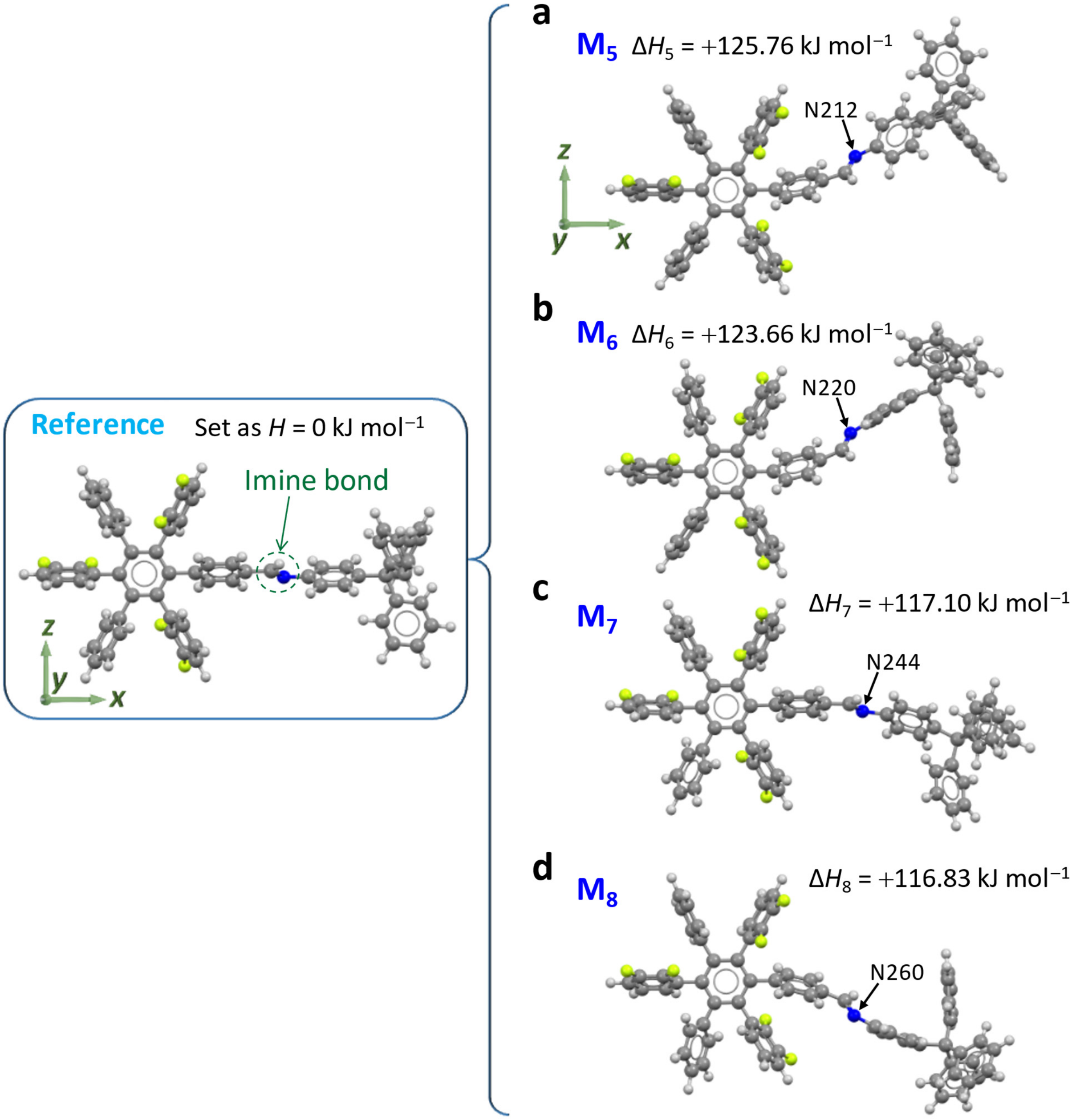

Figure S38. (a–d) The structures of the moieties $\mathbf{M}_n$ for **TK-COF-M** and their energies ΔH_n for $n = 5$ to 8, respectively. The coordinates are common to all graphics. For the label numbers of the nitrogen atoms in the imine bonds, refer to Table S7.

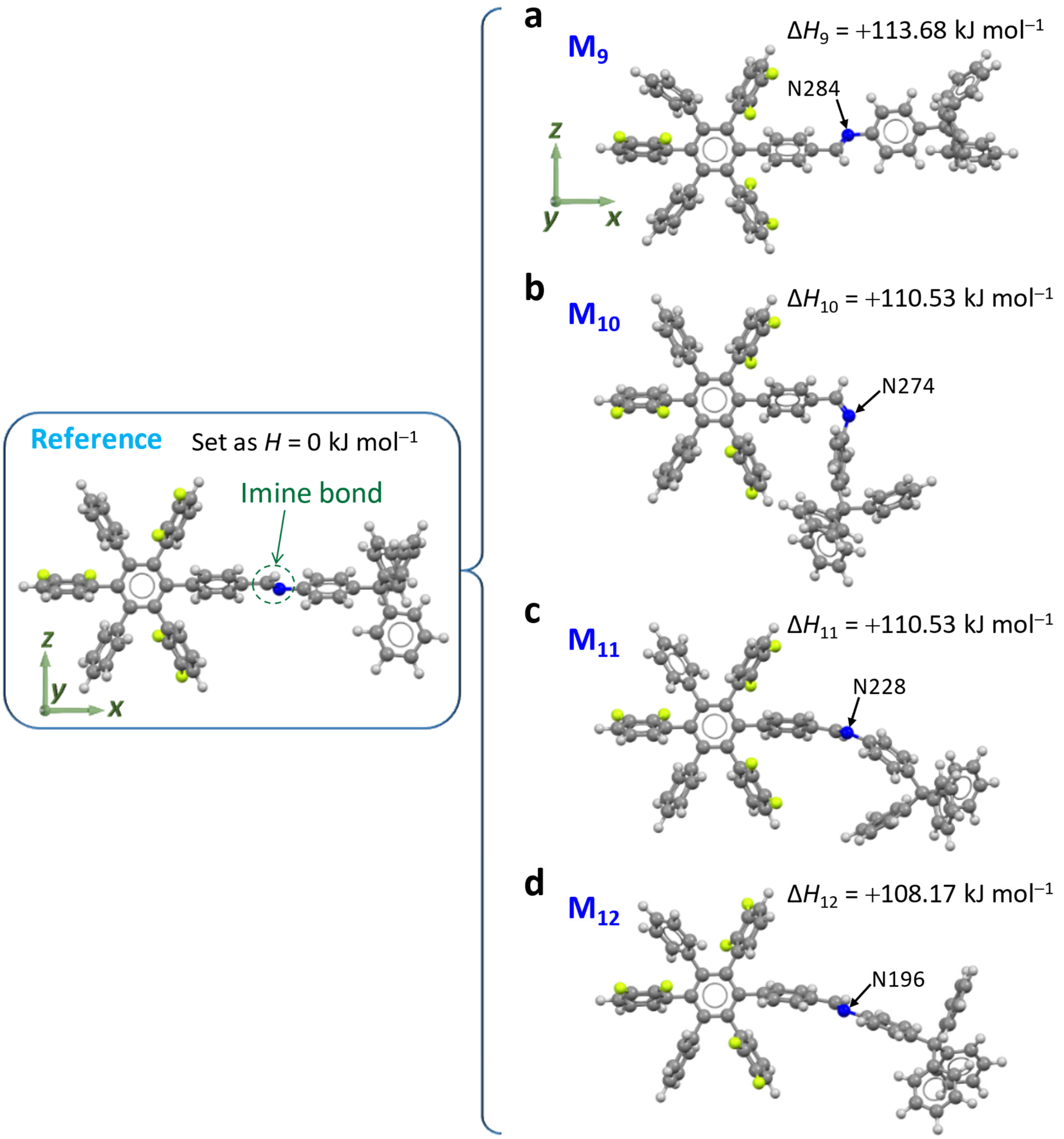

Figure S39. (a–d) The structures of the moieties $\mathbf{M}_n$ for **TK-COF-M** and their energies ΔH_n for n = 9 to 12, respectively. The coordinates are common to all graphics. For the label numbers of the nitrogen atoms in the imine bonds, refer to Table S7.

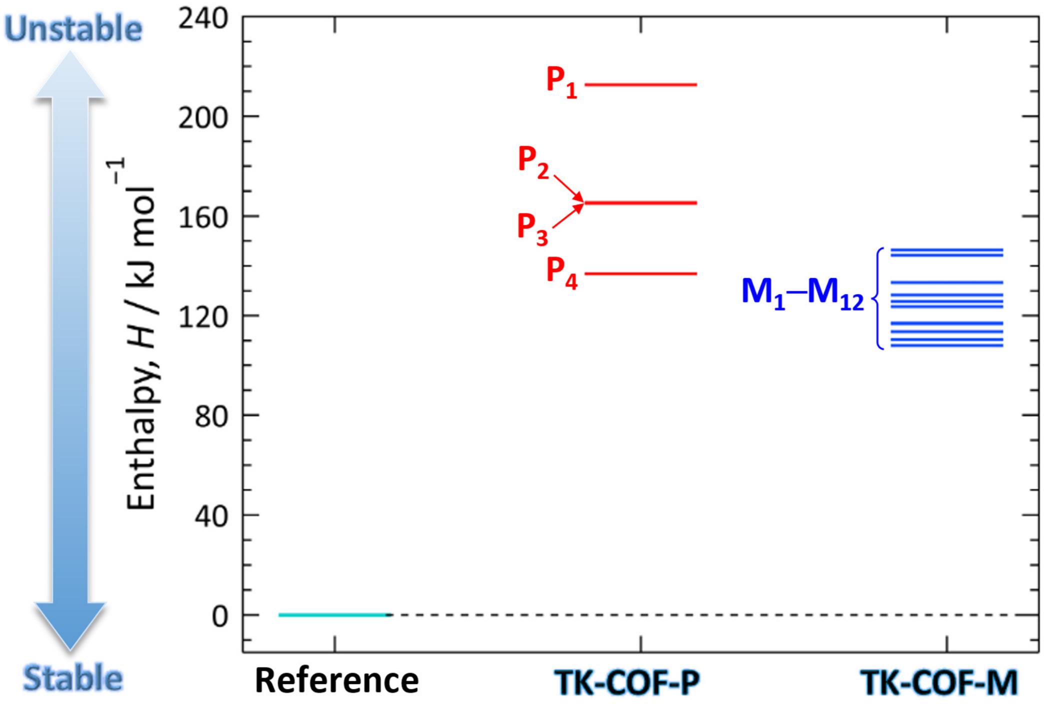

Figure S40. Summary of the energy calculations for the structural moieties $\mathbf{P}_n$ for **TK-COF-P** (n = 1 to 4; Figure S36) and the structural moieties $\mathbf{M}_n$ moieties (n = 1 to 12; Figures S37–S39) in which the energy of the reference structural moiety (Figure S35) has been set to H = 0. All calculations were carried out using Gaussian® software at the ωB97XD/6-31G+(d,p) level of theory.

S5.3 Total energies of **TK-COF-P** and **-M**

We calculated the total energies of **TK-COF-P** and **-M** using the universal force field in Materials Studio® software. Total energy calculations were carried out with the chemical formula $C_{85}H_{48}N_4F_8$ for both **TK-COF-P** and **-M**. As shown in Table S9, the total energy of **TK-COF-P** is greater than that of **TK-COF-M**, indicating that **TK-COF-P** is more energetically unstable than **TK-COF-M**. This result is consistent with the experimental results that **TK-COF-P** is a kinetic isomer.

Table S9. Calculated total energies of **TK-COF-P** and **-M**.

	**TK-COF-P** / kJ mol^{-1}	**TK-COF-M** / kJ mol^{-1}
Valence energy (diag. terms): {A}	1280.3	731.8
Bond	430.8	158.1
Angle	385.5	57.6
Torsion	458.3	510.5
Inversion	5.7	5.5
Non-bond energy: {B}	525.8	576.8
van der Waals	526.6	577.9
Long range correction	–0.9	–1.1
Electrostatic	0	0
Total energy = {A} + {B}	1806.1	1308.6

S5.4 Explanation of the evolution into different morphologies depending on the solvent ratio

As shown above, the product with a prism shape (**TK-COF-P**) is kinetically controlled (*i.e.*, quasi-stable), and the product with a membrane shape (**TK-COF-M**) is thermodynamically stable. We consider that our choice of different dioxane:mesitylene ratios, 4:1 or 1:1, essentially affects the reaction coordinate to evolve the kinetic product (**TK-COF-P**) or thermodynamic product (**TK-COF-M**).

Notably, dioxane often gives high solubilities to the building blocks used for COFs. In fact, both **HABF** and **TAM**, the building blocks used in this report, were highly soluble (*i.e.*, stabilized) in dioxane, whereas they were insoluble (*i.e.*, destabilized) in mesitylene. Importantly, mesitylene has been known to energetically stabilize aromatic frameworks.[S9,S10] Based on these facts, we propose that one of the reasons why the dioxane/mesitylene mixed solvent has often been used for the growth of COFs is that, by choosing their ratio, *one can conveniently tune the relative magnitudes of the chemical potentials of the reactant monomers and the product COFs, because dioxane lowers the chemical potential of reactants whereas mesitylene lowers (raises) the chemical potential of products (reactants).*

Therefore, when we used the dioxane-rich "dioxane:mesitylene = 4:1" solvent, we obtained the kinetic product (**TK-COF-P**); when we used the mesitylene-rich "dioxane:mesitylene = 1:1" solvent, we obtained the thermodynamic product (**TK-COF-M**). To summarize, our experimental results can be explainable in terms of the qualitative energy diagram shown below.

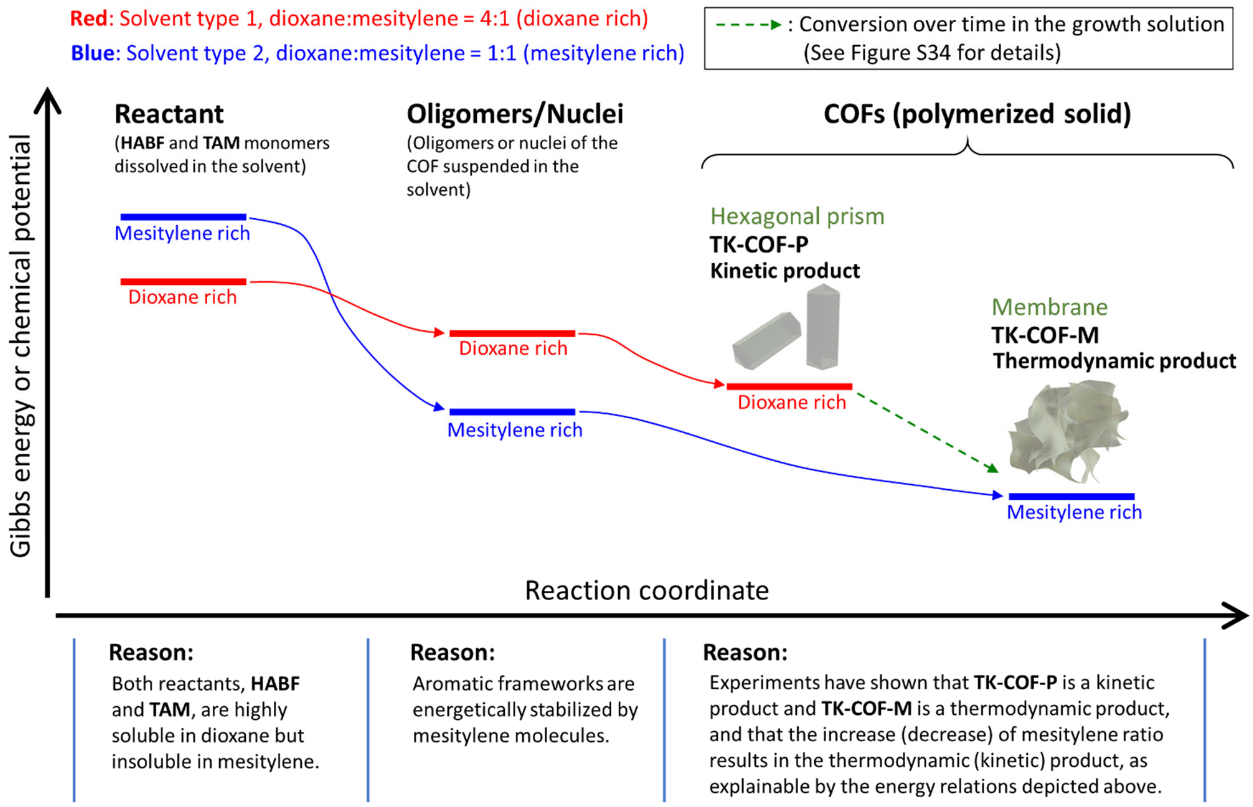

Figure S41. Qualitative energy diagram to explain the reason for the observed evolutions into different morphologies (**TK-COF-P** *vs*. **TK-COF-M**) depending on the dioxane:mesitylene ratio.

S5.5 SEM images showing membranes grown from the side of prisms

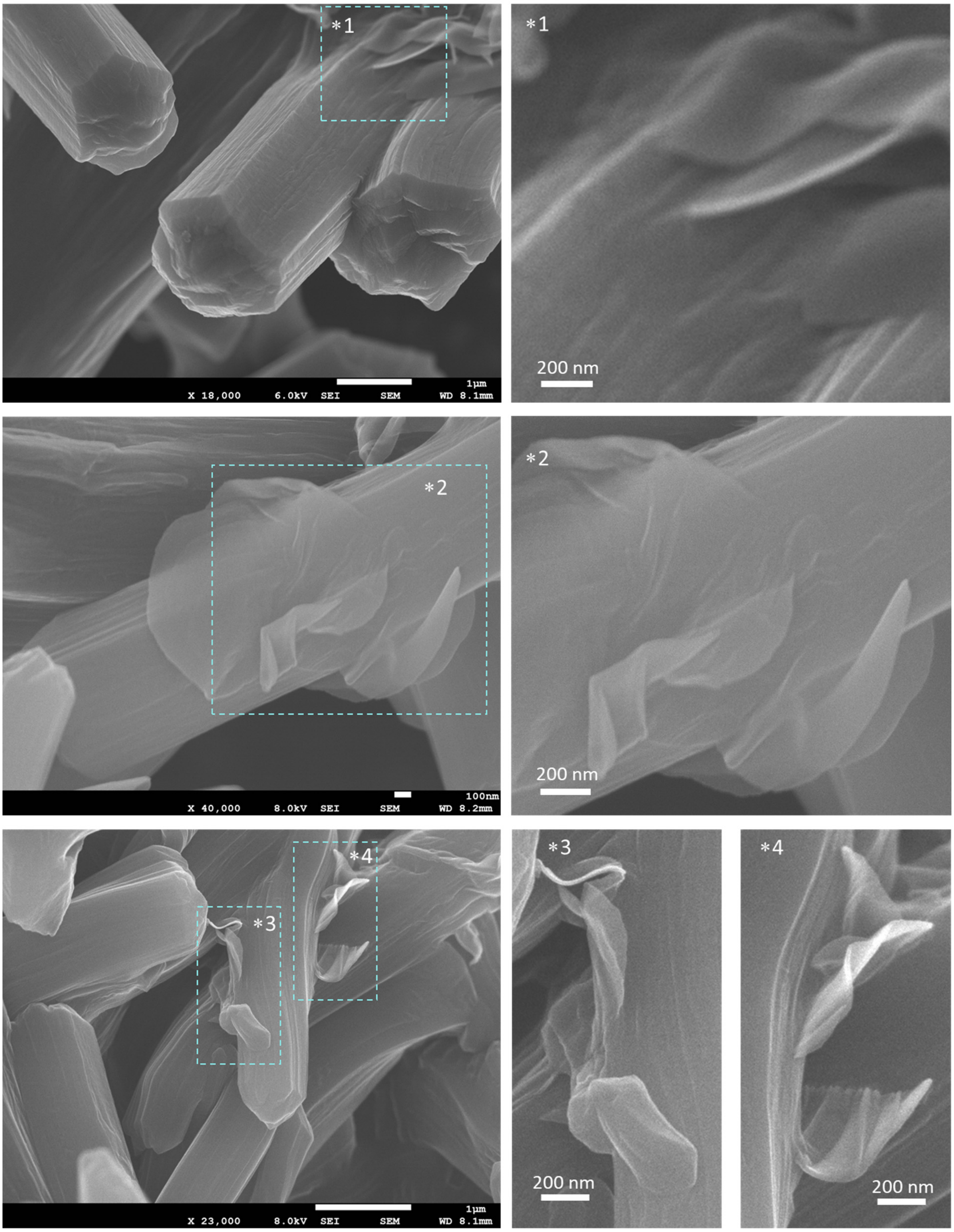

Figure S42. SEM images showing membranes growing from the side of a hexagonal prism or **TK-COF-P**.

S5.6 Comparison of non-bond energies of **TK-COF-P/-M** and those of hypothetical H-substituted **TK-COF-P/-M**

To assess the significance of the hydrogen-bonding interactions between the F atoms on the rotors and adjacent H atoms, we compared the non-bond energies, which are responsible for hydrogen bonds, of **TK-COF-P** and **-M** with those of the corresponding hypothetical COFs in which F atoms in **TK-COF-P** and **-M** are replaced with H atoms; they are denoted **K-COF-P**(F→H) and **TK-COF-M**(F→H), respectively. We calculated the energies using the universal force field with Materials Studio® software, as we did in Section S5.3 above.

The results are compared in Tables S10 and S11. As the results show, the differences in the non-bond energies were less than 2 kJ mol^{-1}, where mol is defined based on the number of rotors. This energy difference is comparable to the thermal energy at room temperature (*ca.* 2.5 kJ mol^{-1}), and hence the hydrogen-bond interactions between the F and H atoms in these COFs are not considered to be significant, at least to the extent that such interactions affect the motion of the rotors.

Table S10. Comparison of non-bond energies of **TK-COF-P** and **TK-COF-P**(F→H).

	**TK-COF-P**^{a,b} / kJ mol^{-1}	**TK-COF-P**(F→H)^{b} / kJ mol^{-1}	Difference^{b} / kJ mol^{-1}	Difference^{c} / kJ mol^{-1}
Non-bond energy: ($= x + y + z$)	525.76	518.50	−7.26	−1.82
van der Waals (x)	526.64	519.94	−6.69	−1.67
Long range correction (y)	−0.88	−1.45	−0.57	−0.14
Electrostatic (z)	0.00	0.00	0.00	0.00

^{a} Identical to the calculation results shown in Table S9.
^{b} Per mole of the chemical formula: $C_{85}H_{48}N_4F_8$ for **TK-COF-P** and $C_{85}H_{56}N_4$ for **TK-COF-P**(F→H).
^{c} Per mole of the number of rotors.

Table S11. Comparison of non-bond energies of **TK-COF-M** and **TK-COF-M**(F→H).

	**TK-COF-M**[a,b] / kJ mol^{-1}	**TK-COF-M**(F→H)[b] / kJ mol^{-1}	Difference[b] / kJ mol^{-1}	Difference[c] / kJ mol^{-1}
Non-bond energy: ($= x + y + z$)	576.79	570.19	−6.60	−1.65
van der Waals (x)	577.92	572.06	−5.86	−1.46
Long range correction (y)	−1.13	−1.87	−0.74	−0.18
Electrostatic (z)	0.00	0.00	0.00	0.00

[a] Identical to the calculation results shown in Table S9.
[b] Per mole of the chemical formula: $C_{85}H_{48}N_4F_8$ for **TK-COF-M** and $C_{85}H_{56}N_4$ for **TK-COF-M**(F→H).
[c] Per mole of the number of rotors.

Section S6. Electric modulus analysis and results

S6.1 Formulation of electric modulus analysis

As has been shown previously,[S11,S12] dielectric relaxation can be studied using the dielectric modulus representation even in the absence of a well-defined peak in ε''. The electric modulus (M) corresponds to the relaxation of the electric field in the materials when the electric displacement remains constant and is given by the formulation of[S11]

$$M = M' + iM'' , \tag{S3}$$

$$M = \frac{1}{\varepsilon} = \frac{1}{\varepsilon' + i\varepsilon''} , \tag{S4}$$

$$M' = \frac{\varepsilon'}{\varepsilon'^2 + \varepsilon''^2} , \; M'' = \frac{\varepsilon''}{\varepsilon'^2 + \varepsilon''^2} . \tag{S5}$$

Using these equations, the dataset of ε' and ε'' can be converted to that of M' and M''. The frequency (f_m) at which the plot of M'' *vs*. frequency gives the maximum is related to the characteristic relaxation time by $\tau = (2\pi f_m)^{-1}$.[S11,S12] Therefore, once the temperature dependence of f_m is obtained, we can use the Arrhenius relation

$$\tau^{-1} = \tau_0{}^{-1} \exp\left(-\frac{E_a}{RT}\right) , \tag{S6}$$

where E_a (J mol^{-1}) is the activation energy, R (J mol^{-1} K^{-1}) is the gas constant, and T (K) is the temperature.

S6.2 Temperature and frequency dependences of M' and M'' for **TK-COF-M**

Dependence of M' on the angular frequency ω (= $2\pi f$) and temperature is plotted in Figure S43 below. See Figure 3f in the main text for the plot for M''.

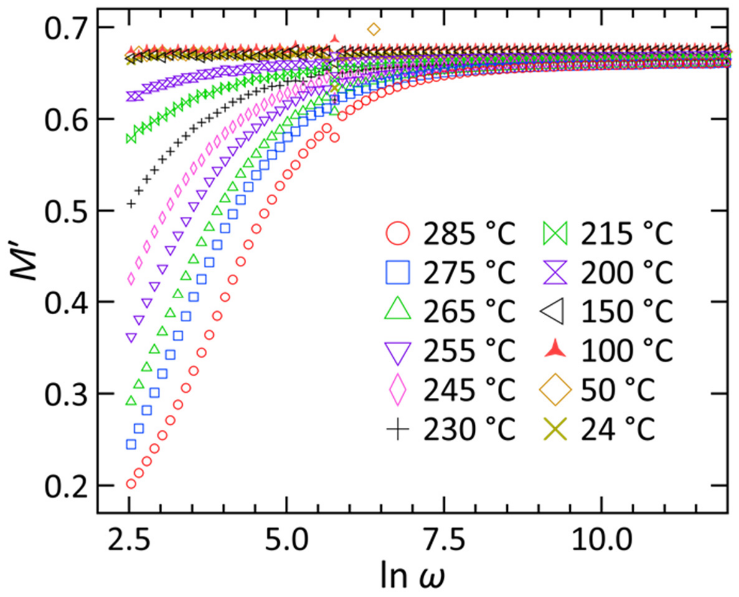

Figure S43. Dependence of M' on the angular frequency and temperature calculated from the dielectric response data for **TK-COF-M**.

The values of M' and M'' can also be plotted against temperature for constant frequency as shown in Figures S44a and S44b, respectively. By using the relationship between the temperature that gives the maximum in the M'' plot (T_m) and the frequency corresponding to the plot (f), along with a relationship of $\tau = (2\pi f)^{-1}$, an Arrhenius plot can be made.[S12] This type of Arrhenius plot is essentially the same as the Arrhenius plot in the inset of Figure 3f in the main text and hence gives the same E_a value.

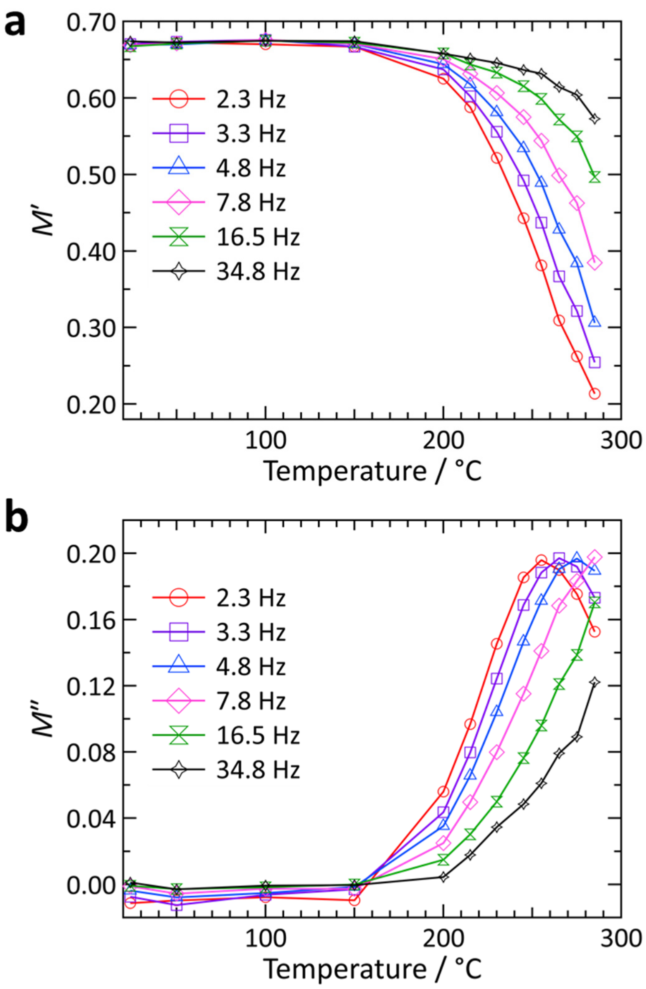

Figure S44. Plots of (a) M' and (b) M'' against temperature for constant frequency, calculated from the dielectric response data for **TK-COF-M**.

S6.3 Temperature and frequency dependences of M' and M'' for **TK-COF-P**

Dependences of M' and M'' on ω and temperature are plotted in Figures S45a and S45b below, respectively. The plots of M' and M'' against temperature for a constant frequency are shown in Figures S46a and S46b, respectively.

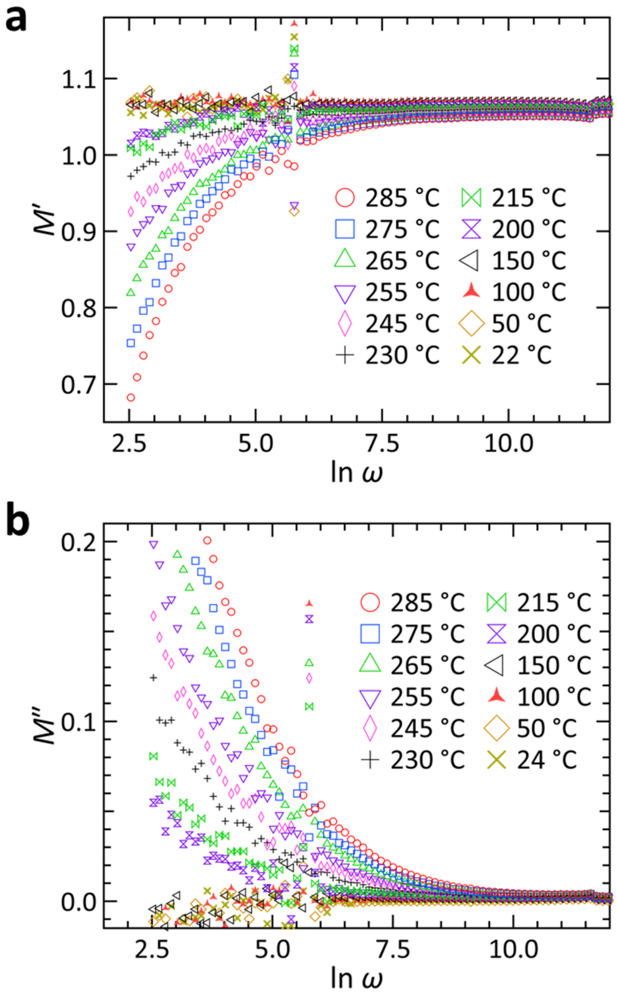

Figure S45. Dependences of M' and M'' on the angular frequency and temperature calculated from the dielectric response data for **TK-COF-P**.

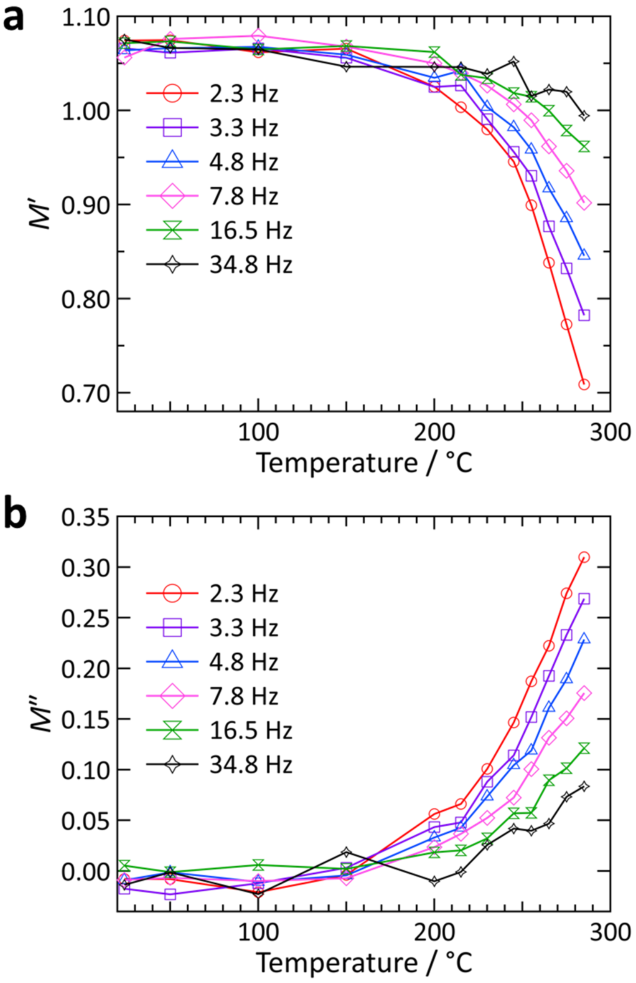

Figure S46. Plots of (a) *M′* and (b) *M″* against temperature for constant frequency, calculated from the dielectric response data for **TK-COF-P**.

Section S7. Dipolar rotor dynamics investigated by temperature-dependent solid-state ^{19}F NMR measurements

S7.1 Measurement methods and samples

Temperature-dependent ^{19}F ss-NMR measurements were carried out using a Bruker Avance III HD wide-bore 300 MHz (300WB) NMR spectrometer equipped with a 4-mm double-resonance wide-range variable temperature (WVT) MAS probe. For temperature-dependent experiments, the sample was packed in a 4-mm ZrO_2 rotor with a ZrO_2 cap. The MAS rate was set at 10 kHz. This WVT probe utilizes the bearing gas to control the sample temperature. The temperature of the bearing gas (T_{gas}) can be raised up to 300 ºC; raising T_{gas} above 300 °C is prohibited by the NMR system for safety reason. The exact temperature at the sample position (T_{sample}) for each T_{gas} was carefully calibrated using the ^{79}Br chemical shift values from a KBr powder sample, which depend linearly on temperature.[S13]

The upper temperature limit of the sample allowed in this NMR system was strictly 285 °C, because when T_{sample} was 285 °C, T_{gas} was 297 °C, which is very close to the upper limit of T_{gas} = 300 °C mentioned above. This is why the measurement for the high temperature side had ended at 285 °C in Figure 3g in the main text.

All COF samples were packed with KBr pellets as a spacer to locate the samples at the center of the rotor in a glovebox equipped with a circulation gas purifier (VAC, OMNI-LAB). Prior to use, KBr powder was ground using a quartz mortar, dried under vacuum in an electric oven overnight, and stored in the glovebox. During NMR measurements, the sample tube was spun and heated by dried nitrogen gas that passed through a nitrogen separation membrane module.

1D ^{19}F spectra (Larmor frequency: 282.40 MHz) were recorded with π/2- and π-pulses, employing the Hahn echo method to suppress background signals from probe materials.[S14] ^{19}F T_1

(spin-lattice relaxation time) values were determined using a conventional saturation-recovery method[S15] followed by a Hahn echo sequence, for which the signals were accumulated in 16-scan increments for each recovery time. ^{19}F $T_{1\rho}$ (spin-lattice relaxation time in the rotating frame) values were determined using the spin-lock method with 180-W irradiation for up to 50 ms, corresponding to a spin-lock frequency of *ca*. 65 kHz for **TK-COF-M** and *ca*. 80 kHz for **TK-COF-P**. The repetition time used was *ca*. 1.3 times the ^{19}F T_1 value under each temperature to utilize the highest repetition efficiency. The ^{19}F chemical shifts were referenced to the polytetrafluoroethylene (PTFE) signal at −122 ppm[S16] (relative to $CFCl_3$ at 0 ppm).

S7.2 Raw data from ^{19}F spin-lattice relaxation measurements for T_1 and $T_{1\rho}$

Figures S47 and S48 (S49 and S50) show the data from T_1 and $T_{1\rho}$ relaxation measurements, respectively, acquired by ^{19}F solid-state NMR for **TK-COF-M** (**TK-COF-P**) at different temperatures. The T_1 relaxation data were well fit with a single exponential function

$$I(t) = I_0\left(1 - e^{-t/\tau}\right), \tag{S7}$$

where $\tau = T_1$. The data for the temperature dependence of T_1 for **TK-COF-M** and **TK-COF-P** are shown in Figures S52a and S52b, respectively.

The $T_{1\rho}$ relaxation data were well fit with the following double-exponential function

$$I(t) = I_{0,\text{fast}}e^{-t/\tau_\text{fast}} + I_{0,\text{slow}}e^{-t/\tau_\text{slow}}, \tag{S8}$$

where $I_{0,\text{fast}}$ and $I_{0,\text{slow}}$ denote the amplitudes of the signal and τ_fast and τ_slow denote the decay time constants of the respective components. We also introduce the intensity fractions A_fast and A_slow as follows.

$$A_\text{fast} \equiv \frac{I_{0,\text{fast}}}{I_{0,\text{fast}} + I_{0,\text{slow}}}, \quad A_\text{slow} \equiv \frac{I_{0,\text{slow}}}{I_{0,\text{fast}} + I_{0,\text{slow}}} \quad (A_\text{fast} + A_\text{slow} = 1). \tag{S9}$$

We plotted the temperature dependence of τ_fast and τ_slow for **TK-COF-M** (**TK-COF-P**) in Figure S53a (Figure S53c) and the temperature dependence of A_fast and A_slow in Figure S53b (Figure S53d). As shown therein, A_slow was the dominant signal occupying approximately 80% of the total signal. Because τ_slow decreased with an increase in temperature (*i.e.*, physically reasonable behavior), whereas τ_fast was insensitive to the temperature, we have regarded τ_slow as $T_{1\rho}$. The fast component is considered to originate from the residual ^{19}F background because of the incomplete suppression of the background.

Based on this assignment, the data for the temperature dependence of $T_{1\rho}$ for **TK-COF-M** and **TK-COF-P** are shown in Figures S54a and S54b, respectively. Additionally, we measured the T_1

and $T_{1\rho}$ relaxations for the molecular solid of **HABF** at 34 °C (Figure S51) to understand the difference between the relaxation times of **TK-COF-M/-P** and the molecular solid of **HABF**, the results of which have been compared in Figure 3h in the main text.

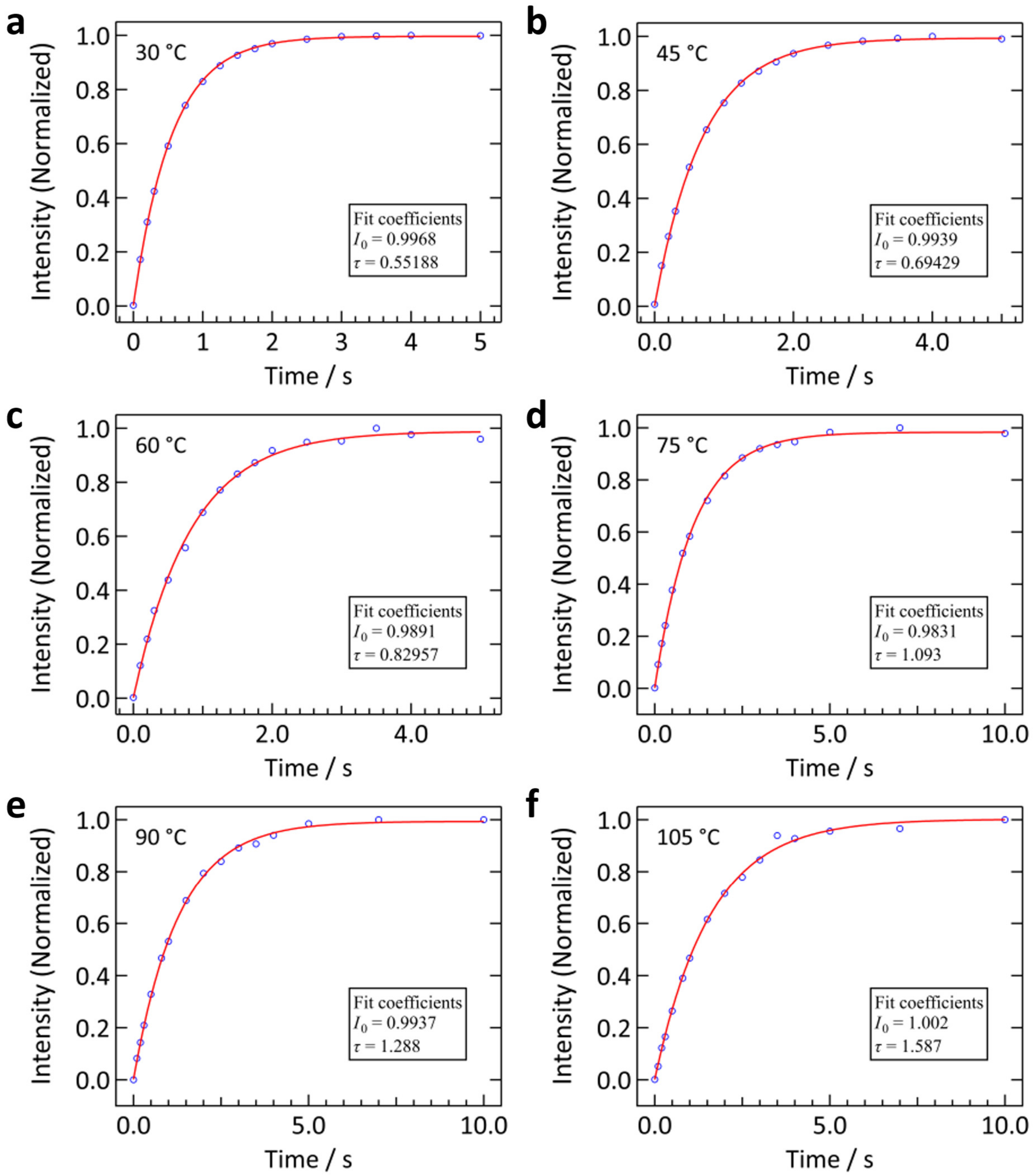

Figure S47. T_1 relaxation measured by ^{19}F solid-state NMR for **TK-COF-M** at different temperatures and the curve fit by equation S7. The temperature is shown in each panel. See Figure S52a for the temperature dependence of T_1.

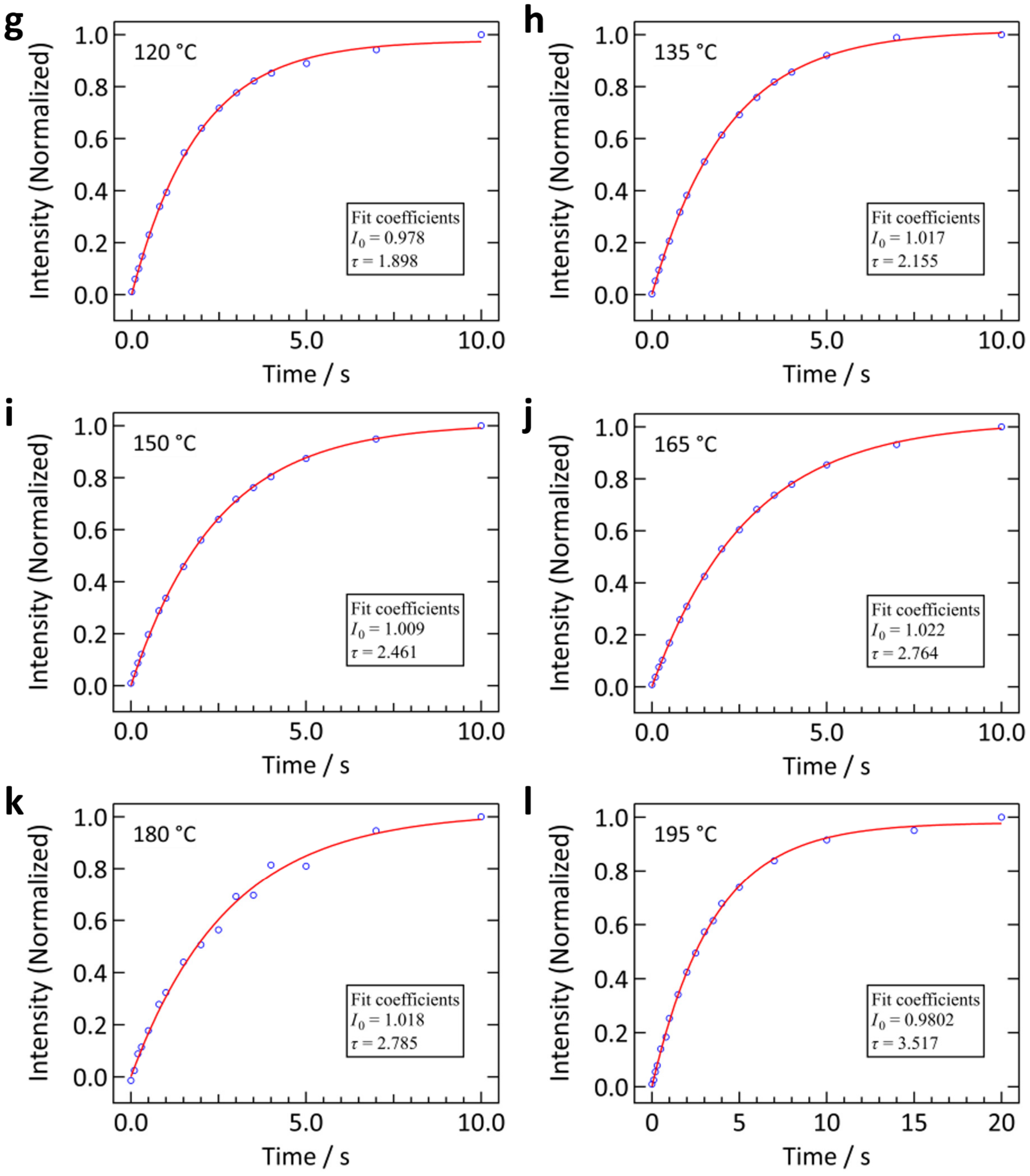

Figure S47 (continued). T_1 relaxation measured by ^{19}F solid-state NMR for **TK-COF-M** at different temperatures and the curve fit by equation S7. The temperature is shown in each panel. See Figure S52a for the temperature dependence of T_1.

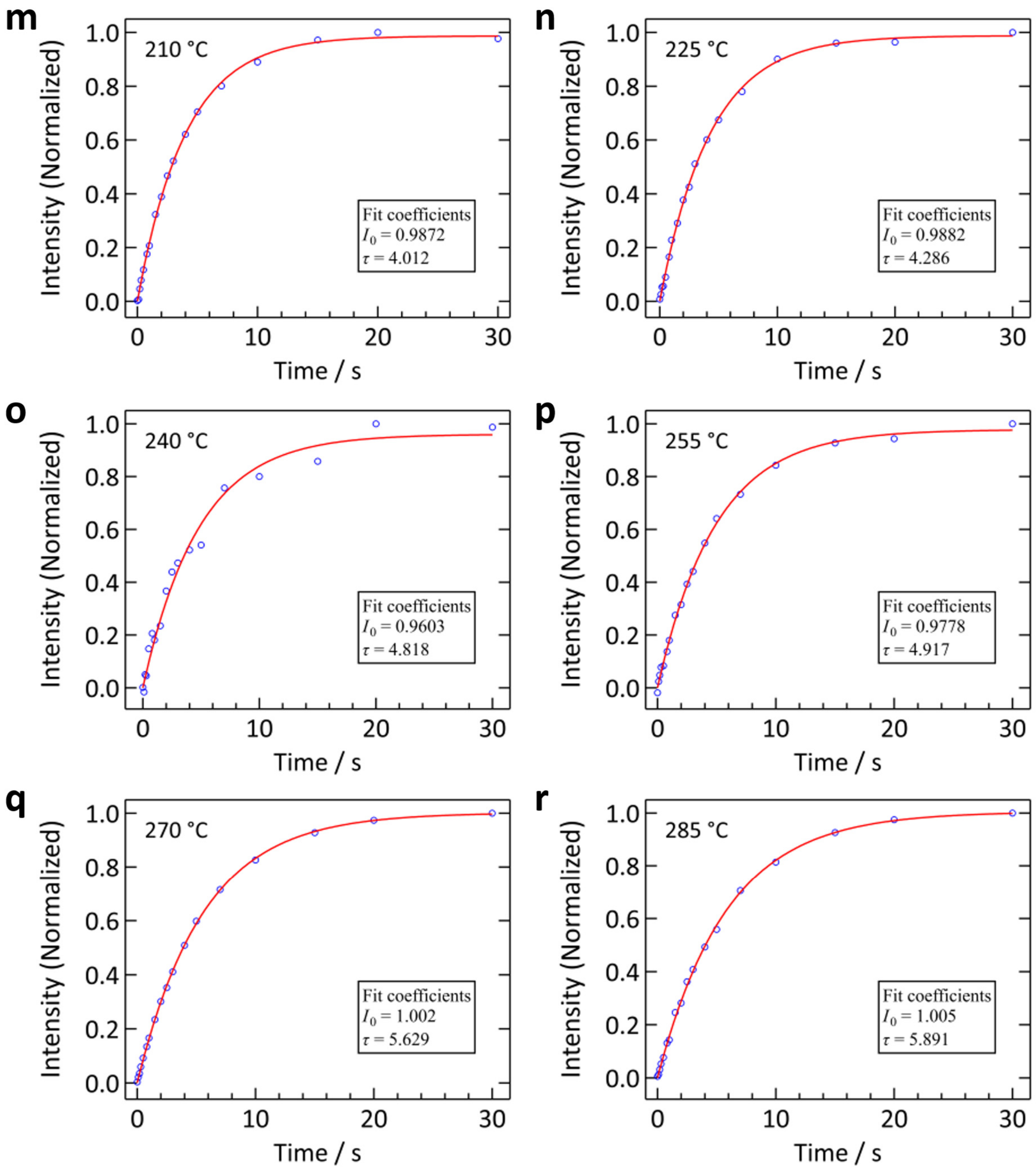

Figure S47 (continued). T_1 relaxation measured by ^{19}F solid-state NMR for **TK-COF-M** at different temperatures and the curve fit by equation S7. The temperature is shown in each panel. See Figure S52a for the temperature dependence of T_1.

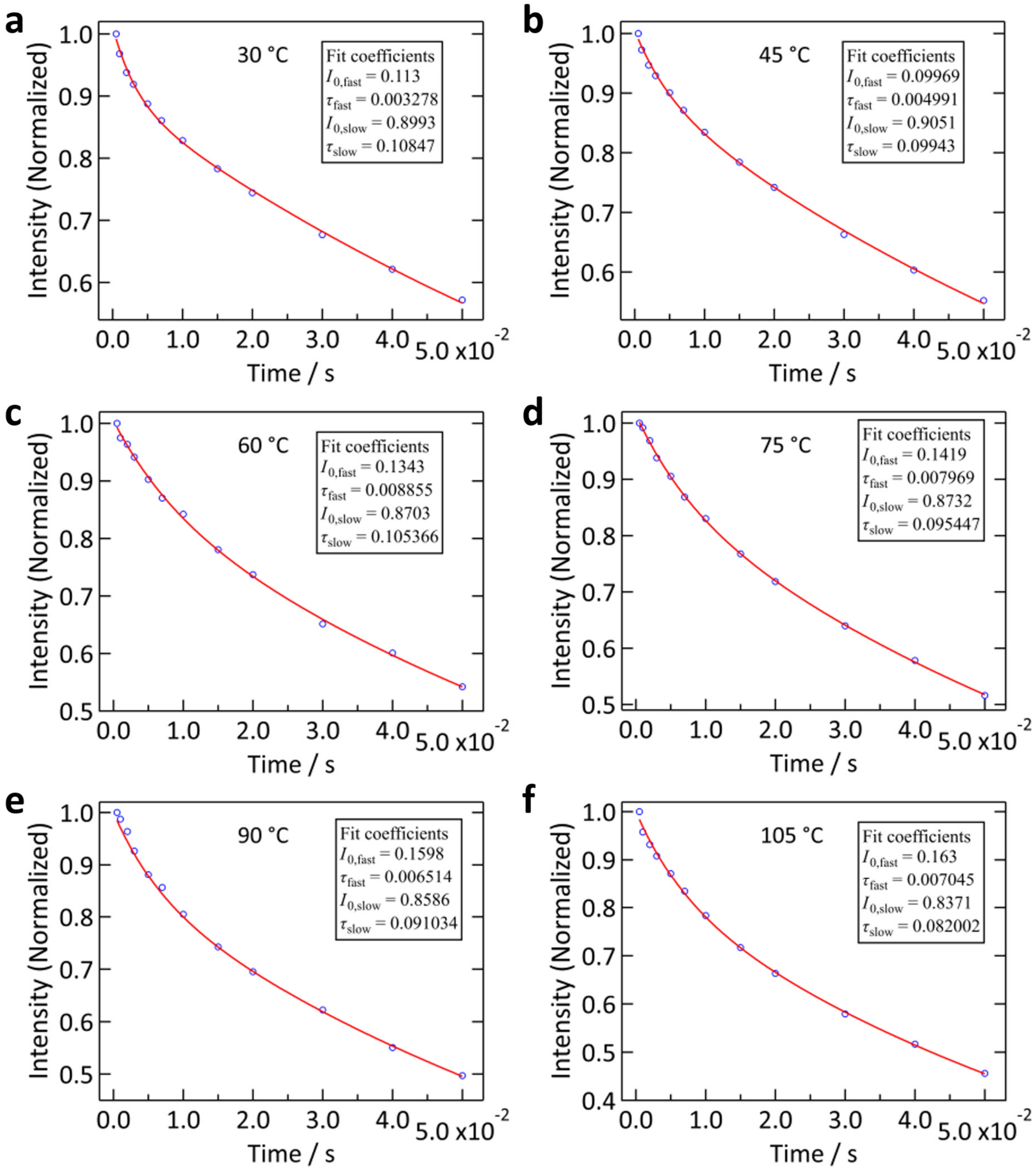

Figure S48. $T_{1\rho}$ relaxation measured by ^{19}F solid-state NMR for **TK-COF-M** at different temperatures and the curve fit by equation S8. The temperature is shown in each panel. See Figure S54a for the temperature dependence of $T_{1\rho}$.

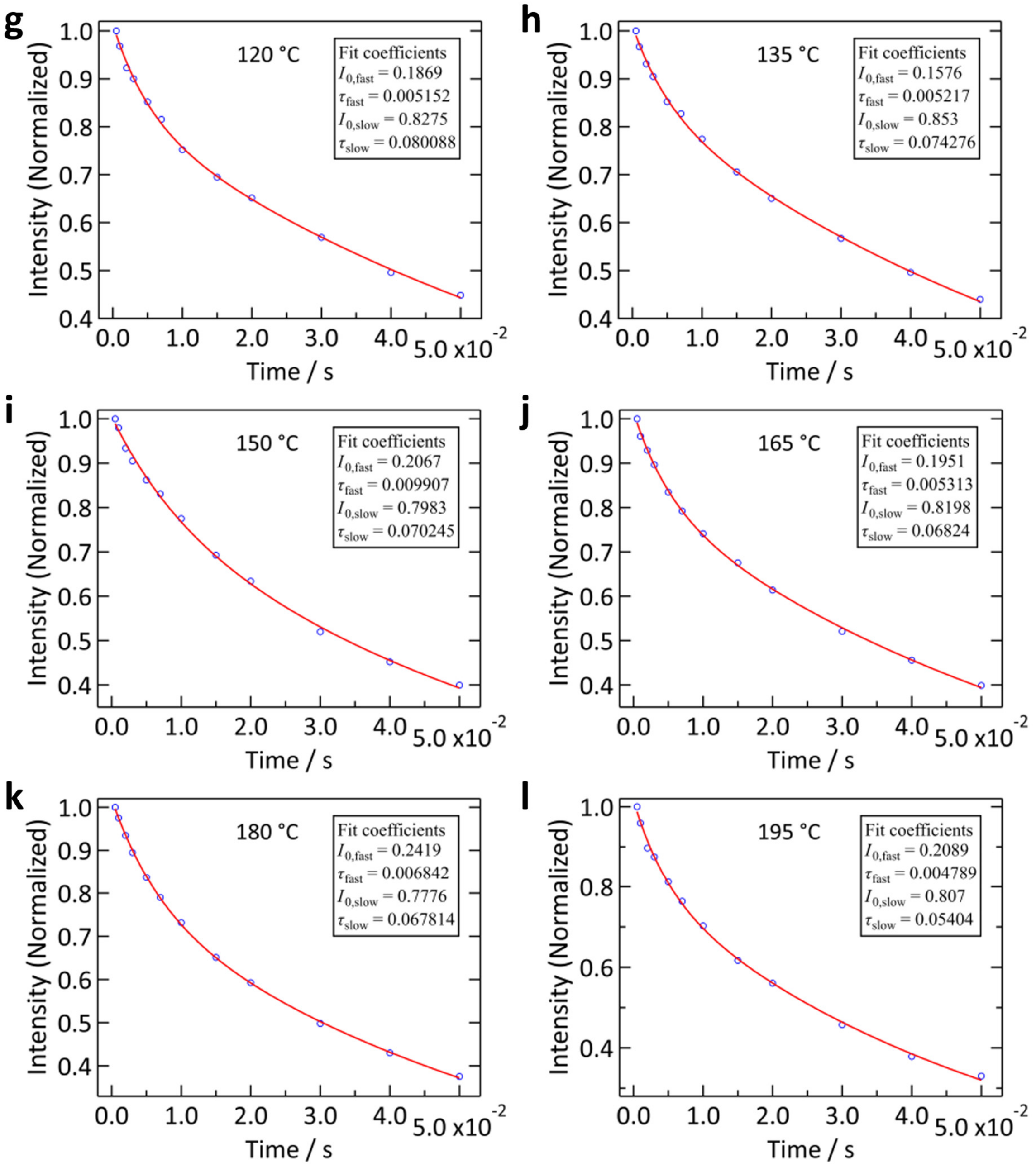

Figure S48 (continued). $T_{1\rho}$ relaxation measured by ^{19}F solid-state NMR for **TK-COF-M** at different temperatures and the curve fit by equation S8. The temperature is shown in each panel. See Figure S54a for the temperature dependence of $T_{1\rho}$.

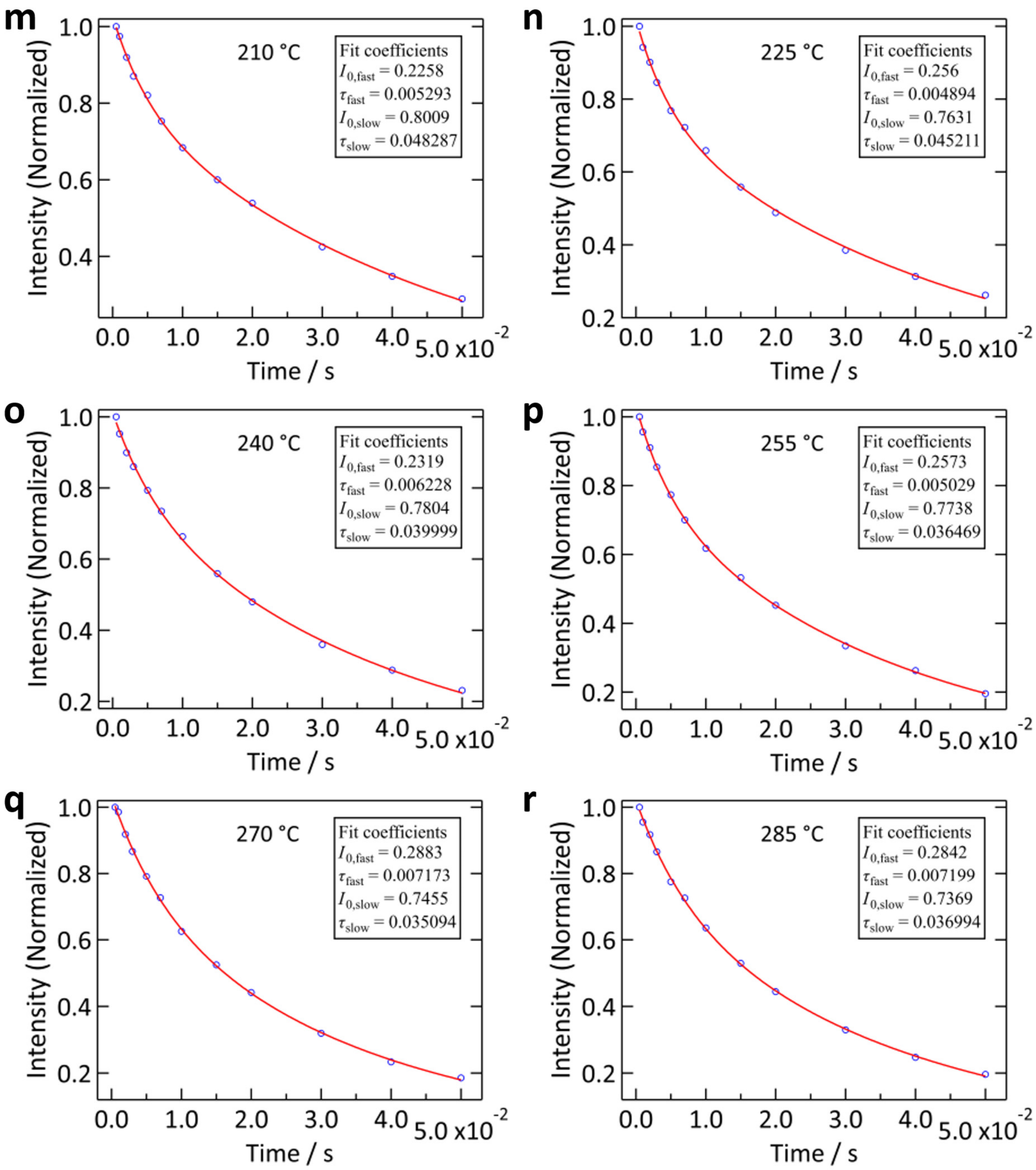

Figure S48 (continued). $T_{1\rho}$ relaxation measured by ^{19}F solid-state NMR for **TK-COF-M** at different temperatures and the curve fit by equation S8. The temperature is shown in each panel. See Figure S54a for the temperature dependence of $T_{1\rho}$.

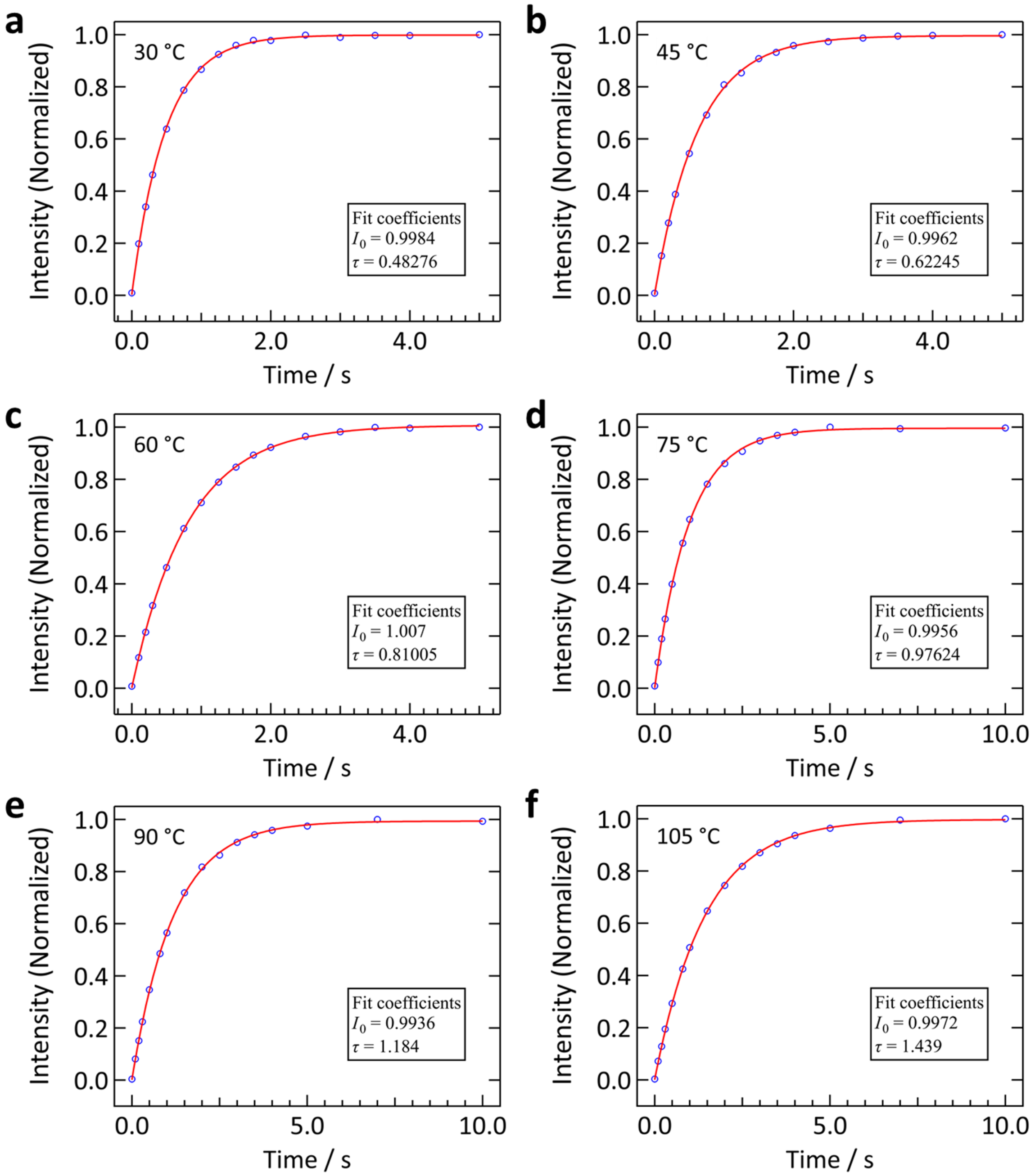

Figure S49. T_1 relaxation measured by ^{19}F solid-state NMR for **TK-COF-P** at different temperatures and the curve fit by equation S7. The temperature is shown in each panel. See Figure S52b for the temperature dependence of T_1.

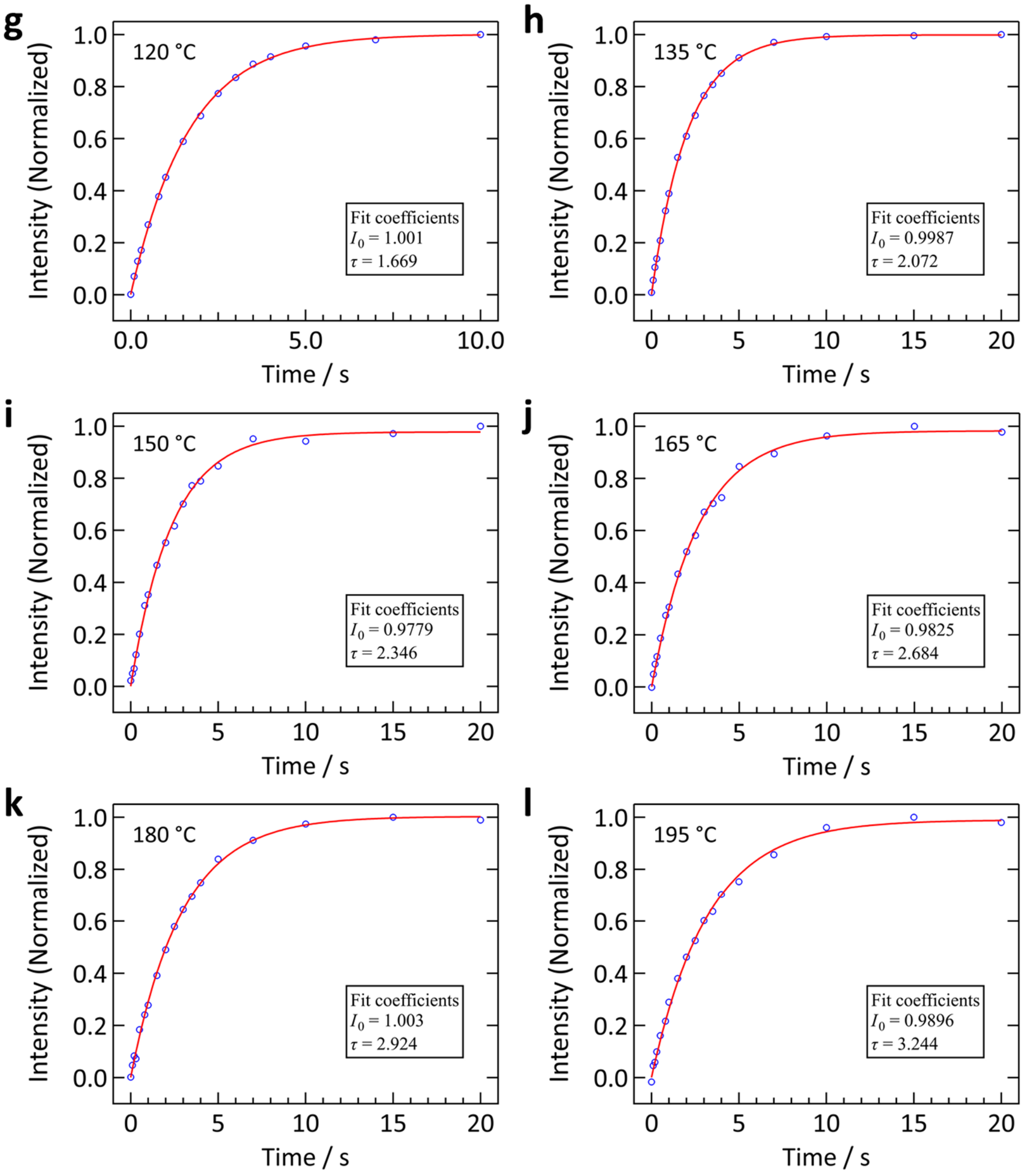

Figure S49 (continued). T_1 relaxation measured by ^{19}F solid-state NMR for **TK-COF-P** at different temperatures and the curve fit by equation S7. The temperature is shown in each panel. See Figure S52b for the temperature dependence of T_1.

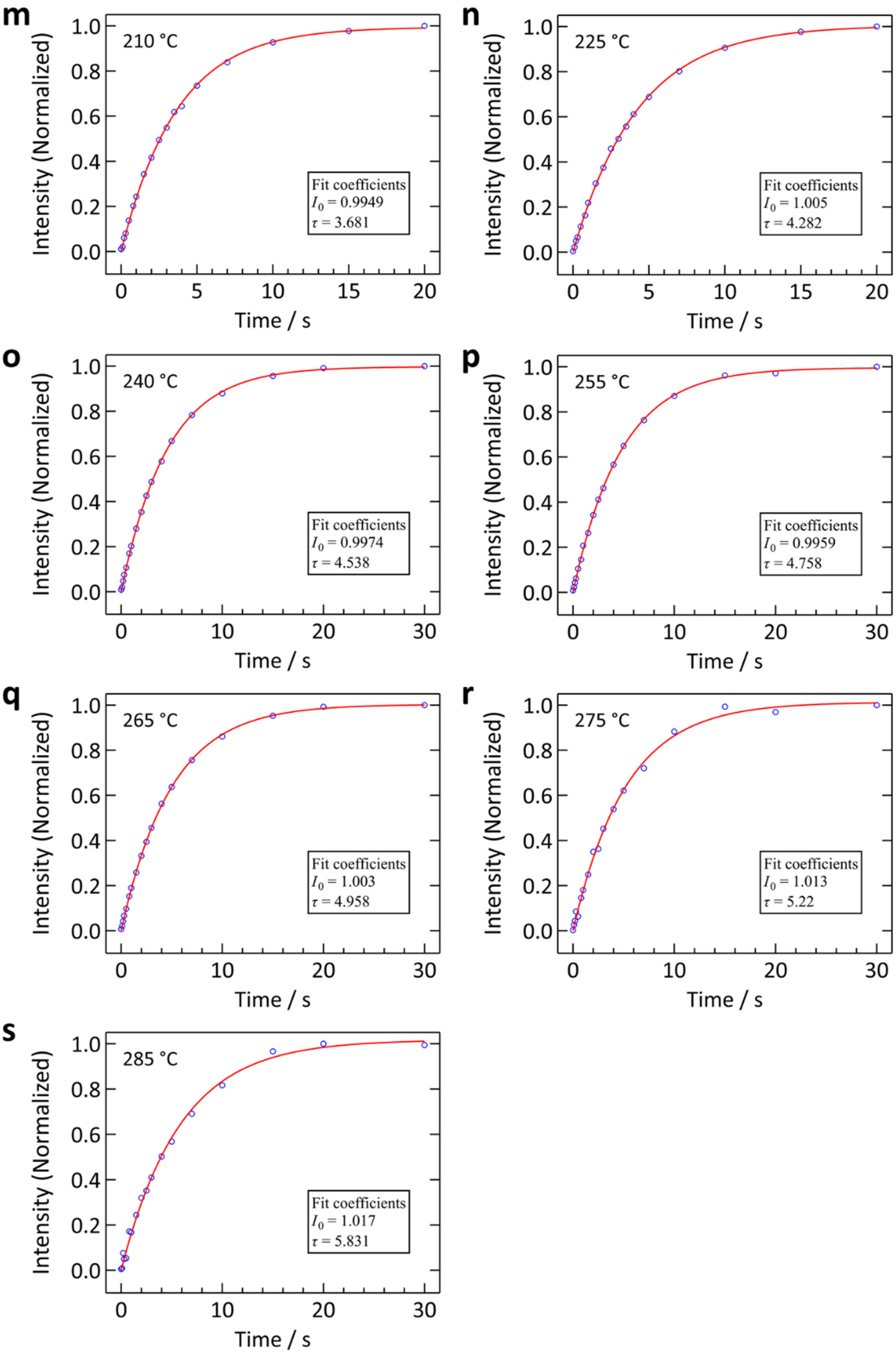

Figure S49 (continued). T_1 relaxation measured by ^{19}F solid-state NMR for **TK-COF-P** at different temperatures and the curve fit by equation S7. The temperature is shown in each panel. See Figure S52b for the temperature dependence of T_1.

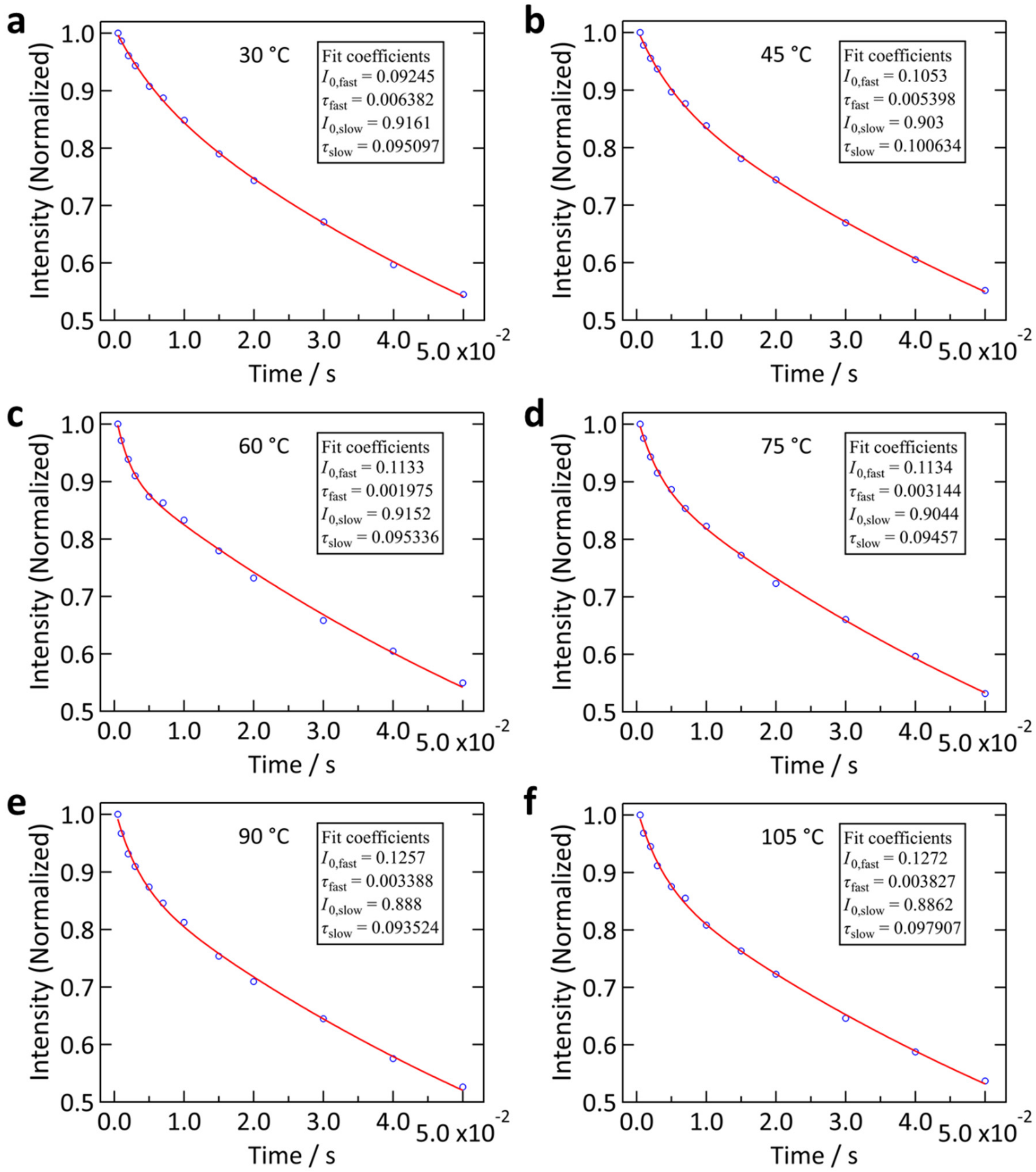

Figure S50. $T_{1\rho}$ relaxation measured by ^{19}F solid-state NMR for **TK-COF-P** at different temperatures and the curve fit by equation S8. The temperature is shown in each panel. See Figure S54b for the temperature dependence of $T_{1\rho}$.

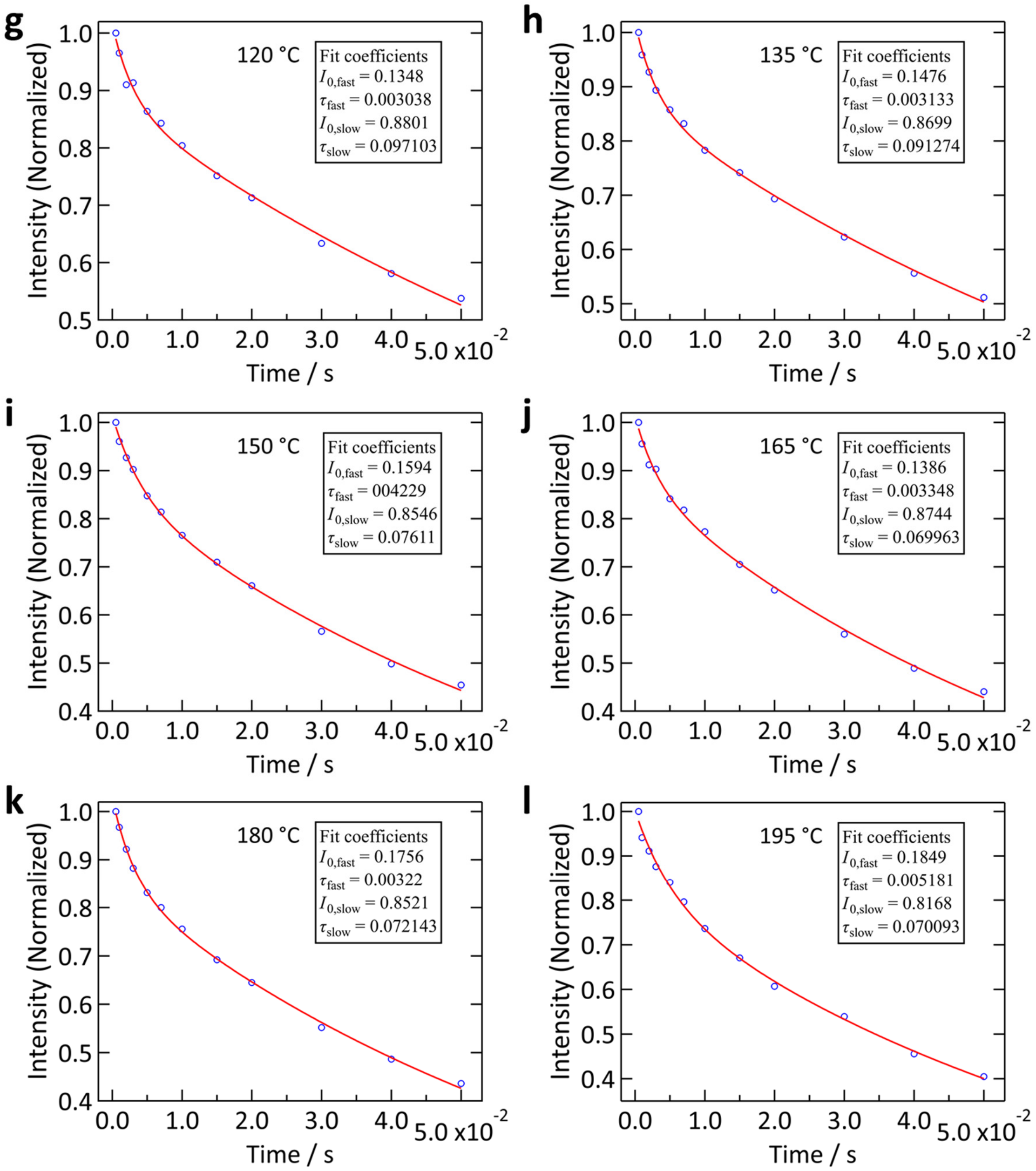

Figure S50 (continued). $T_{1\rho}$ relaxation measured by ^{19}F solid-state NMR for **TK-COF-P** at different temperatures and the curve fit by equation S8. The temperature is shown in each panel. See Figure S54b for the temperature dependence of $T_{1\rho}$.

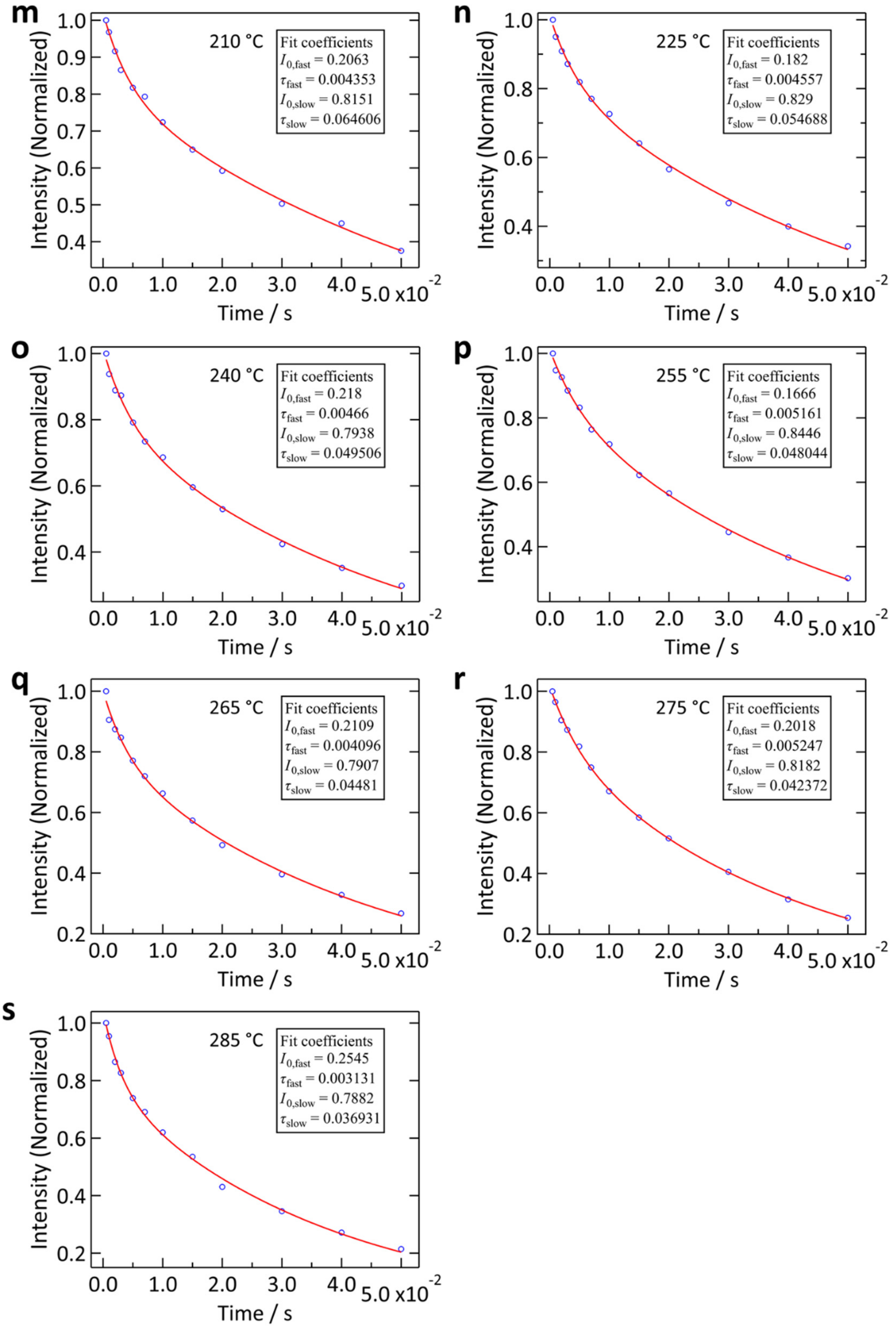

Figure S50 (continued). $T_{1\rho}$ relaxation measured by ^{19}F solid-state NMR for **TK-COF-P** at different temperatures and the curve fit by equation S8. The temperature is shown in each panel. See Figure S54b for the temperature dependence of $T_{1\rho}$.

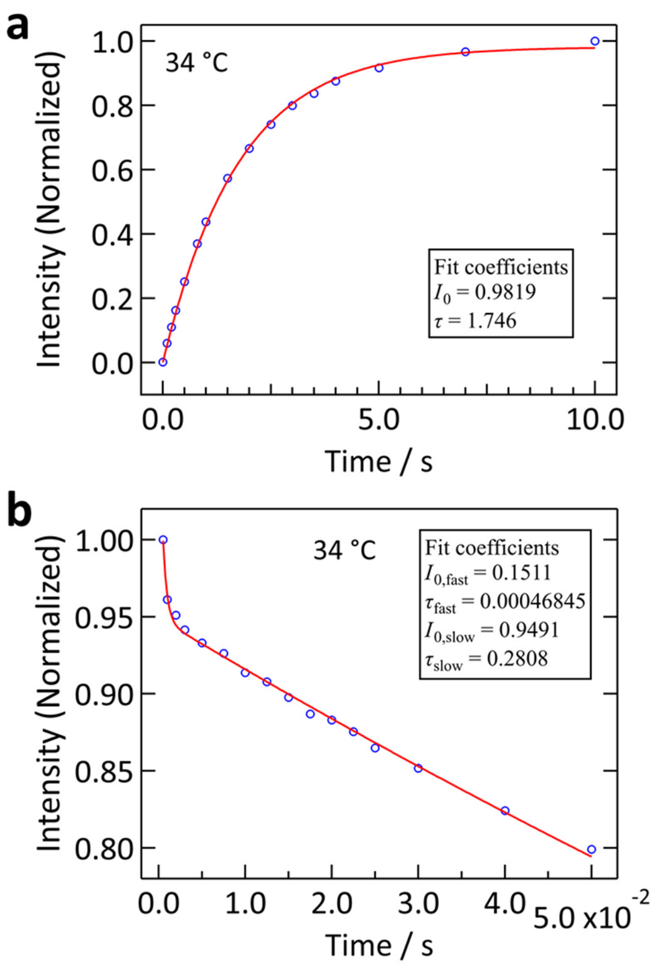

Figure S51. (a) T_1 and (b) $T_{1\rho}$ relaxations measured by ^{19}F solid-state NMR for **HABF** at 34 °C and the curve fits by equations S7 and S8, respectively.

S7.3 Temperature dependences of T_1 and $T_{1\rho}$

Temperature dependences of ^{19}F T_1 are shown in Figure S52 for **TK-COF-M** and **TK-COF-P**. Temperature dependences of the fit parameters (τ_{fast}, τ_{slow}, A_{fast}, and A_{slow}) are shown in Figure S53 for **TK-COF-M** and **TK-COF-P**. Temperature dependences of ^{19}F $T_{1\rho}$ are shown in Figure S54 for **TK-COF-M** and **TK-COF-P**.

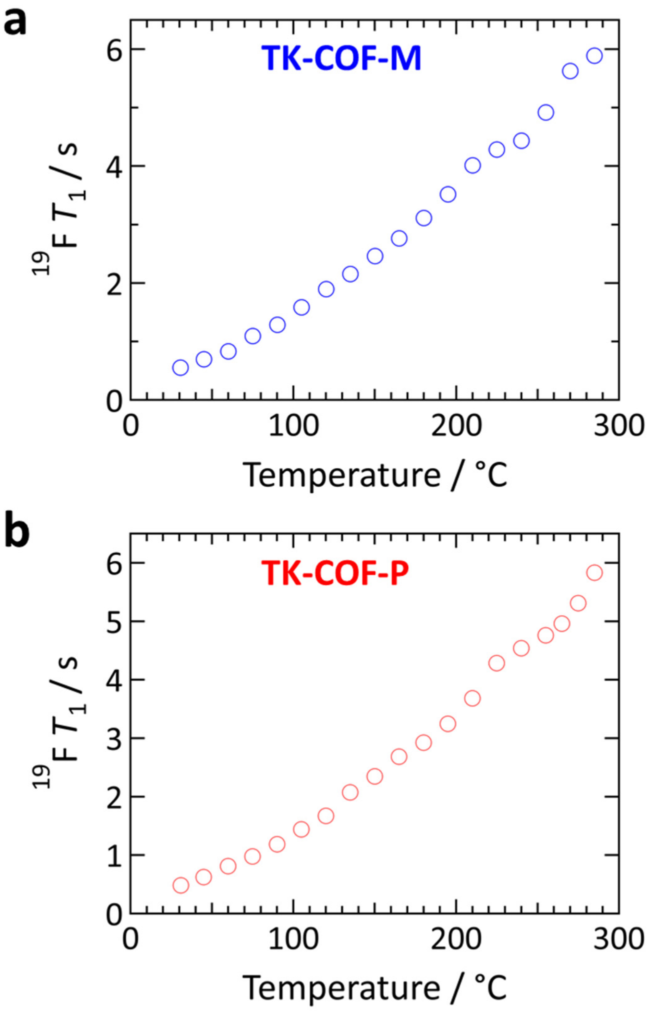

Figure S52. Temperature dependences of ^{19}F T_1 for (a) **TK-COF-M** and (b) **TK-COF-P**.

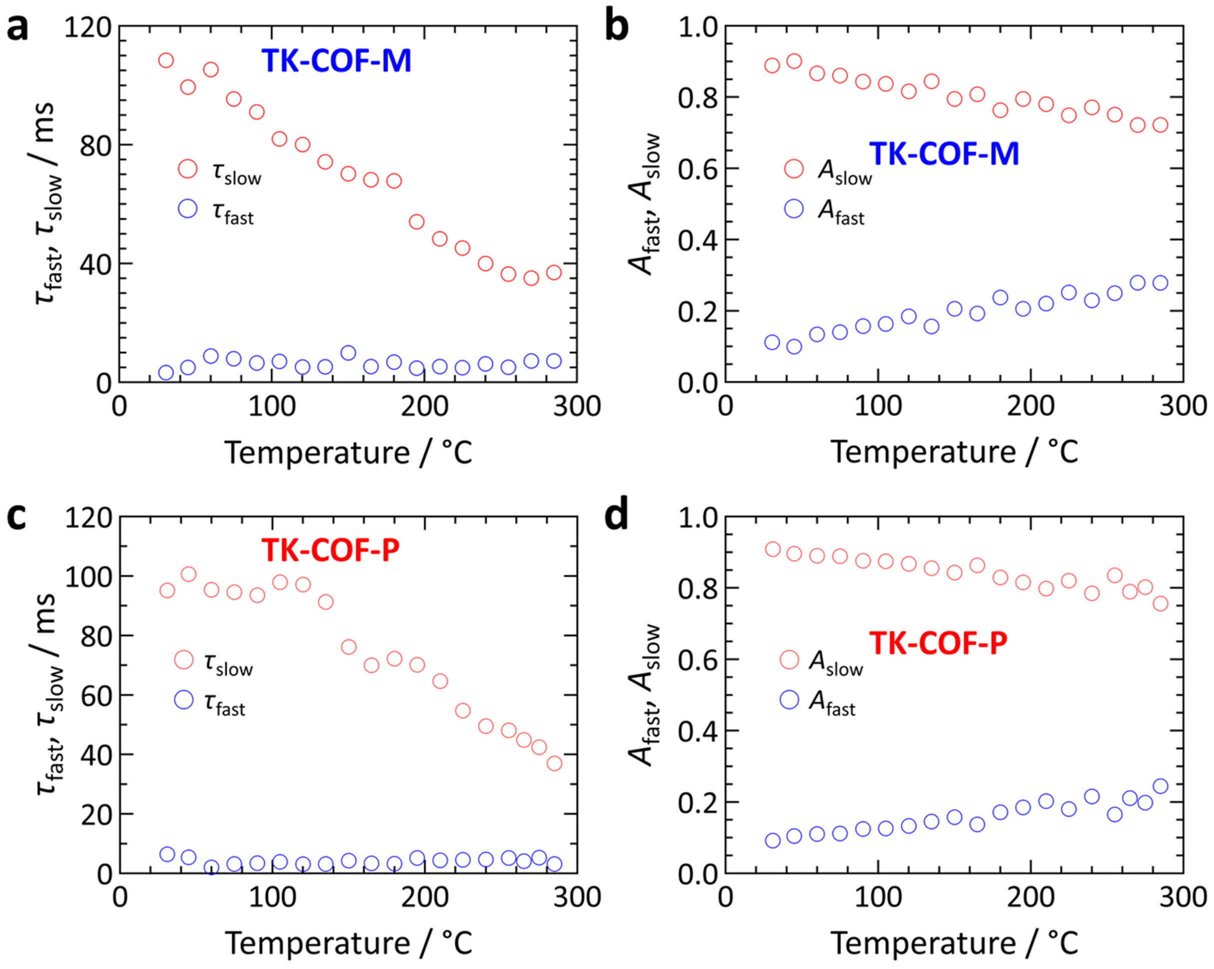

Figure S53. Temperature dependences of (a) τ_{fast} and τ_{slow} and (b) A_{fast} and A_{slow} for **TK-COF-M**, and (c) τ_{fast} and τ_{slow} and (d) A_{fast} and A_{slow} for **TK-COF-P**.

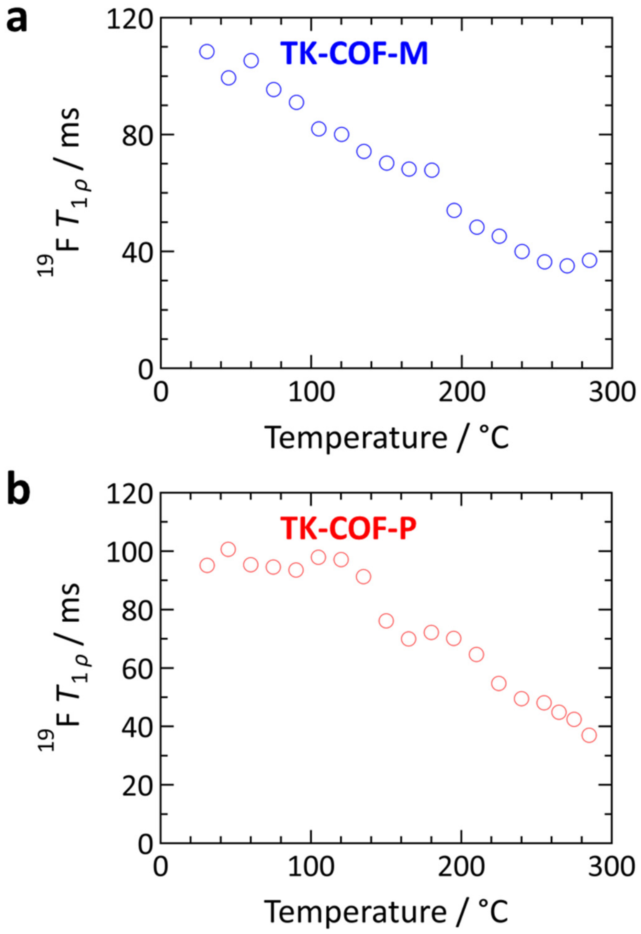

Figure S54. Temperature dependences of ^{19}F $T_{1\rho}$ for (a) **TK-COF-M** and (b) **TK-COF-P**.

The plots of ^{19}F $T_{1\rho}^{-1}$ vs. T^{-1} for **TK-COF-P** and **TK-COF-M** are shown in Figures S55a and S55b below, respectively. The data in Figure S55b are the same as those presented in Figure 3g in the main text. Note the quantitatively similar values of $T_{1\rho}^{-1}$ for both COFs.

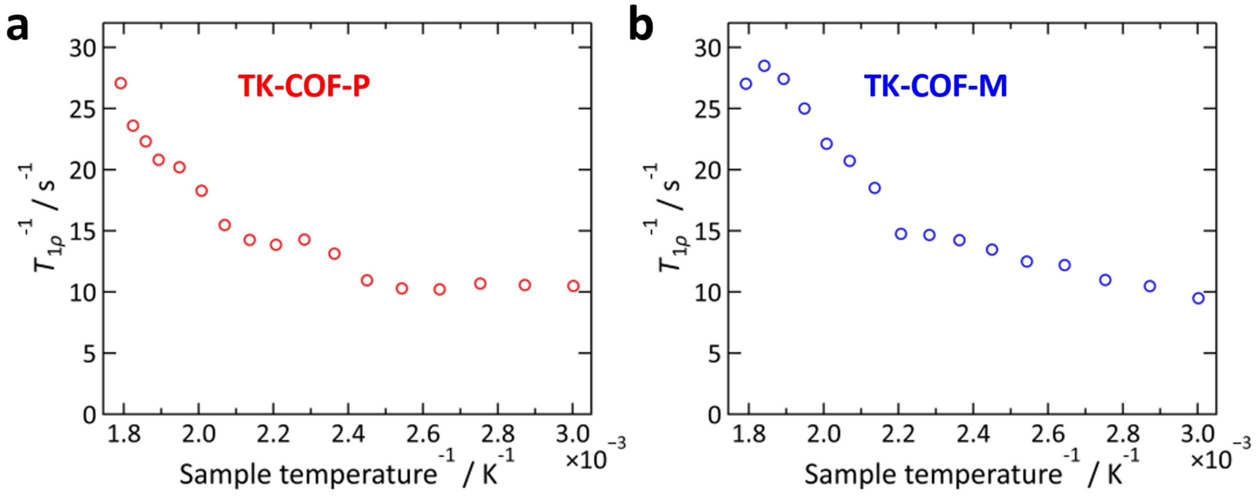

Figure S55. The data of $T_{1\rho}^{-1}$ vs. T^{-1} for (a) **TK-COF-P** and (b) **TK-COF-M**. The data in panel (b) are the same as those presented in Figure 3g in the main text.

S7.4 Discussion of the temperature dependence of ^{19}F NMR signal linewidth

Figure S56a presents the solid-state ^{19}F NMR spectra of **TK-COF-M** at different temperatures, showing that the temperature dependence is not remarkable. Figure S56b presents the magnification of the main peak area. As shown, a narrowing of the full-width at half maximum (FWHM) from 8.5 ppm at 30 °C to 7.7 ppm at 285 °C was found; this is interpreted as the motional narrowing of the solid-state NMR peak induced by the onset of the flipping motion of the dipolar DFP rotors at elevated temperatures, consistent with the results of the temperature-dependent dielectric response measurements shown in Figure 3e in the main text.

However, the extent of the reduction of the peak width (*i.e.*, $(7.7 - 8.5)/8.5 \times 100 \cong -10\%$) from room temperature to 285 °C seems not to be remarkable. We consider that the following two reasons are the main contributors to this non-remarkable peak narrowing of the ^{19}F NMR spectra.

The first reason is related to the fact that the measurement was done on the dried COFs. Although the drying of (*i.e.*, removal of solvent from) the present COFs did not affect the integrity of the COFs, as has been evidenced by the FT-IR spectra (Figure 1d in the main text), the solid-state ^{13}C CP/MAS NMR spectra (Figure 1e in the main text), and the elemental analysis (Table S3), all of which were conducted on the dried samples, the removal of the solvent from the present COFs weakened the PXRD patterns (Figure S16), which indicates the reduction of the structural order and hence microscopic uniformity. In other words, *after the removal of solvent or drying, the F atoms comprising the DPF rotor were placed in different micro-environments*. This nonuniformity of the environments surrounding the F atoms is considered to have caused the non-uniform broadening of the peaks of the NMR spectra. Therefore, the amount of the reduction in the FWHM ($\Delta FWHM = 7.7 - 8.5 = -0.8$ ppm) is considered to have been overwhelmed by the nonuniform width that had existed in the present dried COF samples.

The second probable reason is that the rotational motion of the present DFP rotors is considered to be sluggish “flipping” rather than fast free rotation, even at elevated temperatures above 200 °C; this viewpoint has been strongly suggested by our results of the temperature-dependent dielectric spectroscopy (Figure 3e in the main text) and the large activation energy barrier to flipping (24 kcal mol^{-1}, Figure 3f in the main text). Therefore, this second reason could also be responsible for the non-remarkable spectral narrowing of the ^{19}F NMR peaks presented in Figure S56.

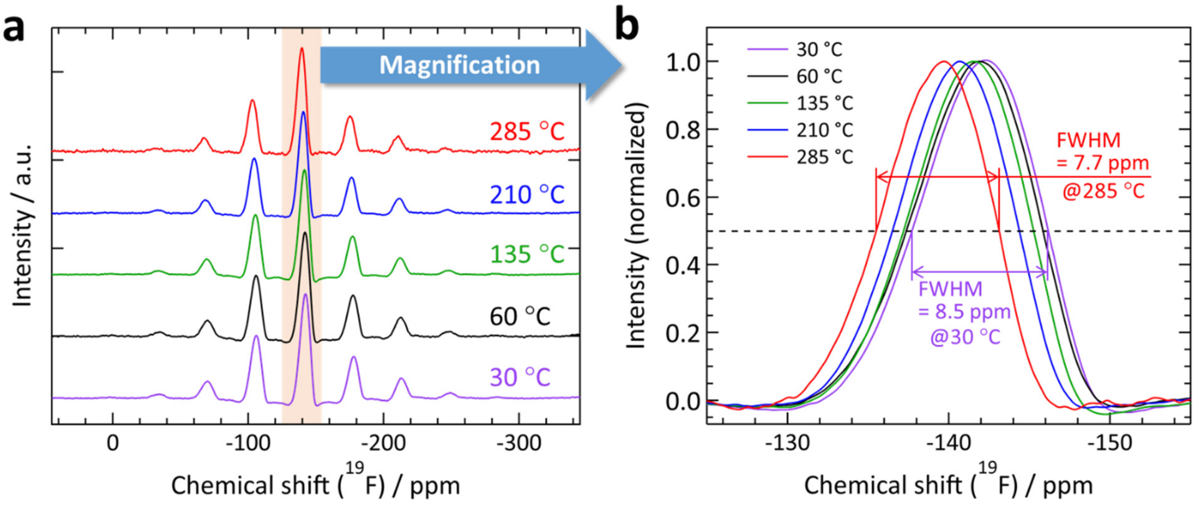

Figure S56. (a) Comparison of the solid-state ^{19}F NMR spectra of **TK-COF-M** at selected temperatures and (b) comparison of the full-width at half maximum (FWHM) of the main peak between 30 and 285 °C.

S7.5 Procedure of Kubo–Tomita analysis and obtained fit parameters

The experimental data of the temperature dependence of $T_{1\rho}^{-1}$ measured for **TK-COF-M** were fit by the Kubo–Tomita function[S17-S19]

$$T_{1\rho}^{-1} = C[\tau_c(1+\omega_0^2\tau_c^2)^{-1} + 4\tau_c(1+4\omega_0^2\tau_c^2)^{-1}] \tag{S10}$$

$$\tau_c^{-1} = \tau_0^{-1}\exp\left(\frac{E_a}{RT}\right), \tag{S11}$$

where ω_0, τ_0, R, and T are the angular frequency (the spin-lock frequency used, *ca*. 65 kHz for the measurement of **TK-COF-M**), the pre-exponential factor, the gas constant (8.3145 J K^{-1} mol^{-1}), and the temperature of the measurement, respectively; C, τ_0, and E_a (activation energy) are fit parameters. The fits to the data were done using the least-squares method. The quality of the fit was evaluated by the coefficient of determination (R^2).

Parameters in the Kubo–Tomita function (equations S10 and S11) obtained from the fit to the data of $T_{1\rho}^{-1}$ for **TK-COF-M** (Figure 3g in the main text) are summarized in Table S12 below.

Table S12. Fit parameters from the Kubo–Tomita functions for the $T_{1\rho}$ data (**TK-COF-M**)

	C / s^{-2}	τ_0 / s	E_a / kcal mol^{-1}	Coefficient of determination (R^2)
Higher temperature component, H	5.10×10^6	1.02×10^{-1}	12.1	–
Lower temperature component, L	3.28×10^6	3.83×10^{-5}	2.64	–
Total fit curve (= H + L)	–	–	–	0.994

References

[S1] Wang, X.; Enomoto, R.; Murakami, Y. Ionic Additive Strategy to Control Nucleation and Generate Larger Single Crystals of 3D Covalent Organic Frameworks. *Chem. Commun.* **2021**, *57*, 6656–6659.

[S2] Chérif, S. F.; Chérif, A.; Dridi, W.; Zid, M. F. Ac Conductivity, Electric Modulus Analysis, Dielectric Behavior and Bond Valence Sum Analysis of $Na_3Nb_4As_3O_{19}$ Compound. *Arab. J. Chem.* **2020**, *13*, 5627–5638.

[S3] Pallach, R.; Keupp, J.; Terlinden, K.; Frentzel-Beyme, L.; Kloß, M.; Machalica, A.; Kotschy, J.; Vasa, S. K.; Chater, P. A.; Sternemann, C.; Wharmby, M. T.; Linser, R.; Schmid, R.; Henke, S. Frustrated Flexibility in Metal-Organic Frameworks. *Nat. Commun.* **2021**, *12*, 4097.

[S4] Nguyen, H. L. Reticular Design and Crystal Structure Determination of Covalent Organic Frameworks. *Chem. Sci.* **2021**, *12*, 8632–8647.

[S5] Reticular Chemistry Structure Resource. http://rcsr.net/ (accessed 2025-06-13).

[S6] Pawley, G. S. Unit-cell Refinement from Powder Diffraction Scans. *J. Appl. Crystallogr.* **1981**, *14*, 357–361.

[S7] Rietveld, H. M. A Profile Refinement Method for Nuclear and Magnetic Structures. *J. Appl. Crystallogr.* **1969**, *2*, 65–71.

[S8] Rappe, A. K.; Casewit, C. J.; Colwell, K. S.; Goddard, W. A. I.; Skiff, W. M. UFF, a Full Periodic Table Force Field for Molecular Mechanics and Molecular Dynamics Simulations. *J. Am. Chem. Soc.* **1992**, *114*, 10024–10035.

[S9] Du, Y.; Calabro, D.; Wooler, B.; Li, Q.; Cundy, S.; Kamakoti, P.; Colmyer, D.; Mao, K.; Ravikovitch, P. Kinetic and Mechanistic Study of COF-1 Phase Change from a Staggered to Eclipsed Model upon Partial Removal of Mesitylene. *J. Phys. Chem. C* **2014**, *118*, 399−407.

[S10] Dey, A.; Chand, S.; Alimi, L. O.; Ghosh, M.; Cavallo, L.; Khashab, N. M. From Capsule to Helix: Guest-Induced Superstructures of Chiral Macrocycle Crystals. *J. Am. Chem. Soc.* **2020**, *142*, 15823−15829.

[S11] Attia, A. A.; Soliman, H. S.; Saadeldin, M. M.; Sawaby, K. AC Electrical Conductivity and Dielectric Studies of Bulk *p*-Quaterphenyl. *Synth. Met.* **2015**, *205*, 139−144.

[S12] Mansingh, A.; Dhar, A. The AC Conductivity and Dielectric Constant of Lithium Niobate Single Crystals. *J. Phys. D: Appl. Phys.* **1985**, *18*, 2059.

[S13] Thurber, K. R.; Tycko, R. Measurement of Sample Temperatures under Magic-Angle Spinning from the Chemical Shift and Spin-Lattice Relaxation Rate of ^{79}Br in KBr Powder. *J. Magn. Reson.* **2009**, *196*, 84–87.

[S14] Hahn, E. L. Spin Echoes. *Phys. Rev.* **1950**, *80*, 580–594.

[S15] Freeman, R.; Hill, H. D. W. Fourier Transform Study of NMR Spin–Lattice Relaxation by "Progressive Saturation." *J. Chem. Phys.* **1971**, *54*, 3367–3377.

[S16] Chen, Q.; Schmidt-Rohr, K. ^{19}F and ^{13}C NMR Signal Assignment and Analysis in a Perfluorinated Ionomer (Nafion) by Two-Dimensional Solid-State NMR. *Macromolecules* **2004**, *37*, 5995–6003.

[S17] Kubo, K.; Tomita, K. A General Theory of Magnetic Resonance Absorption. *Phys. Soc. Jpn.* **1954**, *9*, 888–919.

[S18] Vogelsberg, C. S.; Uribe-Romo, F. J.; Lipton, A. S.; Yang, S.; Houk, K. N.; Brown S.; Garcia-Garibay. M. A. Ultrafast Rotation in an Amphidynamic Crystalline Metal Organic Framework. *Proc. Natl. Acad. Sci.* **2017**, *114*, 13613–13618.

[S19] Perego, J.; Bezuidenhout, C. X.; Bracco, S.; Piva, S.; Prando, G.; Aloisi, C.; Carretta, P.; Kaleta, J.; Le, T. P.; Sozzani, P.; Daolio, A.; Comotti, A. Benchmark Dynamics of Dipolar Molecular Rotors in Fluorinated Metal-Organic Frameworks. *Angew. Chem. Int. Ed.* **2023**, *62*, e202215893.
